# Chemoselective Difluorination of Tetramic Acids in Water

**DOI:** 10.1021/acsomega.5c12048

**Published:** 2026-03-30

**Authors:** M. M. Swatscheno, R. Mahawongnan, Itamar Blau, Jacob S. Tracy

**Affiliations:** Department of Chemistry, 6491 University of West Florida, Pensacola, Florida 32514, United States

## Abstract

3,3-Difluorotetramic acids are key building blocks for the construction of medicinally relevant compounds. However, general methods for their synthesis via the direct electrophilic fluorination of tetramic acids are lacking. Herein we report such a method for the direct 3,3-difluorination of tetramic acids in water without any organic cosolvents utilizing Selectfluor as the electrophilic fluorine source. This reaction shows high chemoselectivity across a range of functional groups, including alkenes, alkynes, ketones, and aromatic rings, and tolerates a range of common nitrogen protecting groups. Finally, the reaction was successfully run at the multimillimole scale and was shown to tolerate an air atmosphere.

## Graphical abstract

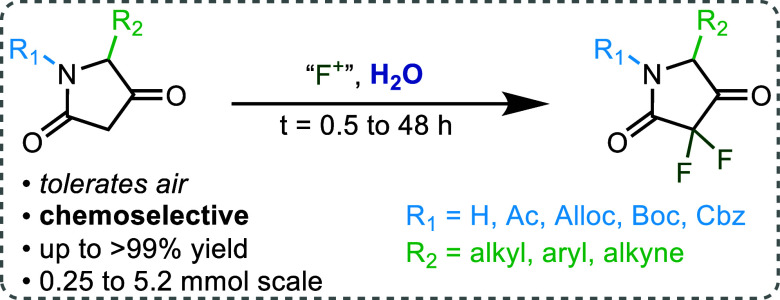

## Introduction

Tetramates are a class of natural compounds based upon a tetramic acid (pyrrolidine-2,4-dione) core that are commonly encountered in both marine and terrestrial environments as secondary metabolites.^1^ These natural products have been extensively studied for their antibacterial,^2^ antifungal,^3^ herbicidal,^4^ antiviral (HIV),^5^ antitumor,^6^ and cytotoxic properties.^7^ The pharmaceutical industry has also shown an interest in this motif, and in recent years investigational drug candidates built around tetramic acid cores have been disclosed for antibiotic activity (**1**)^8^ and for the treatment of type 2 diabetes (**2**).^9^ Additional investigational drugs based upon the closely related 4-hydroxyl-2-pyrrolidone core have also been explored as treatments for kidney disease (**3**),^10^ as nootropics (**4**),^11^ and as anticancer agents (**5**, Figure 1).^12^

**Figure 1:**
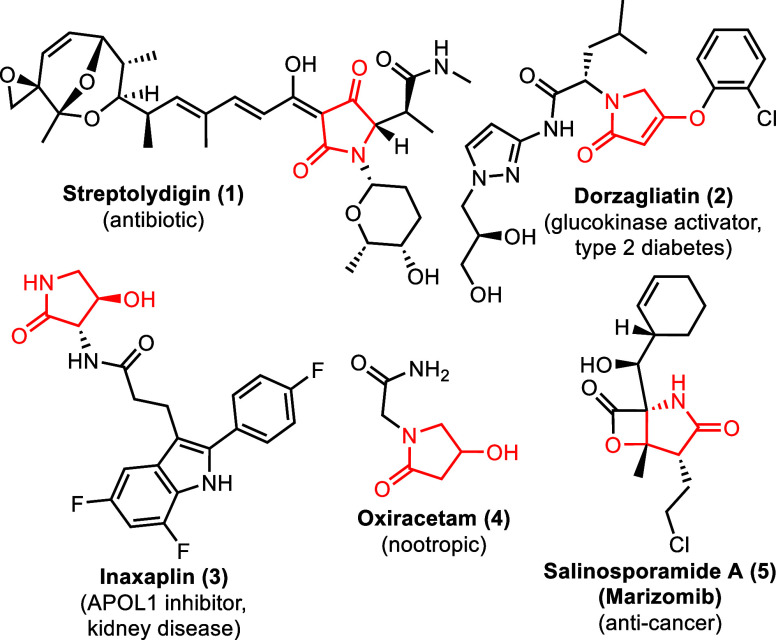
Investigational drugs based upon the tetramic acid and related 4-hydroxyl-2-pyrrolidone cores.

Interest in the biological properties of tetramic acid derivatives has spurred efforts in the synthetic community to develop methods for their synthesis^13^ and structural elaboration.^14^ Despite this work, at the onset of this project there were no reported methods for the 3,3-difluorination of tetramic acids. Such a transformation would be desirable as the introduction of geminal difluorines^15^ into molecules can impact pharmacokinetic and physiochemical properties including the p*K*_a_ of nearby functional groups,^16^ lipophilicity,^17^ solubility,^18^ metabolic stability,^19^ and molecular conformation.^20^ The two prior efforts to synthesize 3,3-difluorinated tetramic acids in the literature required the introduction of fluorine at an early stage in the synthesis followed by multistep sequences to construct the tetramic acid motif.^21^ For example, Eli Lilly and Company’s synthesis relied upon a five-step sequence starting from a doubly protected amino alcohol that utilized both a rhodium catalyst and four equivalents of diethylzinc (Scheme 1).^22^

**Scheme 1:**
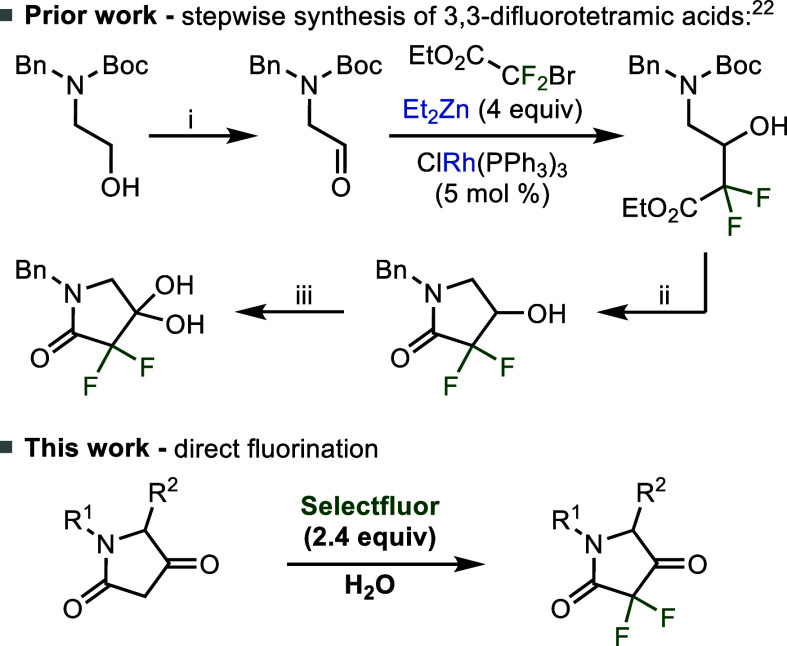
Prior Synthesis of 3,3-Difluorotetramic Acids at the Onset of this Project Versus this Work^a^

a (i): (COCl)_2_, DMSO, NEt_3_, CH_2_Cl_2_. (ii): HCl, *i*PrOH then Na_2_CO_3_. (iii): Potassium 2-iodo-5-methylbenzenesulfonate, oxone.

We therefore sought to develop a set of reaction conditions for the direct 3,3-difluorination of tetramic acids, with the specific goal of utilizing a green solvent such as water to minimize economic and environmental impacts.^23^ The desire to develop an aqueous set of conditions represented a particular challenge for the targeted transformation. While many methods have been developed for the difluorination of nontetramic acid 1,3-dicarbonyls,^24^ very few of these have been reported to take place with water as the sole solvent and all such examples required elevated reaction temperatures.^25^ Since this project was initiated, a few scattered examples have appeared in the patent literature showcasing the direct difluorination of tetramic acids for use as building blocks for hypoxia-inducible factor-2α (HIF-2α) inhibitors.^26^ However, these examples rely upon acetonitrile as a solvent and their generality has not been demonstrated.

## Results and Discussion

We began our studies with tetramic acid **6**, readily available from the *tert*-butoxycarbonyl (Boc) protected amino acid *N-*Boc-phenylalanine (see Supporting Information).^13g^ Treatment of this substrate with 2.0 equiv of *N*-fluorobenzenefulonimide (NFSI) as the electrophilic fluorine source in either MeCN or DMF (0.25 M) for 24 h at room temperature resulted in low conversions, forming less than 5% of the desired 3,3-difluorinated product **7** (Table 1, entries 1–2). These solvents were selected for the initial experiments as they have a well-documented history of being successfully employed in electrophilic fluorination reactions of other 1,3-dicarbonyl derivatives.^24^ Seeing little reactivity under neutral conditions that rely upon formation of the enol tautomer of **6** for reactivity, we sought to improve yield by inducing formation of the more nucleophilic enolate through the addition of 2.5 equiv of sodium bicarbonate in DMF. Under these basified conditions, 44% of the desired product **7** was isolated, albeit after a lengthy 96 h reaction time (entry 3). Still observing sluggish reactivity, we turned our attention to the more electrophilic Selectfluor (1-(chloromethyl)-4-fluoro-1,4-diazabicyclo[2.2.2]­octane bis­(tetrafluoroborate)).^27^ Use of Selectfluor in DMF resulted in 41% of the desired product in just 24 h and without the need for any exogenous base (entry 4).

**Table 1:**
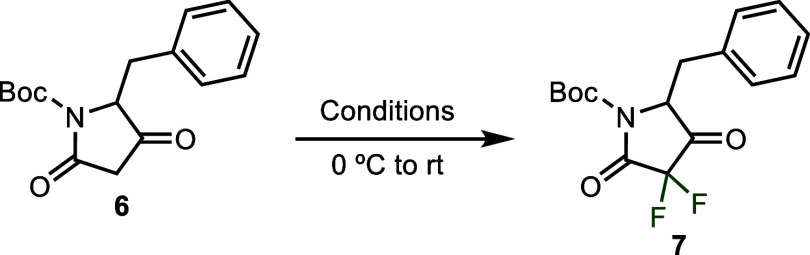
Optimization of the Difluorination of Tetramic Acid Derivatives^a^

entry	fluorine source (“F^+^”)	equiv of “F^+^”	solvent	yield of 7^b^
1	NFSI	2.0	MeCN	<5%
2	NFSI	2.0	DMF	<5%
3^c^	NFSI	2.0	DMF	44%
4	Selectfluor	2.0	DMF	41%
5	Selectfluor	2.0	H_2_O	55%
6	Selectfluor	2.2	H_2_O	76%
**7**	**Selectfluor**	**2.4**	**H_2_O**	**82%**
8	NFSI	2.0	H_2_O	11%
9	Selectfluor	2.0	EtOH	10%
10^d^	Selectfluor	1.0	H_2_O	44%

a Reaction performed under a nitrogen atmosphere at 0.25 mmol scale for 19–25 h at 0.25 M. Entries 1 and 2 for 24 h, entry 4 and 7 for 22 h, entry 5 and 9 for 21 h, entry 6 for 25 h, and entry 8 for 19 h.

b Yields are isolated yields.

c 2.5 equiv of NaHCO_3_, 96 h.

d 44 h.

With promising levels of reactivity, we next sought to find a green solvent that would function with our tetramic acid substrates. Replacing DMF with water resulted in a heterogeneous reaction mixture but effectively promoted the desired transformation with an improved yield of 55% (entry 5). Increasing the equivalents of Selectfluor further improved yield, reaching 82% with 2.4 equiv (entries 6–7). Comparing the reactivity between Selectfluor and NFSI in water showed Selectfluor once again to be the superior electrophilic fluorine source (entry 8). Use of ethanol, another commonly employed green solvent,^28^ was not promising and resulted in just 10% yield (entry 9). Finally, the inherent selectivity for monofluorination versus difluorination was tested by utilizing just 1 equiv of Selectfluor in water for an extended reaction time of 44 h. Under these conditions, high chemoselectivity was observed for difluorination with product **7** isolated in 44% yield relative to the amount of starting tetramic acid **6** and accounting for 88% of total fluorine (entry 10). This selectivity contrasts with that observed with other 1,3-dicarbonyls, where monofluorination generally predominates when 1 equiv of electrophilic fluorine is used.^24^ In all cases, high chemoselectivity was also observed for fluorination at the heavily activated 3-position with no evidence of significant fluorine incorporation at the 5-position. This is despite the enolizability of the 5-position by virtue of the ketone at the 4-position coupled with further acidification by the electron-deficient nitrogen at the 1-position and gem-difluorination of the product at the 3-position.

With optimized conditions in hand (Table 1, entry 7), we turned our attention to exploring the scope of the reaction (Scheme 2). A variety of functional groups were found to be tolerated at the 5-position including simple alkyl groups (**8**), alkynes (**9**), and protected amines (**10**). In the case of the terminal alkyne (**9**), there was no evidence of fluorine incorporation into the π-system nor the terminus of the alkyne. Aromatic rings directly attached to the 5-position (**11**) were also well tolerated with no fluorination of the arene nor the 5-position of the tetramic acid. Lack of fluorination at the 5-position is particularly noteworthy in this case, as the enolizable proton at that position is further acidified by the aromatic ring that would be conjugated to the ketone upon enolization. Substitution at the 5-position with a 4-nitrobenzyl (**12**) group required a prolonged reaction time of 48 h to achieve a satisfactory yield, likely due to a combination of low substrate solubility and the tendency for this tetramic acid starting material to clump together in water, thereby minimizing solvent-exposed surface area. The lengthy reaction time resulted in significant amounts of Boc group hydrolysis, and the final product was isolated as a 9:1 ratio of Boc deprotection to Boc retention.

**Scheme 2:**
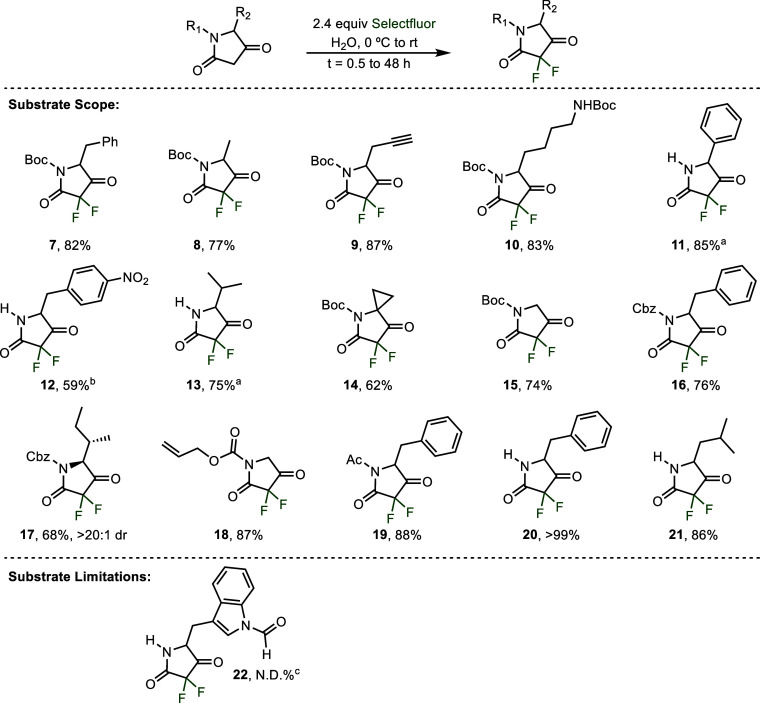
Scope of the 3,3-Difluorination of tetramic Acid Derivatives^a^

a Reactions run at 0.25 mmol scale for 0.5-48 h. Yields are isolated yields. [a] Boc removal was observed under the reaction conditions. [b] Product isolated as a 9:1 ratio of Boc removal to Boc retention. [c] Yield was not determined (N.D.) because of decomposition.

Reaction efficacy was not strongly sensitive to steric bulk at the 5-position, as branched alkyl groups (**13**) and 5,5-dialkyl substitution (**14**) were both tolerated. In addition, it was shown that substitution at the 5-position of the tetramic acid was not required for reactivity and tetramic acids with a methylene at this position proved to be excellent substrates (**15**).

Finally, a range of common nitrogen protecting groups could be employed under the reaction conditions including the carboxybenzyl (Cbz, **16** and **17**), allyloxycarbonyl (Alloc, **18**), and acetyl (Ac, **19**) protecting groups. Unprotected tetramic acids featuring a free N–H were likewise effective substrates in the reaction (**20** and **21**). In the case of the Alloc group, the alkene π-system remained intact with no fluorine incorporation. Product **17**, derived from chiral Cbz-l-isoleucine also points to the mild nature of these reaction conditions, as only a single diastereomer of product was observed, indicating that enolization at the 5-position is unlikely to be occurring and that stereochemistry at the 5-position is likely retained under our reaction conditions. This is further supported by our previous observations of no fluorine incorporation at the 5-position.

While many functional groups were tolerated, the presence of an electron-rich indole ring (**22**) was not. This substrate resulted in high reactivity but yielded a complex mixture of products with many more fluorine peaks observed in the crude ^19^F NMR than expected, likely indicating competitive fluorination at the 2 and/or 3-positions of the indole ring that ultimately lead to decomposition.

In cases where a Boc protecting group was coupled with a relatively large phenyl (**11**) or isopropyl group (**13**) at the 5-position, removal of the Boc protecting group was observed under the reaction conditions. This phenomenon is potentially explained by increased steric strain between the Boc group and the bulky 5-substituent that is alleviated upon Boc hydrolysis. This is further supported by observations that the sterically smaller Cbz group does not undergo such a cleavage when coupled with a similarly bulky isobutyl group at the 5-position (**17**). However, we cannot definitively rule out differences in water solubility between Boc and Cbz-protected fluorinated products playing a role here.

Smaller substrates with proportionally higher polar surface areas were found to react faster under the reaction conditions. This enhanced rate of reactivity is believed to result from higher starting material solubility in water, likely in the enol tautomer. The theory of enhanced solubility is further supported by the observation that these fluorinated products, likely in the hydrate form, were often fully soluble under the aqueous reaction conditions. For example, product **18** was formed in just 0.67 h and resulted in a completely homogeneous reaction mixture. However, neither high levels of starting material nor product solubility were required, and heterogeneous mixtures were well tolerated, often resulting in very high levels of conversion. In these cases, strong stirring was often found to be beneficial.

When fast reactivity was coupled with steric bulk at the 5-position, reaction time could be used to control the retention or deprotection of the tetramic acid’s Boc group under the reaction conditions. For example, running the difluorination of **23** for 1 h resulted in 62% yield of the difluorination product with the Boc group intact (**14**) while running for 20 h resulted in a 70% isolated yield of the deprotected product **24** (Scheme 3). The modest yield of **14** after 1 h was in part accounted for by the presence of approximately 17% of the deprotected product **24** as determined by ^19^F crude NMR (see Supporting Information Figure S62).

**Scheme 3:**
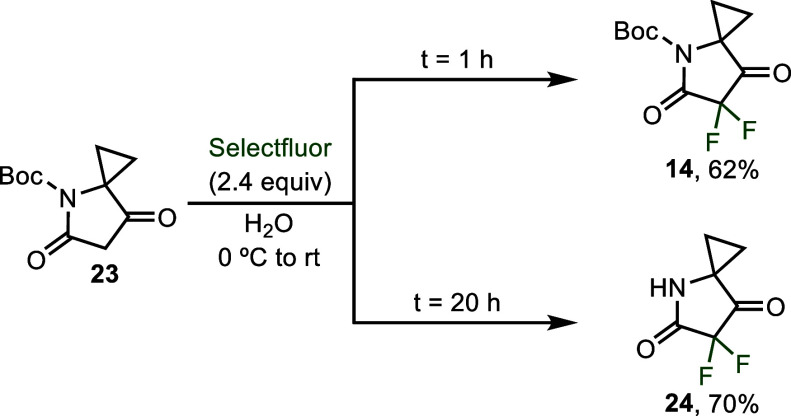
Use of Reaction time to Control the Retention or Deprotection of the Boc Group

To demonstrate the operational simplicity and robustness of these reaction conditions, we performed the difluorination reaction under an air atmosphere and saw only a minimal impact on yield (**7**, Scheme 4A). The reaction was also found to effectively scale utilizing traditional round-bottom flasks and PTFE stir bars, achieving a 90% isolated yield at a 2.0 mmol reaction scale and an 88% isolated yield at a 5.2 mmol scale that resulted in more than a gram of product (**7**, Scheme 4B,C). When performed at the larger gram-scale, the equivalents of Selectfluor could be reduced to just 2.14 equiv without impacting yield.

**Scheme 4:**
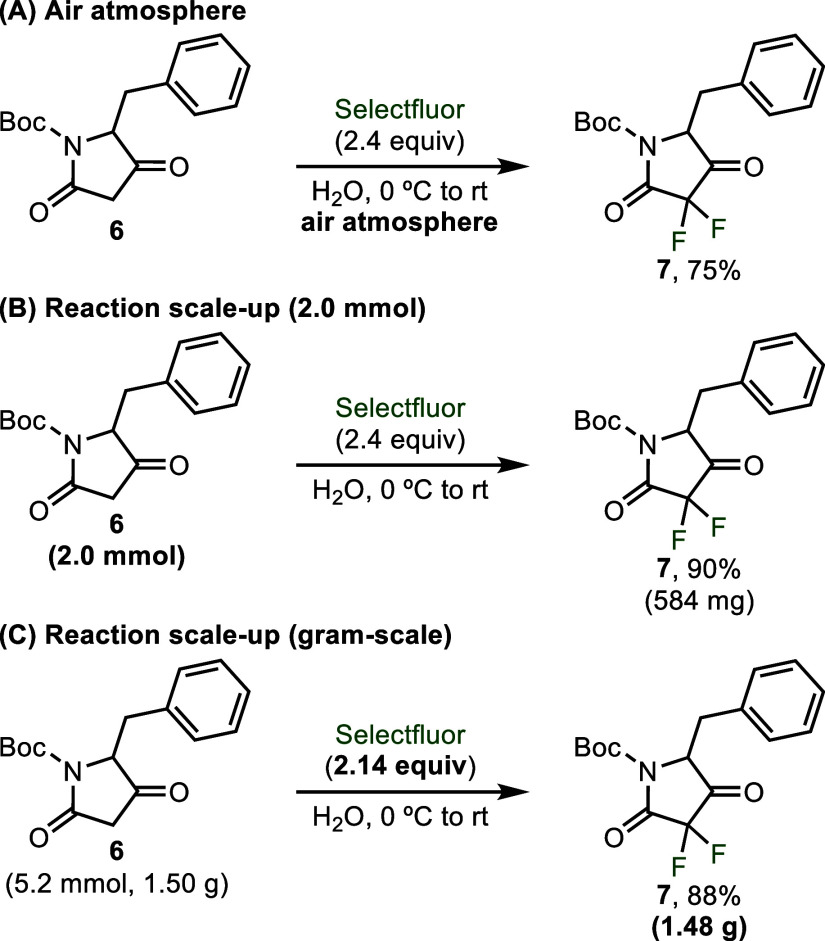
Tolerance of the Reaction Conditions to an Air Atmosphere (Part A) and the Successful Scale-Up of the Reaction to 2.0 mmol (Part B) and 5.2 mmol (Part C)

## Conclusion

We have developed a mild set of conditions for the chemoselective 3,3-difluorination of tetramic acid derivatives in water without the need for any organic cosolvents. The reaction conditions were found to be tolerant of an array of functional groups (alkenes, alkynes, ketones, aromatic rings), common nitrogen protecting groups (Boc, Cbz, Alloc, Ac), substitution patterns at the 5-position, and the presence of an air atmosphere. Reaction scales from 0.25 mmol up to 5.2 mmol were successfully demonstrated, with larger scales showing improvements in yield. Finally, in the case of Boc-protected tetramic acids, control of reaction time was shown to allow for either selective retention or removal of the protecting group.

## Materials and Methods

### Materials

Unless otherwise indicated, all reactions were carried out in glassware sealed with rubber septa, kept under a positive pressure of nitrogen from a balloon, and stirred with a PTFE-coated magnetic stir bar. All commercial reagents were used without further purification. Dry dichloromethane (DCM) was purchased in an AcroSeal bottle over molecular sieves and used as received. Deionized water was used for all reactions and workups. Solvents were transferred via stainless steel needles and plastic syringes. Reactions were monitored via thin layer chromatography (TLC) on Supelco glass backed TLC plates (250 μm thickness, 60 Å pore diameter, F-254 indicator) and visualized via UV irradiation (254 nm) and/or aqueous potassium permanganate solution followed by heating. Solvents were removed under reduced pressure with a rotatory evaporator and compounds dried under high vacuum on a Schlenk line. Manual flash chromatography was performed unless otherwise indicated and utilized glass columns pressurized by compressed air and silica gel (SiliCycle P60, 40–63 μm, 230–400 mesh). Automated flash chromatography was only performed when indicated and utilized a Biotage Selekt Enkel with self-packed columns (silica gel, SiliCycle P60, 40–63 μm, 230–400 mesh). In all cases, the eluting solvents, their corresponding ratios, and any linear gradients are listed individually for each compound.

### Synthetic Procedures: Synthesis of Tetramic Acids

All of the nitrogen-protected tetramic acid starting materials were synthesized based upon upon the reported procedure of Nisato and co-workers.^13g^

A flame-dried 100 mL round-bottom flask equipped with a magnetic stir bar was charged with the corresponding amino acid (1.0 equiv), Meldrum’s acid (2,2-dimethyl-1,3-dioxane-4,6-dione) (1.1 equiv), and DMAP (4-dimethylaminopyridine) (1.5 equiv) and placed under a nitrogen atmosphere. Dry DCM (0.17 M relative to the amino acid) was added, and the reaction was cooled to 0 °C (ice water bath). EDC•HCl (1-(3-(dimethylamino)­propyl)-3-ethylcarbodiimide hydrochloride) (1.5 equiv) was added and after several minutes the ice water bath was removed, after which the reaction mixture was stirred at room temperature for 2 h. The reaction was then cooled to 0 °C (ice bath), diluted with cold ethyl acetate (EtOAc) (100 mL per 5 mmol of amino acid) and quenched with cold brine (50 mL per 5 mmol of amino acid). The organic layer was then washed with cold 5% aqueous KHSO_4_ (3 × 100 mL per 5 mmol of amino acid) followed by brine (50 mL per 5 mmol of amino acid). The organic layer was then dried (Na_2_SO_4_), filtered, and concentrated under reduced pressure to an often-yellow residue. The residue, used without further purification, was placed under a nitrogen atmosphere, diluted with EtOAc (0.05 M), and heated at reflux for 30–60 min (oil bath, 90 °C). At that point, the reaction was cooled to room temperature, concentrated under reduced pressure, and purified via flash chromatography (SiO_2_) or recrystallization.

### Synthetic Procedures: Synthesis of Difluorinated Tetramic Acids

A 1-dram screw cap vial equipped with a magnetic stir bar was charged with the starting tetramic acid (1.0 equiv) and Selectfluor (2.4 equiv) then placed under a nitrogen atmosphere. The vial was cooled to 0 °C (ice water bath) and H_2_O (0.25 M) was then added. After 15 min, the ice water bath was removed and the reaction was allowed to stir at room temperature with strong stirring. The indicated times in the Supporting Information are total stirring times and include both within the ice water bath and at room temperature. At that point, the reaction was diluted with EtOAc (30 mL per 0.25 mmol of starting tetramic acid) and water (20 mL per 0.25 mmol of starting tetramic acid). The layers were separated and the aqueous layer was extracted with EtOAc (8 × 20 mL per 0.25 mmol of starting tetramic acid). The combined organic layers were then washed with brine (50 mL per 0.25 mmol of starting tetramic acid), dried (Na_2_SO_4_), filtered, and concentrated under reduced pressure. The resulting residues were purified via manual flash chromatography (SiO_2_) and loaded onto the column utilizing a dry-loading technique with Celite.

More extensive descriptions of the methods as well as characterization data and spectra are included as Supporting Information.

## Supplementary Material



## Notes

Additional experimental details, characterization data, and spectra (PDF)
