# Supplementary material for: Chemoselective Difluorination of Tetramic Acids in Water
Source: ACS Omega. 2026 Mar 30;11(14):21836–43. doi: 10.1021/acsomega.5c12048 (PMC13084380; doi:10.1021/acsomega.5c12048)
Supplement: Supplementary file 1 [file ao5c12048_si_001.pdf]

Supporting Information

Chemoselective Difluorination of Tetramic Acids in Water

M. M. Swatscheno[a,†], R. Mahawongnan[a,†], Itamar Blau[a,†], Jacob S. Tracy[a,*]
[a]Department of Chemistry, University of West Florida, Pensacola, FL 32514, United States
[†]M.M.S., R.M. and I.B. contributed equally.
*jtracy@uwf.edu

Table of Contents

I. General Information....................S2
II. Experimental Procedures and Compound Characterization....................S3
III. SI References....................S25
IV. Copies of NMR Spectra....................S26

I. General Information

Unless otherwise indicated, all reactions were carried out in glassware sealed with rubber septa, kept under a positive pressure of nitrogen from a balloon, and stirred with a PTFE-coated magnetic stir bar. All commercial reagents were used without further purification. Dry dichloromethane (DCM) was purchased in an AcroSeal bottle over molecular sieves and used as received. Deionized water was used for all reactions and workups. Solvents were transferred via stainless steel needles and plastic syringes. Reactions were monitored via thin layer chromatography (TLC) on Supelco glass backed TLC plates (250 μm thickness, 60 Å pore diameter, F-254 indicator) and visualized via UV irradiation (254 nm) and/or aqueous potassium permanganate solution followed by heating. Solvents were removed under reduced pressure with a rotatory evaporator and compounds dried under high vacuum on a Schlenk line. Manual flash chromatography was performed unless otherwise indicated and utilized glass columns pressurized by compressed air and silica gel (SiliCycle P60, 40-63 μm, 230-400 mesh). Automated flash chromatography was only performed when indicated and utilized a Biotage Selekt Enkel with self-packed columns (silica gel, SiliCycle P60, 40-63 μm, 230-400 mesh). In all cases, the eluting solvents, their corresponding ratios, and any linear gradients are listed individually for each compound.

NMR spectra were acquired with a Bruker Ascend 400 magnet and an Avance Neo console operating at 400 MHz for ^{1}H-NMR, 101 MHz for ^{13}C-NMR, and 376 MHz for ^{19}F-NMR. Chemical shifts are reported relative to the residual solvent signal (Chloroform-d ($CDCl_3$): ^{1}H-NMR: δ = 7.26 ppm; ^{13}C-NMR: δ = 77.16 ppm or dimethylsulfoxide-d$_6$ (DMSO-d$_6$): ^{1}H-NMR δ = 2.50 ppm; ^{13}C-NMR: δ = 39.52 ppm or acetonitrile-d$_3$ (CD_3CN): ^{1}H-NMR: δ = 1.94 ppm; ^{13}C-NMR: δ = 1.32 ppm or 118.26 ppm). Multiplicities are indicated with s = singlet, d = doublet, t = triplet, q = quartet, p = pentet or quintet, m = multiplet or obscured peaks, br = broad resonance. All ^{13}C NMR spectra are proton decoupled. High-resolution mass spectra (HRMS) were acquired on an Agilent 6530 LC/Q-TOF (ESI) located in the Chemistry Department at the University of West Florida.

In the case of the tetramic acid starting materials, mixtures of keto and enol tautomers were often observed when characterized in $CDCl_3$. To enable easier characterization of these compounds, NMR spectra were most often acquired in DMSO-d$_6$, where the enol tautomer was strongly preferred. Likewise, the difluorinated tetramic acid products often showed up as mixtures of the keto and hydrate forms of the tetramic acids in common NMR solvents. The ratio of these depended upon the identity of the NMR solvent, the amount of trace water in the NMR solvent, and how long the products spent in the NMR solvent. While the 3,3-difuorinated tetramic acid products isolated following flash chromatography are predominantly in the keto form, NMR were most often acquired in DMSO-d$_6$ or CD_3CN, where the presence of water and the strong preference for formation of the hydrate of these products simplified the NMR spectra. In some cases, small amounts of water (2-3 μL) were added to these NMR solvents to ensure complete formation of the hydrate.

II. Experimental Procedures and Compound Characterization

Synthesis of Tetramic Acid Starting Materials

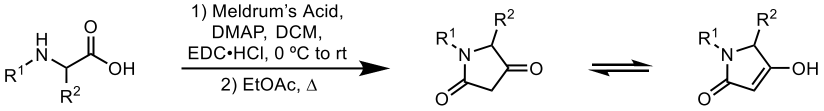

Scheme S1: General synthesis of tetramic acids.

General Procedure A:

This procedure is based upon the reported procedure of Nisato and coworkers.[1]

A flame-dried 100 mL round bottom flask equipped with a magnetic stir bar was charged with the corresponding amino acid (1.0 equiv), Meldrum's acid (2,2-dimethyl-1,3-dioxane-4,6-dione) (1.1 equiv), and DMAP (4-dimethylaminopyridine) (1.5 equiv) and placed under a nitrogen atmosphere. Dry DCM (0.17 M relative to the amino acid) was added, and the reaction was cooled to 0 ºC (ice water bath). EDC·HCl (1-(3-dimethylaminopropyl)-3-ethylcarbodiimide hydrochloride) (1.5 equiv) was added and after several minutes the ice water bath was removed, after which the reaction mixture was stirred at room temperature for 2 h. The reaction was then cooled to 0 ºC (ice bath), diluted with cold ethyl acetate (EtOAc) (100 mL per 5 mmol of amino acid) and quenched with cold brine (50 mL per 5 mmol of amino acid). The organic layer was then washed with cold 5% aqueous $KHSO_4$ (3 × 100 mL per 5 mmol of amino acid) followed by brine (50 mL per 5 mmol of amino acid). The organic layer was then dried (Na_2SO_4), filtered, and concentrated under reduced pressure to an often-yellow residue. The residue, used without further purification, was placed under a nitrogen atmosphere, diluted with EtOAc (0.05 M), and heated at reflux for 30-60 min (oil bath, 90 ºC). At that point, the reaction was cooled to room temperature, concentrated under reduced pressure, and purified via flash chromatography (SiO_2) or recrystallization.

Synthesis of Deprotected Tetramic Acid Starting Materials

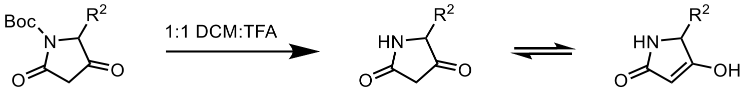

Scheme S2: General Boc-deprotection of tetramic acids.

General Procedure B:

A flame-dried two-dram screw cap vial equipped with a magnetic stir bar was charged with the starting tetramic acid and placed under a nitrogen atmosphere. Dry DCM (1 M) was added, the reaction vial cooled to 0 ºC (ice water bath), and then trifluoroacetic acid (TFA) (1 M) was added.

The reaction mixture was stirred for 1 min in the ice water bath, at which point the bath was removed, and the reaction mixture stirred for 9 additional min at room temperature. Volatiles were then removed under reduced pressure. Toluene (PhMe) (2.9 mL per mmol of starting tetramic acid) was then added and volatiles were once again removed under reduced pressure three times in a row to remove remaining traces of TFA. Isolation of the resulting product was then performed as described for each individual example.

tert-butyl 2-benzyl-3,5-dioxopyrrolidine-1-carboxylate (**6**)

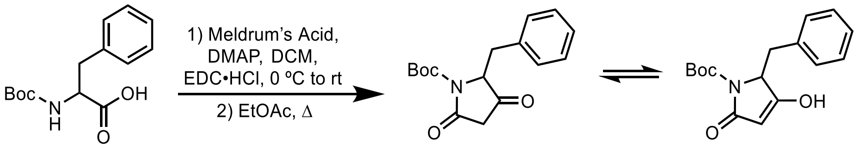

Scheme S3: Synthesis of tetramic acid **6.**

Synthesized according to general procedure A with Boc-Phe-OH (1.33g, 5.00 mmol), Meldrum's acid (0.793 g, 5.50 mmol), DMAP (0.916 g, 7.50 mmol), DCM (30 mL), EDC•HCl (1.15 g, 6.00 mmol), and EtOAc (100 mL). This substrate was heated at reflux for 30 min (oil bath, 93 ºC). The crude product was recrystallized by dissolving the crude mixture in EtOAc (70 mL) and followed by a slow diffusion of layered hexanes at 0 ºC to yield a white solid (1.020 g, 70%).

NMR characterization was performed in DMSO-d_6 which resulted in characterization of the enol tautomer. Note: this product readily incorporates trace ethyl acetate. Efforts to remove this under vacuum at room temperature up to 65 ºC were unsuccessful. Therefore, this product was characterized and used in subsequent steps with small amounts of ethyl acetate present. ^{1}H NMR (400 MHz, DMSO) δ 12.37 (s, 1H), 7.32 – 7.15 (m, 3H), 7.00 (d, J = 6.4 Hz, 1H), 4.66 (s, 1H), 4.62 (dd, J = 5.5, 2.6 Hz, 1H), 3.35 (dd, J = 13.7, 5.6 Hz, 1H), 3.07 (dd, J = 13.9, 2.7 Hz, 1H), 1.50 (s, 9H). ^{13}C NMR (101 MHz, DMSO) δ 175.5, 168.7, 149.0, 134.5, 129.5, 127.9, 126.6, 94.8, 81.0, 59.7, 34.2, 27.8. Data are consistent with that reported in the literature.[1]

5-benzylpyrrolidine-2,4-dione (**SI-1**)

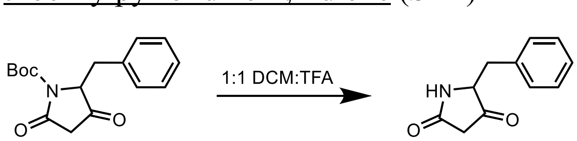

Scheme S4: Synthesis of tetramic acid **SI-1**.

Synthesized according to general procedure B with tetramic acid **6** (300. mg, 0.922 mmol), DCM (0.9 mL), and TFA (0.9 mL). After the final removal of volatiles with PhMe, the resulting solid was washed with PhMe (2 mL) and diethyl ether (Et_2O) (2 mL), filtered and washed with Et_2O to yield a uniform white powder that was used without further purification (139 mg, 80%).

Note: this product was characterized in DMSO-d_6 as 1:0.6 ratio of the keto:enol tautomers.
Enol: ^{1}H NMR (400 MHz, DMSO) δ 11.37 (s, 1H), 7.32 – 7.10 (m, 6H), 4.57 (s, 1H), 4.17 (t, J = 5.0 Hz, 1H), 2.97 (dd, J = 13.8, 4.4 Hz, 1H), 2.78 (dd, J = 13.8, 5.7 Hz, 1H).
Keto: ^{1}H NMR (400 MHz, DMSO) δ 8.43 (s, 1H), 7.32 – 7.10 (m, 5H), 4.25 (dt, J = 5.3, 2.7 Hz, 1H), 2.93 – 2.85 (m, 3H), 2.44 (dd, J = 21.8, 1.7 Hz, 1H).
Combined enol and keto: ^{13}C NMR (101 MHz, DMSO) δ 209.3, 175.0, 173.8, 170.2, 136.3, 136.0, 129.7, 129.6, 128.2, 127.8, 126.5, 126.2, 94.6, 64.1, 57.4, 40.8, 37.2, 36.8. HRMS (ESI) m/z calculated for $C_{11}H_{11}NO_2$ (H^+): 190.0863, found: 190.0864.

tert-butyl 2-methyl-3,5-dioxopyrrolidine-1-carboxylate **(SI-2)**

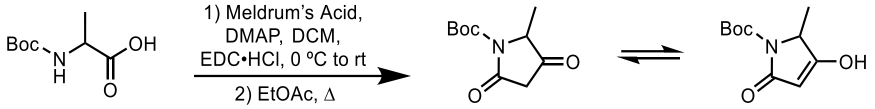

Scheme S5: Synthesis of tetramic acid **SI-2**.

Synthesized according to general procedure A with Boc-Ala-OH (0.946 g, 5.00 mmol), Meldrum's acid (0.793 g, 5.50 mmol), DMAP (0.916 g, 7.50 mmol), DCM (30 mL), EDC•HCl (1.15 g, 6.00 mmol), and EtOAc (100 mL). This substrate was heated at reflux for 30 min (oil bath, 104 ºC). The crude product was recrystallized from hot EtOAc (43 mL) to yield a yellow solid (302 mg, 28%).

NMR characterization was performed in $CDCl_3$ which resulted in a 2:1 ratio of the ketone to enol tautomer. The major ketone tautomer was characterized: ^{1}H NMR (400 MHz, $CDCl_3$) δ 4.41 (q, J = 6.9 Hz, 1H), 3.31 – 3.14 (m, 1H), 1.56 (s, 9H), 1.51 (d, J = 7.4 Hz, 3H). ^{13}C NMR (101 MHz, $CDCl_3$) δ 204.3, 167.5, 149.1, 84.4, 63.7, 42.2, 28.2, 17.5. Data are consistent with that reported in the literature.[2]

2-((*tert*-butoxycarbonyl)amino)pent-4-ynoic acid (**SI-3A**)

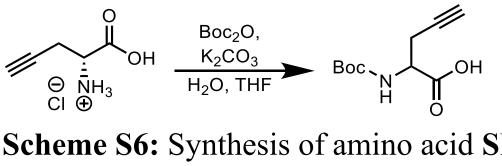

Scheme S6: Synthesis of amino acid **SI-3A**.

Prepared following the modified procedure of Granja and coworkers.[3]

A flame-dried 25 mL round bottom flask equipped with a magnetic stir bar was charged with (*R*)-2-aminopent-4-ynoic acid hydrochloride (1.10 g, 7.35 mmol) and K_2CO_3 (4.06 g, 29.4 mmol) and

then placed under a nitrogen atmosphere. Water (7.5 mL) and THF (2.5 mL) were added followed by di-*tert*-butyl di-carbonate (1.94 mL, 8.45 mmol) neat via syringe. The resulting biphasic reaction mixture was stirred strongly overnight, at which point it was concentrated under reduced pressure. The resulting crude material was partitioned between 10 mL of Et_2O and 10 mL water. The organic layer was discarded, and the aqueous layer was washed with another 10 mL of Et_2O. The organic layer was then acidified to pH 3 using malic acid and $KHSO_4$. The acidified aqueous layer was extracted with DCM (3 × 30 mL). The combined organic layers were then washed with water (3 × 30 mL), dried (Na_2SO_4), filtered, and concentrated under reduced pressure. The crude product was purified via flash chromatography (SiO_2, DCM with 1% AcOH and 1% MeOH) to yield a white powder (921 mg, 44%).

^{1}H NMR (400 MHz, $CDCl_3$) δ 5.32 (d, J = 8.4 Hz, 1H), 4.64 – 4.43 (m, 1H), 2.86 – 2.66 (m, 2H), 2.09 (t, J = 2.6 Hz, 1H), 1.46 (s, 9H). Data are consistent with those reported in the literature.[3]

<u>*tert*-butyl 3,5-dioxo-2-(prop-2-yn-1-yl)pyrrolidine-1-carboxylate</u> (**SI-3B**)

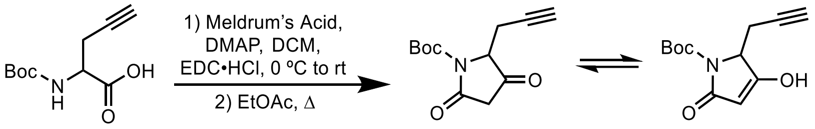

Scheme S7: Synthesis of tetramic acid **SI-3B**.

Synthesized according to general procedure A with **SI-3A** (0.921 g, 3.88 mmol), Meldrum's acid (0.615 g, 4.27 mmol), DMAP (0.711 g, 5.82 mmol), DCM (23 mL), EDC•HCl (0.893 g, 4.27 mmol), and EtOAc (100 mL). This substrate was heated at reflux for 60 min (oil bath, 92 ºC). The crude product was recrystallized from a 1:1 mixture of hexanes:EtOAc (30 mL) that was layered with hexane (60 mL) upon cooling and the two layers were allowed to slowly mix via diffusion at 0 ºC to yield a yellow solid (258 mg, 25%).

NMR characterization was performed in DMSO-d_6 which resulted in characterization of the enol tautomer. ^{1}H NMR (400 MHz, DMSO) δ 12.35 (s, 1H), 4.93 (s, 1H), 4.46 (dd, J = 5.1, 2.4 Hz, 1H), 3.01 (ddd, J = 17.1, 5.2, 2.8 Hz, 1H), 2.84 (t, J = 2.4 Hz, 1H), 2.67 (dt, J = 17.1, 2.6 Hz, 1H), 1.45 (s, 9H). ^{13}C NMR (101 MHz, DMSO) δ 174.9, 169.0, 148.8, 94.8, 81.1, 77.5, 74.0, 57.4, 27.8, 19.7. HRMS (ESI) m/z calculated for $C_{12}H_{15}NO_4$ (-H^+): 236.0928, found: 236.0927.

<u>*tert*-butyl 2-(4-((*tert*-butoxycarbonyl)amino)butyl)-3,5-dioxopyrrolidine-1-carboxylate</u> (**SI-4**)

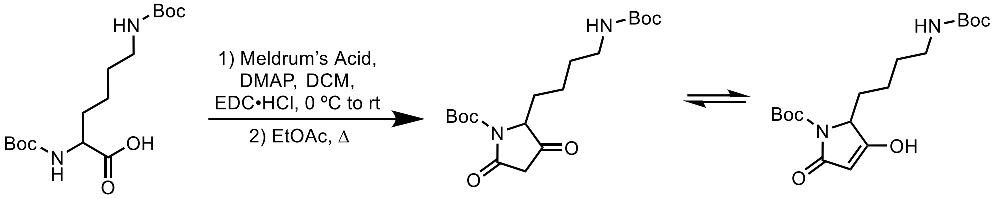

Scheme S8: Synthesis of tetramic acid **SI-4**.

Synthesized according to general procedure A with Boc-Lys(Boc)-OH (1.73 g, 5.00 mmol), Meldrum's acid (0.793 g, 5.50 mmol), DMAP (0.916 g, 7.50 mmol), DCM (30 mL), EDC•HCl (1.15 g, 6.00 mmol), and EtOAc (100 mL). This substrate was heated at reflux for 30 min (oil bath, 96-104 ºC). The crude product was recrystallized by dissolving the crude mixture in EtOAc (10 mL) and followed by a slow diffusion of layered hexanes at 0 ºC to yield a white solid (1.131 g, 61%).

NMR characterization was performed in DMSO-d_6 which resulted in characterization of the enol tautomer. ^{1}H NMR (400 MHz, DMSO) δ 12.25 (s, 1H), 6.76 (t, J = 5.8 Hz, 1H), 4.86 (s, 1H), 4.38 (dd, J = 5.6, 2.7 Hz, 1H), 2.84 (q, J = 6.5 Hz, 1H), 2.03 – 1.90 (m, 1H), 1.81 – 1.69 (m, 1H), 1.44 (s, 9H), 1.35 (s, 9H), 1.34 – 1.26 (m, 2H), 1.12 – 0.96 (m, 2H). ^{13}C NMR (101 MHz, DMSO) δ 176.7, 169.2, 155.5, 148.8, 94.1, 80.8, 77.3, 59.2, 39.5, 29.4, 28.4, 28.2, 27.8, 19.1. HRMS (ESI) m/z calculated for $C_{18}H_{30}N_2O_6$ (H^+): 371.2177, found: 371.2184. Data are consistent with those reported in the literature.[4]

<u>*tert*-butyl 3,5-dioxo-2-phenylpyrrolidine-1-carboxylate</u> (**SI-5**)

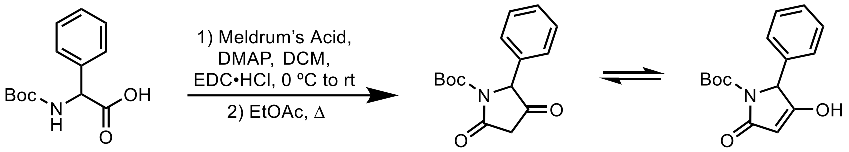

Scheme S9: Synthesis of tetramic acid **SI-5**.

Synthesized according to general procedure A with 2-((*tert*-Butoxy carbonyl)amino)-2-phenylacetic acid (1.26 g, 5.00 mmol), Meldrum's acid (0.793 g, 5.50 mmol), DMAP (0.916 g, 7.50 mmol), DCM (30 mL), EDC•HCl (1.15 g, 6.00 mmol), and EtOAc (100 mL). This substrate was heated at reflux for 30 min (oil bath, 97-101 ºC). The crude product was recrystallized by dissolving the crude mixture in EtOAc (35 mL) and followed by a slow diffusion of layered hexanes (70 mL) at 0 ºC to yield a yellow foam (543 mg, 39%).

NMR characterization was performed in DMSO-d_6 which resulted in characterization of the enol tautomer. ^{1}H NMR (400 MHz, DMSO) δ 12.34 (s, 1H), 7.45 – 7.28 (m, 3H), 7.25 – 7.18 (m, 2H), 5.33 (s, 1H), 4.94 (s, 1H), 1.16 (s, 9H). ^{13}C NMR (101 MHz, DMSO) δ 176.6, 169.6, 148.1, 136.9,

128.4, 128.1, 126.8, 93.1, 80.7, 63.7, 27.4. HRMS (ESI) m/z calculated for $C_{15}H_{17}NO_4$ (H^+): 276.1230, found: 276.1235.

tert-butyl 2-(4-nitrobenzyl)-3,5-dioxopyrrolidine-1-carboxylate (**SI-6**)

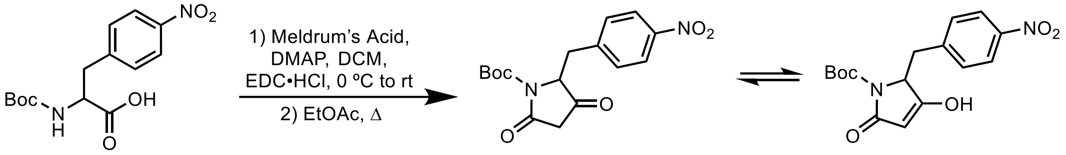

Scheme S10: Synthesis of tetramic acid **SI-6**.

Synthesized according to general procedure A with Boc-Phe (4-NO_2)-OH (1.55 g, 5.00 mmol), Meldrum's acid (0.793 g, 5.50 mmol), DMAP (0.916 g, 7.50 mmol), DCM (30 mL), EDC•HCl (1.15 g, 6.00 mmol), and EtOAc (100 mL). This substrate was heated at reflux for 30 min (oil bath, 94-102 ºC). The crude product was recrystallized by dissolving the crude mixture in EtOAc (15 mL) and followed by a slow diffusion of layered hexanes at 0 ºC to yield a yellow solid (699 mg, 42%).

NMR characterization was performed in DMSO-d_6 which resulted in characterization of the enol tautomer as the major species although some keto tautomer can be seen in the NMR.^{1}H NMR (400 MHz, DMSO) δ 12.55 (s, 1H), 8.15 (d, J = 8.7 Hz, 2H), 7.26 (d, J = 8.7 Hz, 2H), 4.73 (dd, J = 5.7, 2.8 Hz, 1H), 4.71 (s, 1H), 3.47 (dd, J = 13.7, 5.6 Hz, 1H), 3.22 (dd, J = 13.7, 2.8 Hz, 1H), 1.51 (s, 9H). ^{13}C NMR (101 MHz, DMSO) δ 175.2, 168.5, 149.0, 146.5, 143.0, 130.8, 123.1, 94.9, 81.4, 59.4, 34.3, 27.8. HRMS (ESI) m/z calculated for $C_{15}H_{17}NO_4$ (H^+): 335.1238, found: 335.1242.

tert-butyl 2-isopropyl-3,5-dioxopyrrolidine-1-carboxylate (**SI-7**)

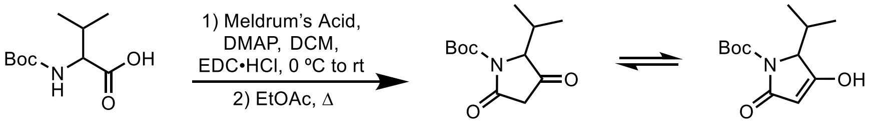

Scheme S11: Synthesis of tetramic acid **SI-7**.

Synthesized according to general procedure A with Boc-Val-OH (1.09 g, 5.00 mmol), Meldrum's acid (0.793 g, 5.50 mmol), DMAP (0.916 g, 7.50 mmol), DCM (30 mL), EDC•HCl (1.15 g, 6.00 mmol), and EtOAc (100 mL). This substrate was heated at reflux for 60 min (oil bath, 93-97 ºC). The crude product was purified via flash chromatography (SiO_2, 1:1 hexanes:EtOAc then 100% EtOAc) to yield an off-white foam (584 mg, 48%).

NMR characterization was performed in DMSO-d_6 which resulted in characterization of the enol tautomer. ^{1}H NMR (400 MHz, DMSO) δ 12.23 (s, 1H), 4.86 (s, 1H), 4.26 (d, J = 2.6 Hz, 1H), 2.34

(heptd, $J = 7.1, 2.7$ Hz, 1H), 1.44 (s, 9H), 1.03 (d, $J = 7.2$ Hz, 3H), 0.76 (d, $J = 7.0$ Hz, 3H). ^{13}C NMR (101 MHz, DMSO) δ 177.0, 169.2, 149.1, 94.5, 80.9, 63.8, 28.8, 27.7, 18.1, 15.5. Data are consistent with those reported in the literature.[1]

tert-butyl 5,7-dioxo-4-azaspiro[2.4]heptane-4-carboxylate (**23**)

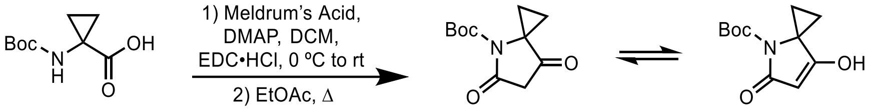

Scheme S12: Synthesis of tetramic acid **23**.

Synthesized according to general procedure A with 1-(Boc-amino) cyclopropane carboxylic acid (1.01 g, 5.00 mmol), Meldrum's acid (0.793 g, 5.50 mmol), DMAP (0.916 g, 7.50 mmol), DCM (30 mL), EDC•HCl (1.15 g, 6.00 mmol), and EtOAc (100 mL). This substrate was heated at reflux for 30 min (oil bath, 94-100 ºC). The crude product was recrystallized by dissolving the crude mixture in EtOAc followed by a slow diffusion of layered hexanes at 0 ºC to yield a yellow foam (393 mg, 35%).

NMR characterization performed in $CDCl_3$ which resulted in characterization of the keto tautomer. ^{1}H NMR (400 MHz, $CDCl_3$) δ 3.26 (s, 2H), 2.02 (q, $J = 4.5$ Hz, 2H), 1.53 (s, 9H), 1.35 (q, $J = 4.4$ Hz, 2H). ^{13}C NMR (101 MHz, $CDCl_3$) δ 203.8, 167.9, 148.7, 84.6, 53.1, 42.2, 28.1, 15.2. HRMS (ESI) m/z calculated for $C_{11}H_{15}NO_4$ (H^+): 226.1074, found: 226.1080.

tert-butyl 2,4-dioxopyrrolidine-1-carboxylate (**SI-8**)

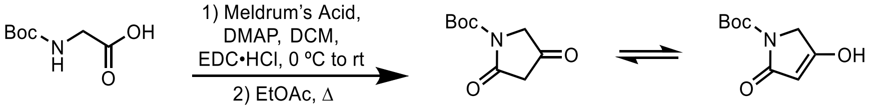

Scheme S13: Synthesis of tetramic acid **SI-8**.

Synthesized according to general procedure A with Boc-Gly-OH (0.736 g, 5.00 mmol), Meldrum's acid (0.793 g, 5.50 mmol), DMAP (0.916 g, 7.50 mmol), DCM (30 mL), EDC•HCl (1.15 g, 6.00 mmol), and EtOAc (100 mL). This substrate was heated at reflux for 46 min (oil bath, 96-101 ºC). The crude product was recrystallized by dissolving the crude mixture in EtOAc (50 mL) followed by a slow diffusion of layered hexanes at 0 ºC to yield a white solid (471 mg, 53%).

NMR characterization was performed in DMSO-d_6 which resulted in characterization of the enol tautomer. ^{1}H NMR (400 MHz, DMSO) δ 12.16 (s, 1H), 4.88 (s, 1H), 4.14 (s, 2H), 1.44 (s, 9H). ^{13}C NMR (101 MHz, DMSO) δ 174.4, 169.2, 148.9, 94.3, 80.8, 49.4, 27.8. HRMS (ESI) m/z calculated for $C_9H_{13}NO_4$ (H^+): 200.0917, found: 200.0923.

benzyl 2-benzyl-3,5-dioxopyrrolidine-1-carboxylate (**SI-9**)

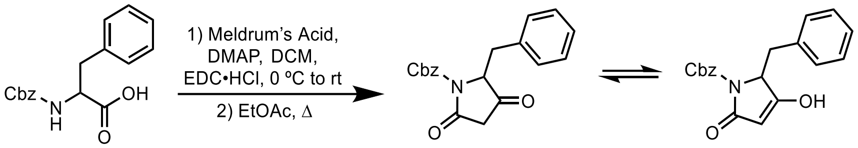

Scheme S14: Synthesis of tetramic acid **SI-9**.

Synthesized according to general procedure A with Cbz-Phe-OH (1.50 g, 5.00 mmol), Meldrum's acid (0.793 g, 5.50 mmol), DMAP (0.916 g, 7.50 mmol), DCM (30 mL), EDC•HCl (1.15 g, 6.00 mmol), and EtOAc (100 mL). This substrate was heated at reflux for 30 min (oil bath, 88-92 ºC). The crude product was purified via flash chromatography (SiO_2, 1:1 hexanes:EtOAc then 1:4 hexanes:EtOAc then 100% EtOAc) to yield a white solid (723 mg, 45%).

NMR characterization was performed in DMSO-d_6 which resulted in characterization of the enol tautomer. ^{1}H NMR (400 MHz, DMSO) δ 12.52 (s, 1H), 7.47 (d, J = 7.0 Hz, 2H), 7.42 (t, J = 7.4 Hz, 2H), 7.38 – 7.31 (m, 1H), 7.21 – 7.07 (m, 3H), 6.89 (dd, J = 6.5, 3.0 Hz, 2H), 5.29 (s, 2H), 4.75 – 4.71 (m, 1H), 4.70 (s, 1H), 3.33 (dd, J = 13.9, 5.5 Hz, 1H), 3.06 (dd, J = 13.9, 2.6 Hz, 1H). ^{13}C NMR (101 MHz, DMSO) δ 176.1, 168.5, 150.4, 136.1, 134.3, 129.5, 128.4, 128.1, 127.9, 127.9, 126.7, 94.7, 66.6, 59.8, 33.9. HRMS (ESI) m/z calculated for $C_{19}H_{17}NO_4$ (H^+): 324.1230, found: 324.1240.

(*S*)-2-((*S*)-*sec*-butyl)-3,5-dioxopyrrolidine-1-carboxylate (**SI-10**)

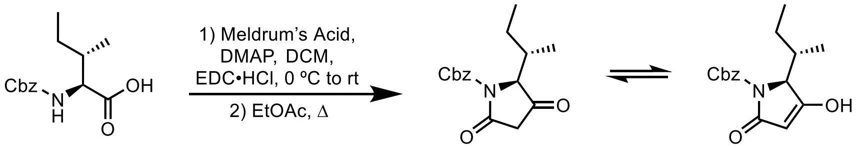

Scheme S15: Synthesis of tetramic acid **SI-10**.

Synthesized according to general procedure A with Cbz-L-Ile-OH (1.33 g, 5.00 mmol), Meldrum's acid (0.793 g, 5.50 mmol), DMAP (0.916 g, 7.50 mmol), DCM (30 mL), EDC•HCl (1.15 g, 6.00 mmol), and EtOAc (100 mL). This substrate was heated at reflux for 30 min (oil bath, 91-104 ºC). The crude product was purified via flash chromatography (SiO_2, 1:1 hexanes:EtOAc then 100% EtOAc then 10% methanol (MeOH) in EtOAc) to yield a yellow foam (514 mg, 36%).

NMR characterization was performed in DMSO-d_6 which resulted in characterization of the enol tautomer. ^{1}H NMR (400 MHz, DMSO) δ 12.37 (s, 1H), 7.50 – 7.19 (m, 5H), 5.24 (d, J = 12.7 Hz, 1H), 5.16 (d, J = 12.7 Hz, 1H), 4.90 (s, 1H), 4.44 (d, J = 2.6 Hz, 1H), 2.13 (tq, J = 9.4, 6.8 Hz, 1H), 1.56 (dp, J = 14.4, 7.3 Hz, 1H), 1.39 (dp, 1H), 0.87 (t, J = 7.4 Hz, 3H), 0.69 (d, J = 6.9 Hz, 3H). ^{13}C NMR (101 MHz, DMSO) δ 177.8, 169.1, 150.3, 136.0, 128.3, 127.9, 127.6, 94.4, 66.6,

62.6, 35.2, 25.4, 12.6, 12.0. HRMS (ESI) m/z calculated for $C_{16}H_{19}NO_4$ (H^+): 290.1387, found: 290.1397.

allyl 2,4-dioxopyrrolidine-1-carboxylate (**SI-11**)

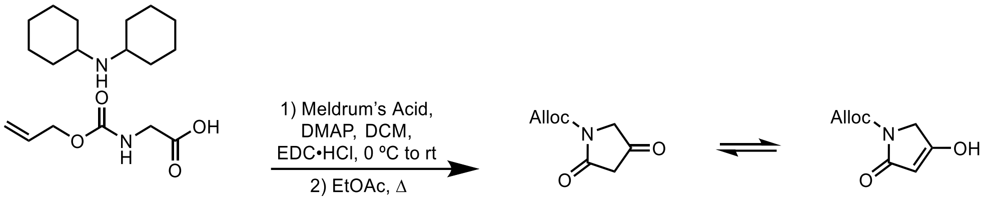

Scheme S16: Synthesis of tetramic acid **SI-11**.

Synthesized according to general procedure A with Alloc-Gly-OH•DCHA (1.70 g, 5.00 mmol), Meldrum's acid (0.793 g, 5.50 mmol), DMAP (0.916 g, 7.50 mmol), DCM (30 mL), EDC•HCl (1.15 g, 6.00 mmol), and EtOAc (100 mL). This substrate was heated at reflux for 60 min (oil bath, 88 ºC). The crude product was recrystallized from a hot 1:3 mixture of EtOAc:Hexanes to yield a white solid (169 mg, 18%).

NMR characterization was performed in DMSO-d_6 which resulted in characterization of the enol tautomer. ^{1}H NMR (400 MHz, DMSO) δ 12.30 (s, 1H), 5.94 (ddt, J = 17.3, 10.4, 5.1 Hz, 1H), 5.38 (dq, J = 17.3, 1.8 Hz, 1H), 5.22 (dq, J = 10.5, 1.6 Hz, 1H), 4.93 (s, 1H), 4.64 (dt, J = 5.2, 1.6 Hz, 2H), 4.22 (s, 2H). ^{13}C NMR (101 MHz, DMSO) δ 276.6, 270.7, 251.5, 234.1, 219.1, 195.7, 167.1, 150.8, 132.5, 117.5, 94.1, 65.5, 49.3. HRMS (ESI) m/z calculated for $C_8H_9NO_4$ (H^+): 184.0604, found: 184.0611.

1-acetyl-5-benzylpyrrolidine-2,4-dione (**SI-12**)

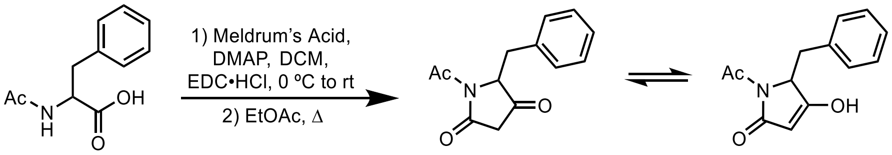

Scheme S17: Synthesis of tetramic acid **SI-12**.

Synthesized according to general procedure A with Ac-Phe-OH (0.870 g, 4.20 mmol), Meldrum's acid (0.793 g, 5.50 mmol), DMAP (0.916 g, 7.50 mmol), DCM (30 mL), EDC•HCl (1.15 g, 6.00 mmol), and EtOAc (100 mL). This substrate was heated at reflux for 60 min (oil bath, 85-87 ºC). The crude product was recrystallized by dissolving the crude mixture in EtOAc (15 mL) followed by a slow diffusion of layered hexanes (135 mL) at 0 ºC to yield a white solid (474 mg, 49%).

NMR characterization was performed in DMSO-d6 which resulted in characterization of the enol tautomer. ^{1}H NMR (400 MHz, DMSO) δ 12.60 (s, 1H), 7.27 – 7.14 (m, 3H), 6.98 – 6.90 (m, 2H), 4.74 (dd, J = 5.4, 2.6 Hz, 1H), 4.72 (s, 1H), 3.41 (dd, J = 13.7, 5.4 Hz, 1H), 3.04 (dd, J = 13.7, 2.7 Hz, 1H), 2.33 (s, 3H). ^{13}C NMR (101 MHz, DMSO) δ 177.1, 170.3, 168.7, 134.5, 129.4, 127.9, 126.7, 94.5, 59.0, 33.4, 24.7. HRMS (ESI) m/z calculated for $C_{13}H_{13}NO_3$ (H^+): 232.0968, found: 232.0974.

tert-butyl 2-isobutyl-3,5-dioxopyrrolidine-1-carboxylate (**SI-13**)

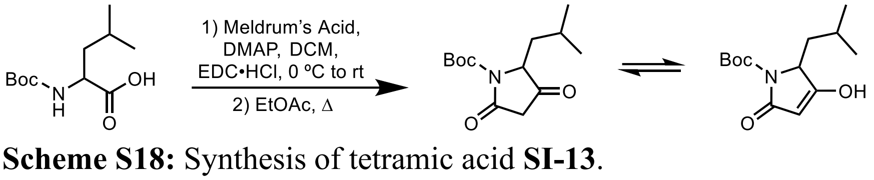

Scheme S18: Synthesis of tetramic acid **SI-13**.

Synthesized according to general procedure A with Boc-Leu-OH (1.16 g, 5.00 mmol), Meldrum's acid (0.793 g, 5.50 mmol), DMAP (0.916 g, 7.50 mmol), DCM (30 mL), EDC•HCl (1.15 g, 6.00 mmol), and EtOAc (100 mL). This substrate was heated at reflux for 30 min (oil bath, 91-104 ºC). The crude product was purified via automated flash chromatography (SiO_2, 1:1 hexanes:EtOAc) to yield a yellow foam that was not analytically pure but carried forward to the next step (494 mg, 46%).

NMR characterization was performed in DMSO-d6 which resulted in characterization of the enol tautomer. ^{1}H NMR (400 MHz, DMSO) δ 12.31 (s, 1H), 4.85 (s, 1H), 4.38 (dd, J = 6.5, 2.9 Hz, 1H), 1.83 – 1.63 (m, 3H), 1.45 (d, J = 2.9 Hz, 9H), 0.86 (obscured doublets J = 5.3 Hz, 6H total). ^{13}C NMR (101 MHz, DMSO) δ 177.9, 169.2, 148.8, 93.7, 80.9, 58.7, 38.5, 27.8, 23.7, 23.2, 23.1. Data are consistent with those reported in the literature.[1]

5-isobutylpyrrolidine-2,4-dione (**SI-14**)

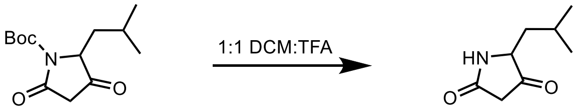

Scheme S19: Synthesis of tetramic acid **SI-14**.

Synthesized according to general procedure B with the impure tetramic acid **SI-13** (200. mg, 0.783 mmol), DCM (1 mL), and TFA (1 mL). After the final removal of volatiles with PhMe, the resulting solid was dissolved in PhMe (0.5 mL). To this was added Et_2O (1 mL) then hexanes (4 mL) to precipitate the product which was then filtered off to yield an off-white powder that was used without further purification (60.4 mg, 50%).

NMR characterization was performed in $CDCl_3$ which resulted in characterization of the keto tautomer. ^{1}H NMR (400 MHz, $CDCl_3$) δ 7.70 (s, 1H), 4.02 (dd, J = 9.6, 4.3 Hz, 1H), 3.02 (d, J = 1.1 Hz, 2H), 1.83 – 1.68 (m, 1H), 1.65 (td, J = 9.1, 4.5 Hz, 1H), 1.49 (ddd, J = 13.8, 9.6, 5.4 Hz, 1H), 0.95 (t, J = 6.2 Hz, 6H). ^{13}C NMR (101 MHz, $CDCl_3$) δ 207.9, 171.7, 63.0, 41.4, 40.6, 25.1, 23.3, 21.6. HRMS (ESI) m/z calculated for $C_8H_{13}NO_2$ (H^+): 156.1019, found: 156.1021.

<u>*tert*-butyl 2-((1-formyl-1*H*-indol-3-yl)methyl)-3,5-dioxopyrrolidine-1-carboxylate</u> **(SI-15)**

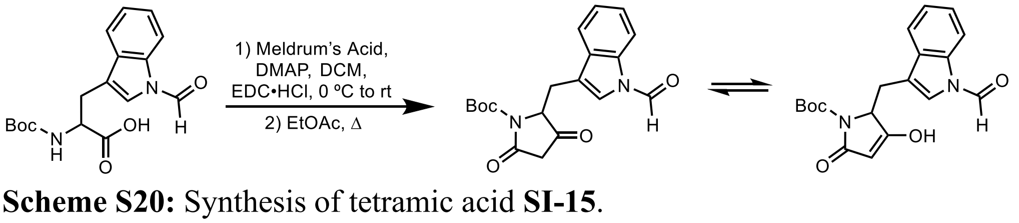

Scheme S20: Synthesis of tetramic acid **SI-15**.

Synthesized according to general procedure A with Boc-Trp(for)-OH (1.00 g, 3.00 mmol), Meldrum's acid (0.476 g, 3.30 mmol), DMAP (0.550 g, 4.50 mmol), DCM (18 mL), EDC•HCl (0.69 g, 3.6 mmol), and EtOAc (60 mL). This substrate was heated at reflux for 60 min (oil bath, 92-96 ºC). This material was directly carried onto the next step without purification.

<u>3-((3,5-dioxopyrrolidin-2-yl)methyl)-1*H*-indole-1-carbaldehyde</u> (**SI-16**)

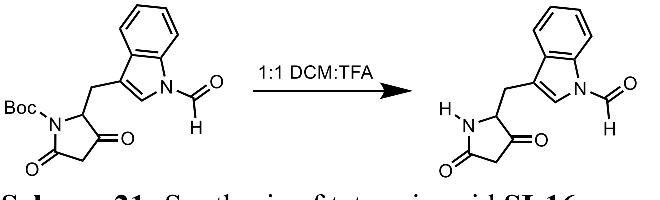

Scheme 21: Synthesis of tetramic acid **SI-16**.

Synthesized according to general procedure B with tetramic acid **SI-15** (273 mg, 0.765 mmol), DCM (0.7 mL), and TFA (0.7 mL). After the final removal of volatiles with PhMe, the resulting solid was dissolved in EtOAc (1 mL) and PhMe was slowly added until solid began to precipitate. At that point, Et_2O was added to complete the precipitation of the desired product as a white powder (138 mg, 18% over two steps).

NMR characterization was performed in DMSO-d_6 which resulted in a 6:4 ratio of keto:enol tautomers that was further complicated by the presence of rotameric species as a result of the formamide. As a result, the ^{1}H and ^{13}C data are presented together and are not assigned to a specific tautomer: ^{1}H NMR (400 MHz, DMSO) δ 11.45 (s, 0.4H), 10.13 – 6.99 (series of tautomeric and rotameric peaks, 7H total), 4.64 (s, 0.4H), 4.49 – 4.12 (m, 1H), 3.20 – 2.64 (m, 3.2H). ^{13}C NMR (101 MHz, DMSO) δ 209.2, 175.3, 173.9, 170.4, 161.2, 157.9, 133.5, 131.0, 125.3, 124.7, 123.9,

123.5, 119.5, 117.5, 116.9, 115.1, 110.7, 94.4, 62.9, 56.3, 40.8, 26.7. HRMS (ESI) m/z calculated for $C_{14}H_{12}N_2O_3$ (H^+): 257.0921, found: 257.0928.

Synthesis of 3,3-Difluorinated Tetramic Acids

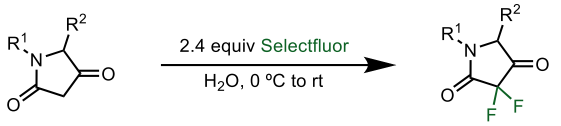

Scheme S22: Generic difluorination of tetramic acids.

General Procedure C:

A 1-dram screw cap vial equipped with a magnetic stir bar was charged with the starting tetramic acid (1.0 equiv) and Selectfluor (2.4 equiv) then placed under a nitrogen atmosphere. The vial was cooled to 0 ºC (ice water bath) and H_2O (0.25 M) was then added. After 15 minutes, the ice water bath was removed and the reaction was allowed to stir at room temperature with strong stirring. The indicated times below are total stirring times and include both within the ice water bath and at room temperature. At that point, the reaction was diluted with EtOAc (30 mL per 0.25 mmol of starting tetramic acid) and water (20 mL per 0.25 mmol of starting tetramic acid). The layers were separated and the aqueous layer was extracted with EtOAc (8 × 20 mL per 0.25 mmol of starting tetramic acid). The combined organic layers were then washed with brine (50 mL per 0.25 mmol of starting tetramic acid), dried (Na_2SO_4), filtered, and concentrated under reduced pressure. The resulting residues were purified via manual flash chromatography (SiO_2) and loaded onto the column utilizing a dry-loading technique with Celite.

tert-butyl 5-benzyl-3,3-difluoro-2,4-dioxopyrrolidine-1-carboxylate (**7**)

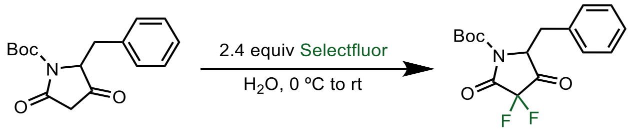

Scheme S23: Synthesis of difluorinated tetramic acid **7**.

0.25 mmol scale reaction:

Synthesized according to general procedure C with **6** (72.3 mg, 0.250 mmol), Selectfluor (213 mg, 0.600 mmol), and H_2O (1.0 mL) for 22 h. The crude product was purified via flash chromatography (SiO_2, 4:1 hexanes:EtOAc then 2:1 hexanes:EtOAc) to yield a white solid (72.9 mg, 82%).

0.25 mmol scale reaction under air:

Synthesized according to general procedure C except under an air atmosphere with **6** (72.3 mg, 0.250 mmol), Selectfluor (213 mg, 0.600 mmol), and H_2O (1.0 mL) for 22 h. The crude product was purified via flash chromatography (SiO_2, 2.5:1 hexanes:EtOAc) to yield a white solid (61.2 mg, 75%).

2 mmol scale reaction:

A 25 mL round-bottom flask equipped with a magnetic stir bar was charged with **6** (579 mg, 2.00 mmol) and Selectfluor (1.70 g, 4.80 mmol) then placed under a nitrogen atmosphere. The flask was placed in an ice water bath and H_2O (8.0 mL) was then added. After 15 minutes, the water bath was removed, and the reaction was allowed to stir at room temperature with strong stirring for 23 h. At that point, the reaction was diluted with EtOAc (60 mL) and water (30 mL). The layers were separated, and the aqueous layer was extracted with EtOAc (8 × 40 mL). The combined organic layers were then washed with brine (60 mL), dried (Na_2SO_4), filtered, and concentrated under reduced pressure. The crude product was purified via flash chromatography (SiO_2, 2.5:1 hexanes:EtOAc) to yield a white solid (584 mg, 90%).

5.2 mmol scale reaction (gram-scale):

A 100 mL round-bottom flask equipped with a magnetic stir bar was charged with **6** (1.50 g, 5.18 mmol) and Selectfluor (3.92 g, 11.1 mmol) then placed under a nitrogen atmosphere. The flask was placed in an ice water bath and H_2O (18.4 mL) was then added. After 15 minutes, the water bath was removed, and the reaction was allowed to stir at room temperature with strong stirring for 24 h. At that point, the reaction was diluted with EtOAc (100 mL) and water (60 mL). The layers were separated, and the aqueous layer was extracted with EtOAc (8 × 80 mL). The combined organic layers were then washed with brine (120 mL), dried (Na_2SO_4), filtered, and concentrated under reduced pressure. The crude product was purified via flash chromatography (SiO_2, 2.5:1 hexanes:EtOAc) to yield a white solid (1.48 g, 88%).

NMR characterization was performed in DMSO-d_6 to favor formation of the hydrate from trace water in the NMR solvent and allow the product to be readily characterized as one major species (**7·H2O**). The NMR are therefore the hydrate of **7**. See section I. General Information for more details. R_f (hexanes: EtOAc 4:1) = 0.14. ^{1}H NMR (400 MHz, DMSO) δ 7.62 (s, 1H), 7.43 (s, 1H), 7.29 (t, J = 7.5 Hz, 2H), 7.25 – 7.18 (m, 3H), 4.34 (dd, J = 6.1, 6.1 Hz, 1H), 3.15 (dd, J = 14.3, 5.8 Hz, 1H), 2.65 (dd, J = 14.3, 7.4 Hz, 1H), 1.26 (s, 9H). ^{13}C NMR (101 MHz, DMSO) δ 161.5 (dd, $J_{C\text{-}F}$ = 32.6, 28.1 Hz), 148.4, 137.8, 129.4, 128.3, 126.3, 111.8 (dd, $J_{C\text{-}F}$ = 267.9, 252.2 Hz), 92.1 (dd, $J_{C\text{-}F}$ = 21.4, 17.8 Hz), 83.7, 65.5, 36.8. ^{19}F NMR (376 MHz, DMSO) δ -111.6 (d, $J_{F\text{-}F}$ = 262.0 Hz, 1F), -133.7 (dd, $J_{F\text{-}F}$ = 262.1, $J_{H\text{-}F}$ = 5.3 Hz, 1F). HRMS (ESI) m/z calculated for $C_{16}H_{17}F_2NO_4$ ($-H^+$): 324.1053, found: 324.1050.

tert-butyl 3,3-difluoro-5-methyl-2,4-dioxopyrrolidine-1-carboxylate (**8**)

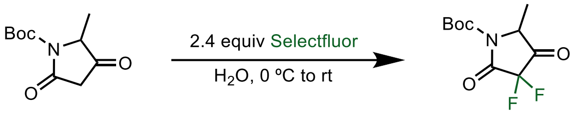

Scheme S24: Synthesis of difluorinated tetramic acid **8.**

Synthesized according to general procedure C with **SI-2** (53.3 mg, 0.250 mmol), Selectfluor (213 mg, 0.600 mmol), and H_2O (1.0 mL) for 1 h 20 min. The crude product was purified via flash chromatography (SiO_2, 1.6:1 hexanes:EtOAc) to yield a white solid (48.5 mg, 77%).

NMR characterization was performed in CD_3CN to favor formation of the hydrate from trace water in the NMR solvent and allow the product to be readily characterized as one major species (**8·H2O**). The NMR are therefore the hydrate of **8**. See section I. General Information for more details. R_f (hexanes: EtOAc 1.6:1) = 0.27. ^{1}H NMR (400 MHz, CD_3CN) δ 5.12 (s, 1H), 5.04 (s, 1H), 4.09 (dqd, J = 7.7, 6.8, 5.4 Hz, 1H), 1.51 (s, 9H), 1.30 (dd, J = 6.7, 1.9 Hz, 3H). ^{13}C NMR (101 MHz, CD_3CN) δ 162.2 (dd, $J_{C\text{-}F}$ = 32.3, 28.4 Hz), 149.8, 112.5 (dd, J = 264.4, 254.9 Hz), 93.1 (dd, $J_{C\text{-}F}$ = 20.8, 18.8 Hz), 85.3, 61.9 (d, $J_{C\text{-}F}$ = 2.4 Hz), 28.0, 15.4 (d, $J_{C\text{-}F}$ = 2.3 Hz). ^{19}F NMR (376 MHz, CD_3CN) δ -115.8 (d, $J_{F\text{-}F}$ = 265.5 Hz, 1F), -134.5 (dd, $J_{F\text{-}F}$ = 265.2, $J_{H\text{-}F}$ = 4.9 Hz, 1F). HRMS (ESI) m/z calculated for $C_{10}H_{13}F_2NO_4$ (-H^+): 248.0740, found: 248.0736.

tert-butyl 3,3-difluoro-2,4-dioxo-5-(prop-2-yn-1-yl)pyrrolidine-1-carboxylate (**9**)

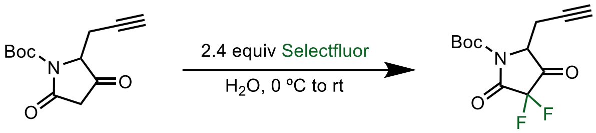

Scheme S25: Synthesis of difluorinated tetramic acid **9**.

Synthesized according to general procedure C with **SI-3B** (59.3 mg, 0.250 mmol), Selectfluor (213 mg, 0.600 mmol), and H_2O (1.0 mL) for 30 min. The crude product was purified via flash chromatography (SiO_2, 1.8:1 hexanes:EtOAc) to yield a white solid (59.4 mg, 87%).

NMR characterization was performed in DMSO-d_6 to favor formation of the hydrate from trace water in the NMR solvent and allow the product to be readily characterized as one major species (**9·H2O**). The NMR are therefore the hydrate of **9**. See section I. General Information for more details. R_f (hexanes: EtOAc 2:1) = 0.20. ^{1}H NMR (400 MHz, DMSO) δ 7.66 (s, 1H), 7.48 (d, J = 1.5 Hz, 1H), 4.18 (dt, J = 6.7, 4.9 Hz, 1H), 2.88 (t, J = 2.7 Hz, 1H), 2.68 (ddd, J = 17.5, 7.0, 2.7 Hz, 1H), 2.51 (ddd, J = 17.5, 5.4, 2.7 Hz, 1H), 1.49 (s, 9H). ^{13}C NMR (101 MHz, DMSO) δ 161.2 (dd, $J_{C\text{-}F}$ = 32.7, 28.4 Hz), 148.5, 111.4 (dd, $J_{C\text{-}F}$ = 266.6, 253.5 Hz), 91.3 (dd, $J_{C\text{-}F}$ = 21.6, 18.3 Hz), 84.2, 79.6, 73.6, 62.1 (d, $J_{C\text{-}F}$ = 2.9 Hz), 27.4, 19.6 (d, $J_{C\text{-}F}$ = 2.3 Hz). ^{19}F NMR (376 MHz,

DMSO) δ -112.3 (d, $J_{F\text{-}F}$ = 262.1 Hz, 1F), -132.8 (dd, $J_{F\text{-}F}$ = 262.2, $J_{H\text{-}F}$ = 4.7 Hz, 1F). HRMS (ESI) m/z calculated for $C_{12}H_{13}F_2NO_4$ ($-H^+$): 272.0740, found: 272.0738.

tert-butyl 5-(4-((tert-butoxycarbonyl)amino)butyl)-3,3-difluoro-2,4-dioxopyrrolidine-1-carboxylate (**10**)

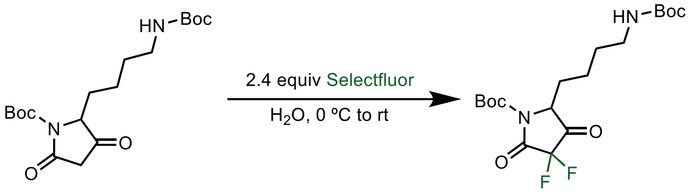

Scheme S26: Synthesis of difluorinated tetramic acid **10**.

Synthesized according to general procedure C with **SI-4** (92.6 mg, 0.250 mmol), Selectfluor (213 mg, 0.600 mmol), and H_2O (1.0 mL) for 48 h. The crude product was purified via flash chromatography (SiO_2, 1.8:1 hexanes:EtOAc) to yield a white solid (84.2 mg, 83%).

NMR characterization was performed in DMSO-d_6 to favor formation of the hydrate from trace water in the NMR solvent and allow the product to be readily characterized as one major species (**10·H₂O**). The NMR are therefore the hydrate of **10**. See section I. General Information for more details. R_f (hexanes: EtOAc 2:1) = 0.23. ^{1}H NMR (400 MHz, DMSO) δ 7.45 (s, 1H), 7.33 (d, J = 1.5 Hz, 1H), 6.76 (t, J = 5.6 Hz, 1H), 3.99 – 3.90 (m, 1H), 2.88 (q, J = 6.2 Hz, 2H), 1.81 – 1.70 (m, 1H), 1.48 (s, 9H), 1.36 (s, 9H), 1.52 – 1.20 (series of obscured m, 5H total). ^{13}C NMR (101 MHz, DMSO) δ 161.4 (dd, $J_{C\text{-}F}$ = 32.7, 28.1 Hz), 155.5, 148.8, 111.7 (dd, $J_{C\text{-}F}$ = 267.2, 252.8 Hz), 91.9 (dd, $J_{C\text{-}F}$ = 21.0, 17.9 Hz), 83.9, 77.3, 64.3 (d, $J_{C\text{-}F}$ = 2.6 Hz), 39.6, 30.4, 29.4, 28.2, 27.4, 23.9. ^{19}F NMR (376 MHz, DMSO) δ -112.0 (d, $J_{F\text{-}F}$ = 261.9 Hz, 1F), -133.6 (dd, $J_{F\text{-}F}$ = 261.7, $J_{H\text{-}F}$ = 5.1 Hz, 1F). HRMS (ESI) m/z calculated for $C_{18}H_{28}F_2N_2O_6$ ($-H^+$): 405.1843, found: 405.1839.

3,3-difluoro-5-phenylpyrrolidine-2,4-dione (**11**)

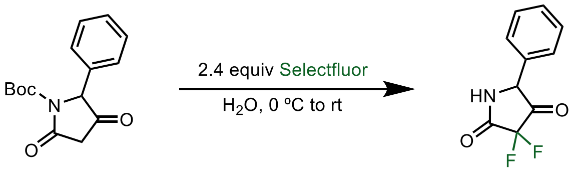

Scheme S27: Synthesis of difluorinated tetramic acid **11**.

Synthesized according to general procedure C with **SI-5** (72.1 mg, 0.250 mmol), Selectfluor (213 mg, 0.600 mmol), and H_2O (1.0 mL) for 22.5 h. The crude product was purified via flash chromatography (SiO_2, 1:1 hexanes:EtOAc) to yield a white solid (45.1 mg, 85%).

NMR characterization was performed in CD_3CN to favor formation of the hydrate from trace water in the NMR solvent and allow the product to be readily characterized as one major species (**11·H₂O**). The NMR are therefore the hydrate of **11**. See section I. General Information for more details. R_f (hexanes: EtOAc 1:1) = 0.28. ^1H NMR (400 MHz, CD_3CN) δ 7.54 – 7.31 (m, 5H), 7.29 (s, 1H), 5.16 (s, 1H), 4.69 (s, 1H), 4.46 (s, 1H). ^{13}C NMR (101 MHz, CD_3CN) δ 164.8 (dd, J_{C-F} = 30.1, 28.0 Hz), 134.4, 129.7, 129.2, 129.1, 113.4 (dd, J_{C-F} = 264.4, 252.7 Hz), 95.5 (dd, J_{C-F} = 22.6, 18.7 Hz), 63.9 (d, J_{C-F} = 2.4 Hz). ^{19}F NMR (376 MHz, CD_3CN) δ -125.0 (dd, J_{F-F} = 262.4, Hz, 1F), -132.2 (dd, J_{F-F} = 262.3, J_{F-H} = 3.2 Hz, 1F). HRMS (ESI) m/z calculated for $C_{10}H_7F_2NO_2$ ($-H^+$): 210.0372, found: 210.0363.

3,3-difluoro-5-(4-nitrobenzyl)pyrrolidine-2,4-dione (**12**)

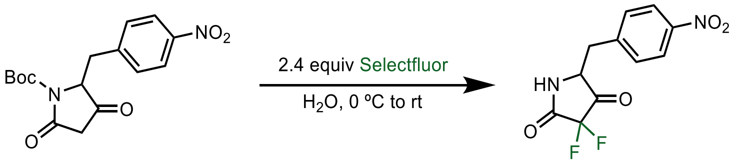

Scheme S28: Synthesis of difluorinated tetramic acid **12**.

Synthesized according to general procedure C with **SI-6** (83.6 mg, 0.250 mmol), Selectfluor (213 mg, 0.600 mmol), and H_2O (1.0 mL) for 48 h. The crude product was purified via flash chromatography (SiO_2, 3:4 hexanes:EtOAc) to yield the title compound as a white solid (35.1 mg, 52%) plus Boc-protected **12** (6.1 mg, 7%).

NMR characterization was performed in DMSO-d_6 to favor formation of the hydrate from trace water in the NMR solvent and allow the product to be readily characterized as one major species (**12·H₂O**). The NMR are therefore the hydrate of **12**. See section I. General Information for more details. R_f (hexanes:EtOAc 3:4) = 0.26. ^1H NMR (400 MHz, DMSO) δ 8.84 (s, 1H), 8.17 (d, J = 8.7 Hz, 2H), 7.51 (d, J = 8.7 Hz, 2H), 7.22 (s, 1H), 7.12 (s, 1H), 3.74 – 3.66 (m, 1H), 3.19 (dd, J = 14.3, 4.4 Hz, 1H), 2.65 (dd, J = 14.2, 9.3 Hz, 1H). ^{13}C NMR (101 MHz, DMSO) δ 163.7 (t, J_{C-F} = 28.9 Hz), 146.2, 146.2, 130.7, 123.4, 112.5 (t, J_{C-F} = 259.4 Hz), 93.6 (t, J_{C-F} = 20.5 Hz), 61.0, 35.9. ^{19}F NMR (376 MHz, DMSO) δ -122.9 (d, J_{F-F} = 258.9 Hz, 1F), -129.8 (dt, J_{F-F} = 259.3, J_{F-H} = 2.8 Hz, 1F). HRMS (ESI) m/z calculated for $C_{11}H_8F_2N_2O_4$ ($-H^+$): 269.0379, found: 269.0373.

3,3-difluoro-5-isopropylpyrrolidine-2,4-dione (**13**)

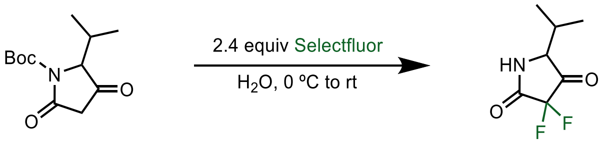

Scheme S29: Synthesis of difluorinated tetramic acid **13**.

Synthesized according to general procedure C with **SI-7** (60.3 mg, 0.250 mmol), Selectfluor (213 mg, 0.600 mmol), and H_2O (1.0 mL) for 21 h. The crude product was purified via flash chromatography (SiO_2, 1:1 hexanes:EtOAc) to yield a white solid (33.2 mg, 75%).

Characterized in $CDCl_3$ predominantly in the ketone form although small amounts of the hydrate are visible in the NMR spectrum. Only the ketone peaks (**13**) are reported. R_f (hexanes:EtOAc 1:1) = 0.27. ^{1}H NMR (400 MHz, $CDCl_3$) δ 8.59 (s, 1H), 4.03 (d, J = 4.6 Hz, 1H), 2.25 (heptd, J = 6.9, 4.6 Hz, 1H), 1.08 (d, J = 7.0 Hz, 3H), 0.99 (d, J = 6.8 Hz, 3H). ^{13}C NMR (101 MHz, $CDCl_3$) δ 196.0 (t, J_{C-F} = 22.2 Hz), 163.2 (t, J_{C-F} = 27.6 Hz), 100.3 (t, J_{C-F} = 260.1 Hz), 65.2 (t, J_{C-F} = 2.5 Hz), 31.4, 18.4, 17.1. ^{19}F NMR (376 MHz, $CDCl_3$) δ -121.0 (d, J_{F-F} = 336.4 Hz, 1F), -124.7 (d, J_{F-F} = 336.5 Hz, 1F). HRMS (ESI) m/z calculated for $C_7H_9F_2NO_2$ (-H^+): 176.0529, found: 176.0521.

tert-butyl 6,6-difluoro-5,7-dioxo-4-azaspiro[2.4]heptane-4-carboxylate (**14**)

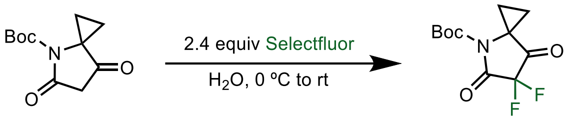

Scheme S30: Synthesis of difluorinated tetramic acid **14**.

Synthesized according to general procedure C with **23** (56.3 mg, 0.250 mmol), Selectfluor (213 mg, 0.600 mmol), and H_2O (1.0 mL) for 1 h. Before column chromatography, the crude reaction mixture was dissolved in $CDCl_3$, benzotrifluoride (20.5 µL, 0.167 mmol, 0.667 equiv) was added as an internal standard, and a ^{19}F NMR was taken of the crude reaction mixture using a 30 second delay between scans (see Figure S62). The crude ^{19}F NMR showed an NMR yield of 66% for **14** and a combined 17% for **24** and **24**•H_2O. The crude product was purified via flash chromatography (SiO_2, 2.5:1 hexanes:EtOAc) to yield **14** as a white solid (40.6 mg, 62%).

Characterized in $CDCl_3$ predominantly in the ketone form. R_f (hexanes:EtOAc 3:1) = 0.20. ^{1}H NMR (400 MHz, $CDCl_3$) δ 2.29 (q, J = 4.7 Hz, 2H), 1.55 (s, 9H), 1.52 (q, J = 5.2 Hz, 2H). ^{13}C NMR (101 MHz, $CDCl_3$) δ 194.8 (t, J_{C-F} = 22.0 Hz), 159.2 (t, J_{C-F} = 28.7 Hz), 148.1, 99.5 (t, J_{C-F} = 260.3 Hz), 86.5, 50.4 (t, J_{C-F} = 3.8 Hz), 28.0, 16.7. ^{19}F NMR (376 MHz, $CDCl_3$) δ -120.9 (s, 2F). HRMS (ESI) m/z calculated for $C_{11}H_{13}F_2NO_4$ (+H_2O -H+): 278.0846, found: 278.0838.

tert-butyl 6,6-difluoro-5,7-dioxo-4-azaspiro[2.4]heptane-4-carboxylate (**24**)

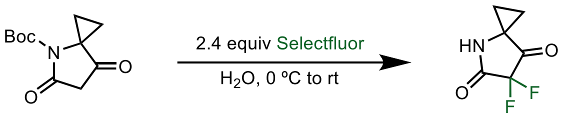

Scheme S31: Synthesis of difluorinated tetramic acid **24**.

Synthesized according to general procedure C with **23** (56.3 mg, 0.250 mmol), Selectfluor (213 mg, 0.600 mmol), and H_2O (1.0 mL) for 20 h. The crude product was purified via flash chromatography (SiO_2, 1:1.5 hexanes:EtOAc) to yield a white solid (28.0 mg, 70%).

NMR characterization was performed in DMSO-d_6 to favor formation of the hydrate (4:1 hydrate:ketone) from trace water in the NMR solvent and allow the product to be readily characterized as a major species (**24·H2O**). The reported NMR are therefore the hydrate of **24**. See section I. General Information for more details. R_f (hexanes:EtOAc 1:1.5) = 0.23. ^{1}H NMR (400 MHz, DMSO) δ 8.79 (s, 1H), 6.76 (s, 2H), 1.03 – 0.84 (m, 2H), 0.72 – 0.60 (m, 2H). ^{13}C NMR (101 MHz, DMSO) δ 163.35 (t, $J_{C\text{-}F}$ = 29.0 Hz), 113.20 (t, $J_{C\text{-}F}$ = 260.0 Hz), 92.65 (t, $J_{C\text{-}F}$ = 20.8 Hz), 44.82 (t, $J_{C\text{-}F}$ = 2.5 Hz), 7.02. ^{19}F NMR (376 MHz, DMSO) δ -127.90 (d, $J_{F\text{-}H}$ = 2.8 Hz, 2F). HRMS (ESI) m/z calculated for $C_6H_5F_2NO_2$ (-H^+): 160.0216, found: 160.0208.

tert-butyl 3,3-difluoro-2,4-dioxopyrrolidine-1-carboxylate (**15**)

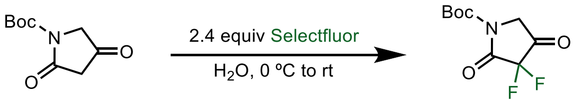

Scheme S32: Synthesis of difluorinated tetramic acid **15**.

Synthesized according to general procedure C with **SI-8** (49.8 mg, 0.250 mmol), Selectfluor (213 mg, 0.600 mmol), and H_2O (1.0 mL) for 1 h. The crude product was purified via flash chromatography (SiO_2, 1.7:1 hexanes:EtOAc) to yield a white solid (43.3 mg, 74%).

NMR characterization was performed in DMSO-d_6 to favor formation of the hydrate from trace water in the NMR solvent and allow the product to be readily characterized as one major species (**15·H2O**). The reported NMR are therefore the hydrate of **15**. See section I. General Information for more details. R_f (hexanes: EtOAc 1:1) = 0.68. ^{1}H NMR (400 MHz, DMSO) δ 7.42 (s, 2H), 3.65 (s, 2H), 1.47 (s, 9H). ^{13}C NMR (101 MHz, DMSO) δ 161.4 (t, $J_{C\text{-}F}$ = 30.7 Hz), 148.7, 111.7 (t, $J_{C\text{-}F}$ = 259.9 Hz), 90.4 (t, $J_{C\text{-}F}$ = 20.6 Hz), 83.8, 53.3, 27.5. ^{19}F NMR (376 MHz, DMSO) δ -127.8 (s, 2F). HRMS (ESI) m/z calculated for $C_9H_{11}F_2NO_4$ (-H^+): 234.0583, found: 234.0580.

benzyl 5-benzyl-3,3-difluoro-2,4-dioxopyrrolidine-1-carboxylate (**16**)

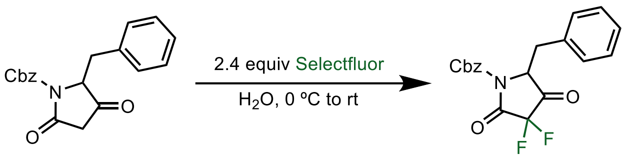

Scheme S33: Synthesis of difluorinated tetramic acid **16**.

Synthesized according to general procedure C with **SI-9** (80.8 mg, 0.250 mmol), Selectfluor (213 mg, 0.600 mmol), and H_2O (1.0 mL) for 20.5 h. The crude product was purified via flash chromatography (SiO_2, 3.5:1 hexanes:EtOAc to 3:1 hexanes:EtOAc) to yield a white solid (67.9 mg, 76%).

NMR characterization was performed in DMSO-d_6 to favor formation of the hydrate from trace water in the NMR solvent and allow the product to be readily characterized as one major species (**16·H$_2$O**). The reported NMR are therefore the hydrate of **16**. See section I. General Information for more details. R_f (hexanes:EtOAc 2:1) = 0.33. ^{1}H NMR (400 MHz, DMSO) δ 7.63 (s, 1H), 7.47 (s, 1H), 7.43 – 7.30 (m, 5H), 7.26 – 7.11 (m, 5H), 5.15 (d, J = 12.4 Hz, 1H), 4.94 (d, J = 12.4 Hz, 1H), 4.37 (q, J = 6.2 Hz, 1H), 3.15 (dd, J = 14.2, 7.9 Hz, 1H), 2.77 (dd, J = 14.3, 6.4 Hz, 1H). ^{13}C NMR (101 MHz, DMSO) δ 161.4 (dd, $J_{C\text{-}F}$ = 33.1, 28.7 Hz), 150.0, 137.8, 134.7, 129.4, 128.4, 128.4, 128.1, 128.1, 126.2, 111.6 (dd, $J_{C\text{-}F}$ = 268.5, 251.8 Hz), 92.1 (dd, $J_{C\text{-}F}$ = 21.4, 17.8 Hz), 68.3, 66.0 (d, $J_{C\text{-}F}$ = 3.3 Hz), 36.5 (d, $J_{C\text{-}F}$ = 2.9 Hz). ^{19}F NMR (376 MHz, DMSO) δ -111.4 (d, $J_{F\text{-}F}$ = 262.9 Hz, 1F), -134.3 (dd, $J_{F\text{-}F}$ = 262.5, $J_{F\text{-}H}$ = 5.2 Hz, 1F). HRMS (ESI) m/z calculated for $C_{19}H_{15}F_2NO_4$ (-H$^+$): 358.0896, found: 358.0891.

(*S*)-5-((*S*)-*sec*-butyl)-3,3-difluoro-2,4-dioxopyrrolidine-1-carboxylate (**17**)

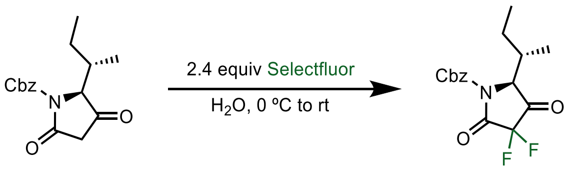

Scheme S34: Synthesis of difluorinated tetramic acid **17**.

Synthesized according to general procedure C with **SI-10** (73.7 mg, 0.250 mmol), Selectfluor (213 mg, 0.600 mmol), and H_2O (1.0 mL) for 41.5 h. The crude product was purified via flash chromatography (SiO_2, 2.2:1 hexanes:EtOAc) to yield a white solid (55.6 mg, 68%, >20:1 dr).

Characterized in DMSO-d_6 predominantly in the ketone form. R_f (hexanes:EtOAc 2:1) = 0.30. ^{1}H NMR (400 MHz, DMSO) δ 7.54 – 7.33 (m, 5H), 5.42 (d, J = 12.4 Hz, 1H), 5.34 (d, J = 12.5 Hz, 1H), 4.67 – 4.61 (m, 1H), 2.13 (heptd, J = 7.0, 3.7 Hz, 1H), 1.54 (dp, J = 14.4, 7.3 Hz, 1H), 1.43

– 1.27 (m, 1H), 0.89 (t, J = 7.4 Hz, 3H), 0.77 (dd, J = 7.1, 2.0 Hz, 4H). ^{13}C NMR (101 MHz, DMSO) δ 193.1 (t, $J_{C\text{-}F}$ = 21.1 Hz), 159.5 (dd, $J_{C\text{-}F}$ = 28.9, 27.0 Hz), 150.0, 134.8, 128.5, 128.5, 128.1, 100.0 (dd, $J_{C\text{-}F}$ = 269.3, 246.4 Hz), 68.8, 67.3, 36.4, 25.4, 13.9 (d, $J_{C\text{-}F}$ = 4.0 Hz), 11.5. ^{19}F NMR (376 MHz, DMSO) δ -110.6 (d, $J_{F\text{-}F}$ = 317.3 Hz, 1F), -127.4 (d, $J_{F\text{-}F}$ = 317.5 Hz, 1F). HRMS (ESI) m/z calculated for $C_{16}H_{17}F_2NO_4$ (-H^+): 324.1053, found: 324.1049.

<u>allyl 3,3-difluoro-2,4-dioxopyrrolidine-1-carboxylate</u> (**18**)

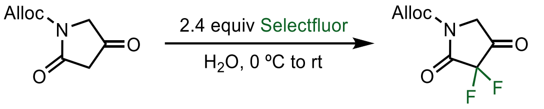

Scheme S35: Synthesis of difluorinated tetramic acid **18**.

Synthesized according to general procedure C with **SI-11** (49.0 mg, 0.268 mmol), Selectfluor (226 mg, 0.637 mmol), and H_2O (1.0 mL) for 0.67 h. The crude product was purified via flash chromatography (SiO_2, 3:2 hexanes:EtOAc) to yield a white solid that was transformed into the hydrate (**18·H2O**) before obtaining a final mass via dissolution and concentration under reduced pressure from wet acetonitrile (white solid, 51.4 mg, 87%).

NMR characterization was performed in CD_3CN to favor formation of the hydrate from trace water in the NMR solvent and allow the product to be readily characterized as one major species (**18·H2O**). The reported NMR are therefore the hydrate of **18**. See section I. General Information for more details. R_f of **18** (hexanes:EtOAc 3:2) = 0.29. ^{1}H NMR (400 MHz, CD_3CN) δ 5.98 (ddt, J = 17.4, 10.7, 5.4 Hz, 1H), 5.42 (dq, J = 17.2, 1.7 Hz, 1H), 5.29 (dq, J = 10.6, 1.5 Hz, 1H), 5.22 (s, 2H), 4.73 (dt, J = 5.5, 1.6 Hz, 2H), 3.77 (s, 2H). ^{13}C NMR (101 MHz, CD_3CN) δ 162.1 (t, $J_{C\text{-}F}$ = 30.7 Hz), 151.2, 132.4, 119.1, 112.3 (t, $J_{C\text{-}F}$ = 259.8 Hz), 92.1 (t, $J_{C\text{-}F}$ = 20.9 Hz), 68.5, 54.2. ^{19}F NMR (376 MHz, CD_3CN) δ -129.2 (s, 2F). HRMS (ESI) m/z calculated for $C_8H_7F_2NO_4$ (-H^+): 218.0270, found: 218.0266.

<u>1-acetyl-5-benzyl-3,3-difluoropyrrolidine-2,4-dione</u> (**19**)

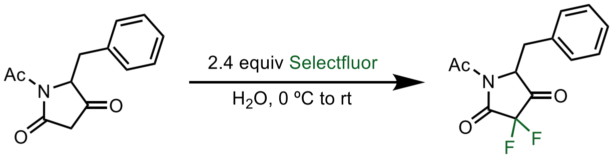

Scheme S36: Synthesis of difluorinated tetramic acid **19**.

Synthesized according to general procedure C with **SI-12** (57.8 mg, 0.250 mmol), Selectfluor (213 mg, 0.600 mmol), and H_2O (1.0 mL) for 0.5 h. The crude product was purified via flash chromatography (SiO_2, 2.5:1 hexanes:EtOAc) to yield a white solid (59.1 mg, 88%).

NMR characterization was performed in DMSO-d_6 to favor formation of the hydrate from trace water in the NMR solvent and allow the product to be readily characterized as one major species (**19·H$_2$O**). The reported NMR are therefore the hydrate of **19**. See section I. General Information for more details. R_f (hexanes:EtOAc 2.5:1) = 0.23. ^{1}H NMR (400 MHz, DMSO) δ 7.58 (s, 1H), 7.42 (d, J = 2.2 Hz, 1H), 7.28 (d, J = 4.2 Hz, 4H), 7.23 – 7.15 (m, 1H), 4.47 – 4.38 (m, 1H), 3.05 (ddd, J = 14.2, 7.8, 2.2 Hz, 1H), 2.71 (dd, J = 14.3, 4.2 Hz, 1H), 2.45 (s, 3H). ^{13}C NMR (101 MHz, DMSO) δ 169.8, 163.0 (dd, J_{C-F} = 33.2, 27.9 Hz), 138.6, 129.4, 128.0, 126.1, 112.4 (dd, J_{C-F} = 269.8, 252.0 Hz), 92.1 (dd, J_{C-F} = 21.4, 17.6 Hz), 64.2 (d, J_{C-F} = 2.8 Hz), 36.0 (d, J_{C-F} = 2.5 Hz), 25.6. ^{19}F NMR (376 MHz, DMSO) δ -111.6 (d, J_{F-F} = 262.5 Hz, 1F), -133.1 (dd, J_{F-F} = 262.7, J_{F-H} = 5.4 Hz, 1F). HRMS (ESI) m/z calculated for $C_{13}H_{11}F_2NO_3$ (-H$^+$): 266.0634, found: 266.0630.

5-benzyl-3,3-difluoropyrrolidine-2,4-dione (**20**)

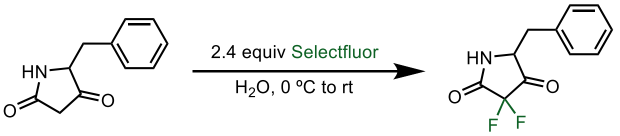

Scheme S37: Synthesis of difluorinated tetramic acid **20**.

Synthesized according to general procedure C with **SI-1** (47.3 mg, 0.250 mmol), Selectfluor (213 mg, 0.600 mmol), and H_2O (1.0 mL) for 18 h. The crude product was purified via flash chromatography (SiO_2, 1:1.15 hexanes:EtOAc) to yield a white solid (56.1 mg, >99%).

NMR characterization was performed in DMSO-d_6 to favor formation of the hydrate from trace water in the NMR solvent and allow the product to be readily characterized as one major species (**20·H$_2$O**). The reported NMR are therefore the hydrate of **20**. See section I. General Information for more details. R_f (hexanes:EtOAc 1:1.1) = 0.28. ^{1}H NMR (400 MHz, DMSO) δ 8.8 (s, 1H), 7.4 – 7.3 (m, 2H), 7.2 – 7.2 (m, 3H), 7.1 (s, 1H), 7.0 (s, 1H), 3.6 (d, J = 9.8 Hz, 0H), 3.1 (dd, J = 14.4, 3.9 Hz, 1H), 2.5 (dd, J = 14.2, 9.9 Hz, 1H). ^{13}C NMR (101 MHz, DMSO) δ 163.8 (t, J_{C-F} = 28.9 Hz), 137.9, 129.1, 128.4, 126.2, 112.6 (t, J_{C-F} = 258.7 Hz), 93.7 (t, J_{C-F} = 20.5 Hz), 61.5, 36.0. ^{19}F NMR (376 MHz, DMSO) δ -123.1 (d, J_{F-F} = 258.7 Hz, 1F), -130.0 (d, J_{F-F} = 258.7 Hz, 1F). HRMS (ESI) m/z calculated for $C_{11}H_9F_2NO_2$ (-H$^+$): 224.0529 found: 224.0521.

3,3-difluoro-5-isobutylpyrrolidine-2,4-dione (**21**)

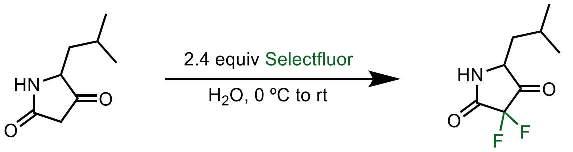

Scheme S38: Synthesis of difluorinated tetramic acid **21**.

Synthesized according to general procedure C with **SI-14** (38.8 mg, 0.250 mmol), Selectfluor (213 mg, 0.600 mmol), and H_2O (1.0 mL) for 39 h. The crude product was purified via flash chromatography (SiO_2, 1.5:1 hexanes:EtOAc) to yield a white solid (41.2 mg, 86%).

NMR characterization was performed in DMSO-d_6 to favor formation of the hydrate from trace water in the NMR solvent and allow the product to be readily characterized as one major species (**21·H2O**). The reported NMR are therefore the hydrate of **21**. See section I. General Information for more details. R_f (hexanes:EtOAc 1.5:1) = 0.18. ^{1}H NMR (400 MHz, DMSO) δ 8.89 (s, 1H), 6.91 (s, 1H), 6.83 (s, 1H), 3.42 – 3.37 (m, 1H), 1.80 – 1.61 (m, 1H), 1.43 (ddd, J = 13.8, 9.6, 4.1 Hz, 1H), 1.26 – 1.13 (m, 1H), 0.89 (d, J = 6.6 Hz, 3H), 0.84 (d, J = 6.5 Hz, 3H). ^{13}C NMR (101 MHz, DMSO) δ 163.9 (t, $J_{C\text{-}F}$ = 28.7 Hz), 112.8 (dd, $J_{C\text{-}F}$ = 259.3, 257.7 Hz), 93.8 (t, $J_{C\text{-}F}$ = 20.3 Hz), 57.8, 38.3, 23.6 (d, $J_{C\text{-}F}$ = 6.5 Hz), 21.4. ^{19}F NMR (376 MHz, DMSO) δ -125.0 (d, $J_{F\text{-}F}$ = 258.7 Hz, 1F), -128.5 (d, $J_{F\text{-}F}$ = 258.0 Hz, 1F). HRMS (ESI) m/z calculated for $C_8H_{11}F_2NO_2$ (-H^+): 190.0685, found: 190.0681.

Attempted synthesis of (**22**)

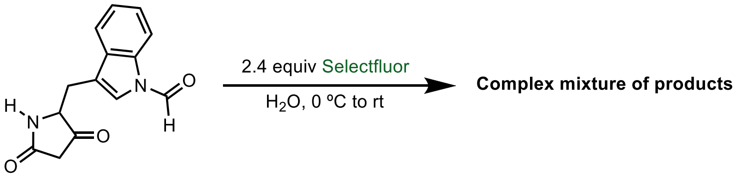

Scheme S39: Attempted Synthesis of difluorinated tetramic acid **22**.

General procedure C was followed with **SI-16** (47.3 mg, 0.250 mmol), Selectfluor (213 mg, 0.600 mmol), and H_2O (1.0 mL) for 2.5 h. The crude product was worked up as described in general procedure C and analyzed via ^{19}F-NMR with 20.5 µL of α,α,α-trifluorotoluene as an internal standard. A copy of this crude NMR is included below showing the extent of decomposition that was observed.

III. SI References

1. Jouin, P.; Castro, B.; Nisato, D. Stereospecific synthesis of *N*-protected statine and its analogues via chiral tetramic acid. *J. Chem. Soc., Perkin Trans. 1.*, **1987**, *6*, 1177−1182. DOI: 10.1039/P19870001177.
2. Aikawa, K.; Miyawaki, T.; Hitaka, T.; Imai, Y.N.; Hara, T.; Miyazaki, J.; Yamaoka, M.; Kusaka, M.; Kanzaki, N.; Tasaka, A.; Shiraishi, M.; Yamamoto, S.; Synthesis and biological evaluation of novel selective androgen receptor modulators (SARMs). Part I. *Bioorg. Med. Chem.*, **2015**, *23*(10), 2568–2578. DOI: 10.1016/j.bmc.2015.03.032.
3. Brea, R.J.; Lopez-Deber, M.P; Castedo, L.; Granja, J.R. Synthesis of ω-(Hetero) arylalkynylated α-Amino Acid by Sonogashira-Type Reactions in Aqueous Media. *J. Org. Chem.,* **2006**, *71*(20)*,* 7870-7873. DOI: 10.1021/jo061300n.
4. Schuler, S.; Einsiedler, M.; Evers, J.K.; Malay, M.; Uka, V.; Schneider, S.; Gulder, T.A.M. Expanding Polycyclic Tetramate Macrolactam (PoTeM) core structure diversity by chemo-enzymatic synthesis and bioengineering. *Angew. Chem. Int. Ed*., **2025**, *64*(13), e202420335. DOI: 10.1002/anie.202420335.

V. Copies of NMR Spectra

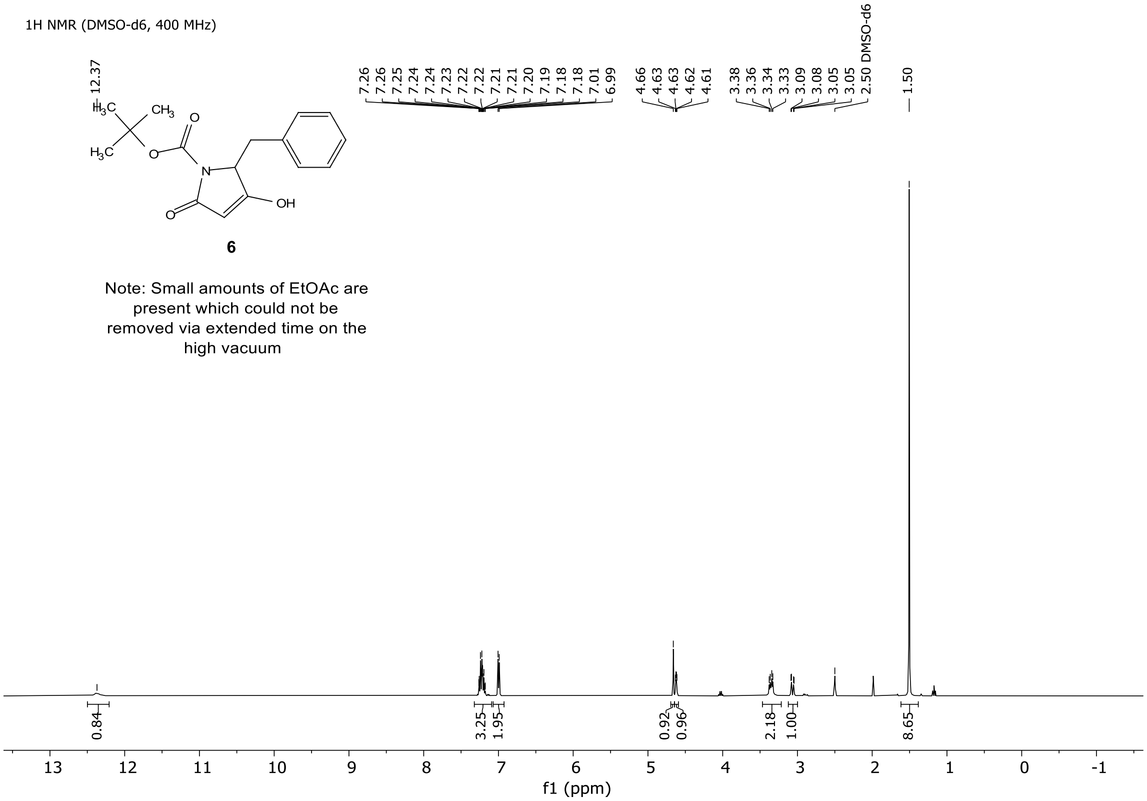

Figure S1: ^{1}H NMR of **6.**

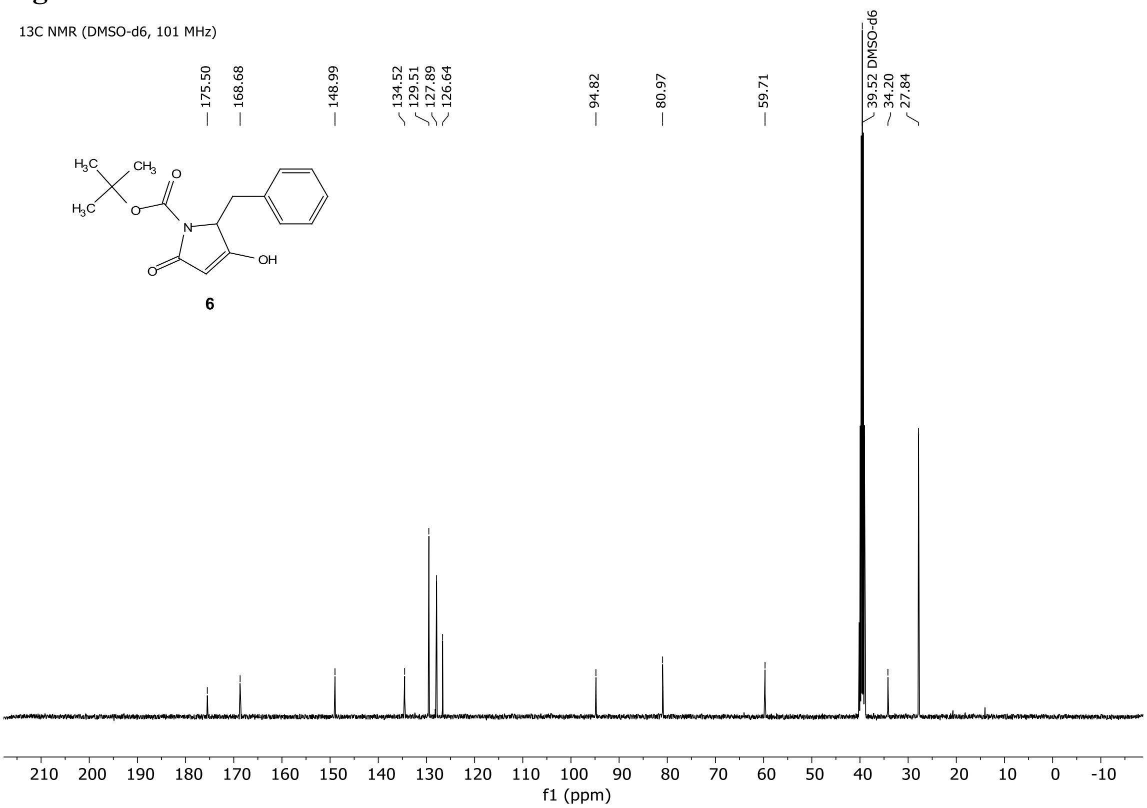

Figure S2: ^{13}C NMR of **6.**

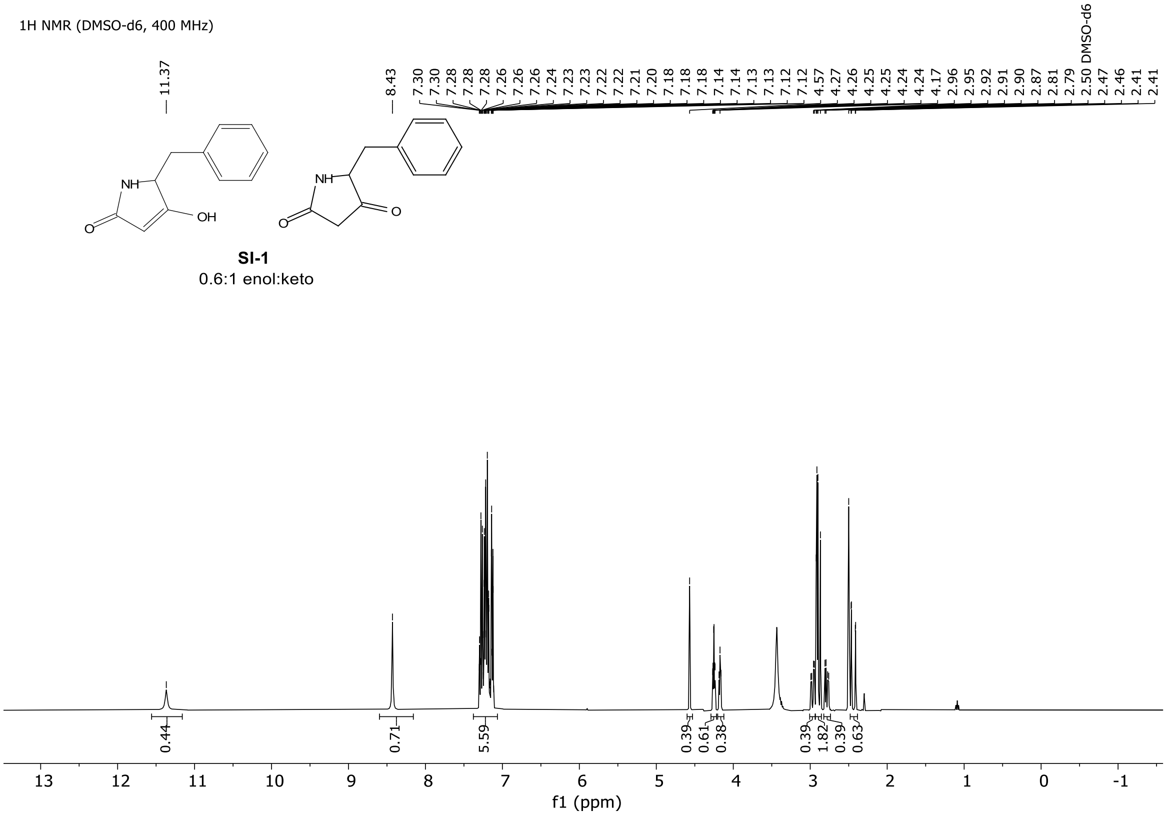

Figure S3: ^{1}H NMR of **SI-1.**

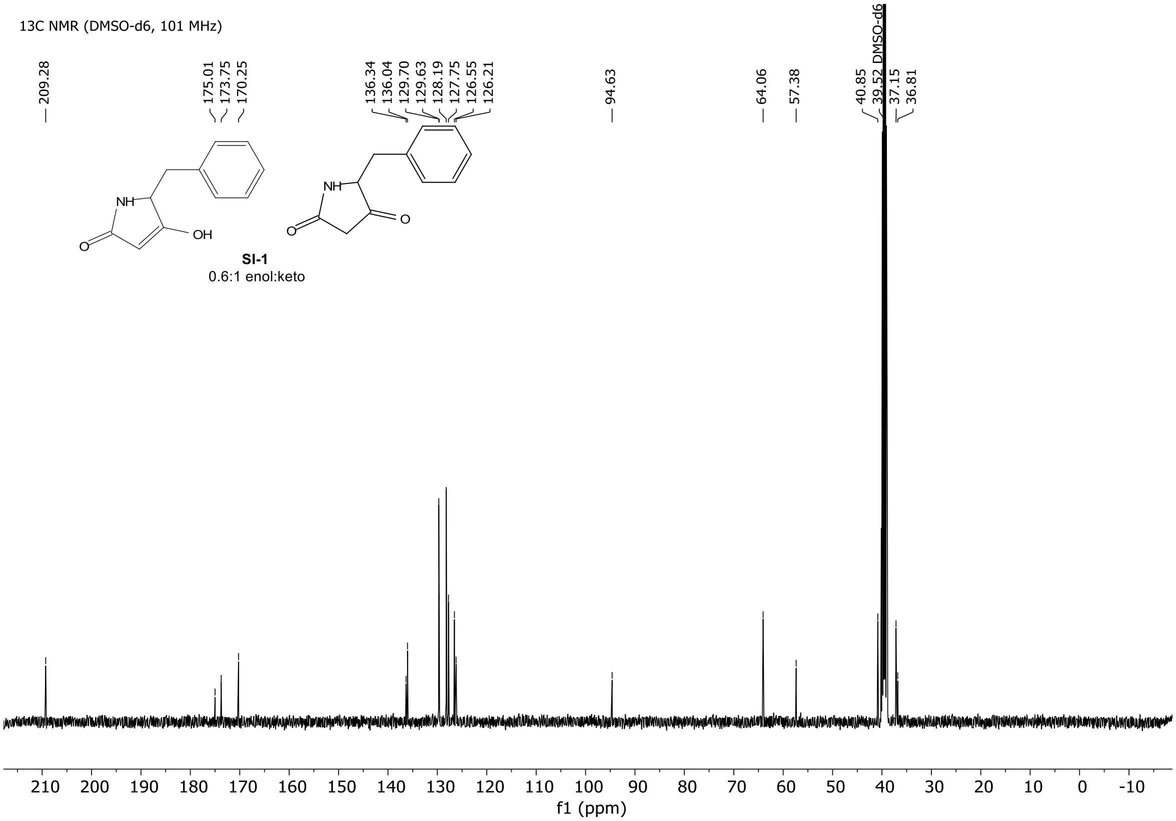

Figure S4: ^{13}C NMR of **SI-1.**

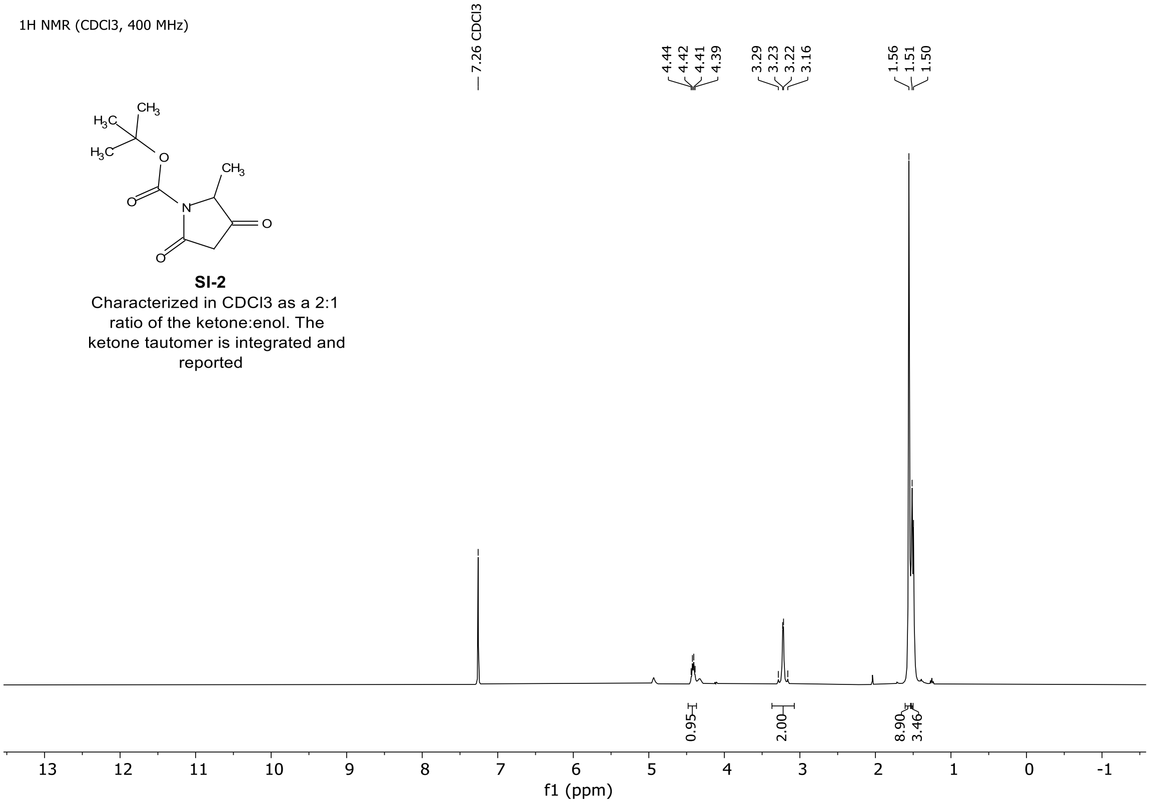

Figure S5: ^{1}H NMR of **SI-2**.

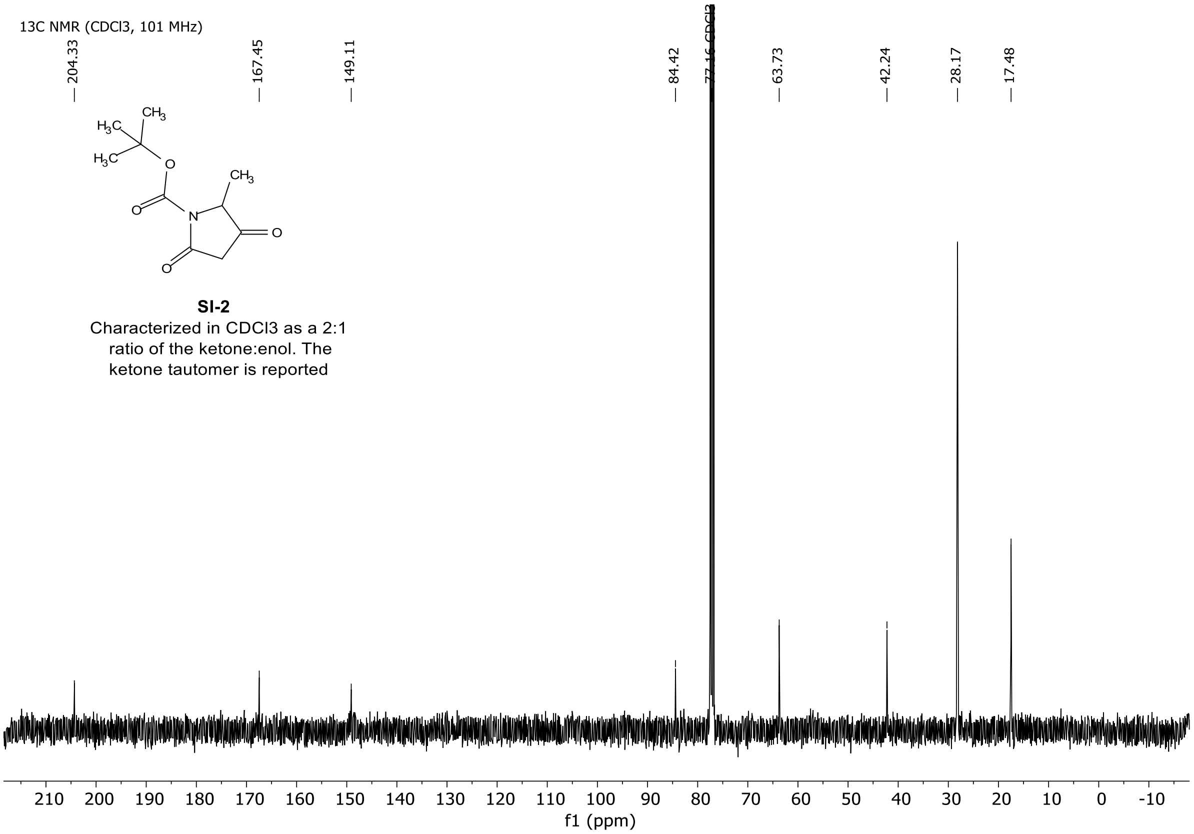

Figure S6: ^{13}C NMR of **SI-2**.

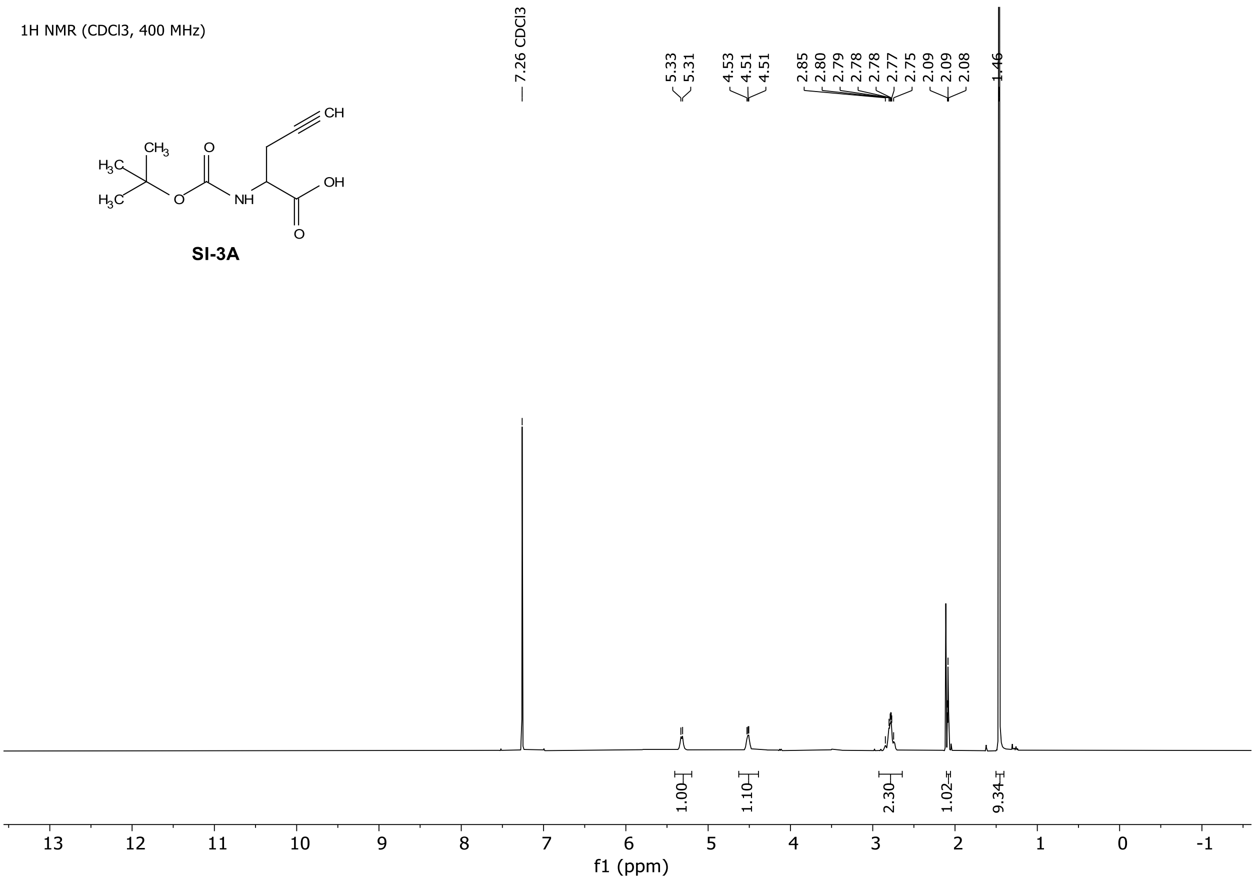

Figure S7: ^{1}H NMR of **SI-3A**.

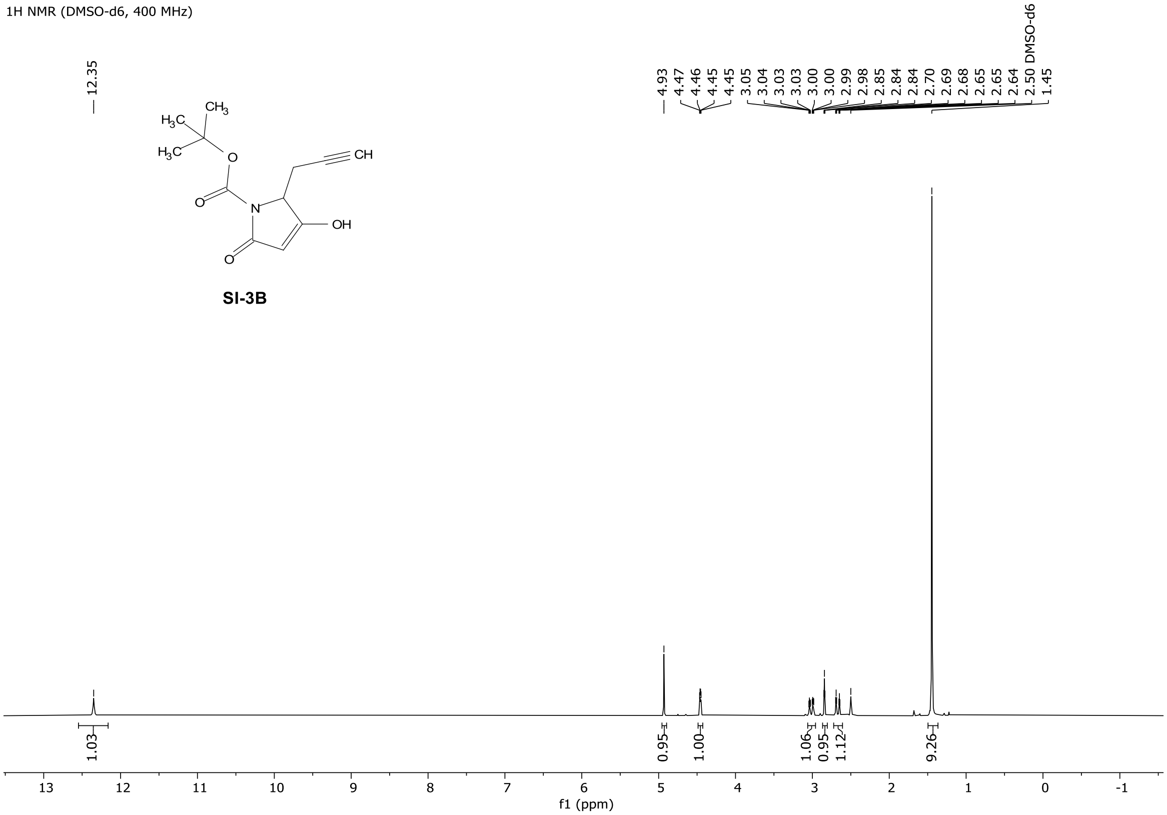

Figure S8: ^{1}H NMR of **SI-3B**.

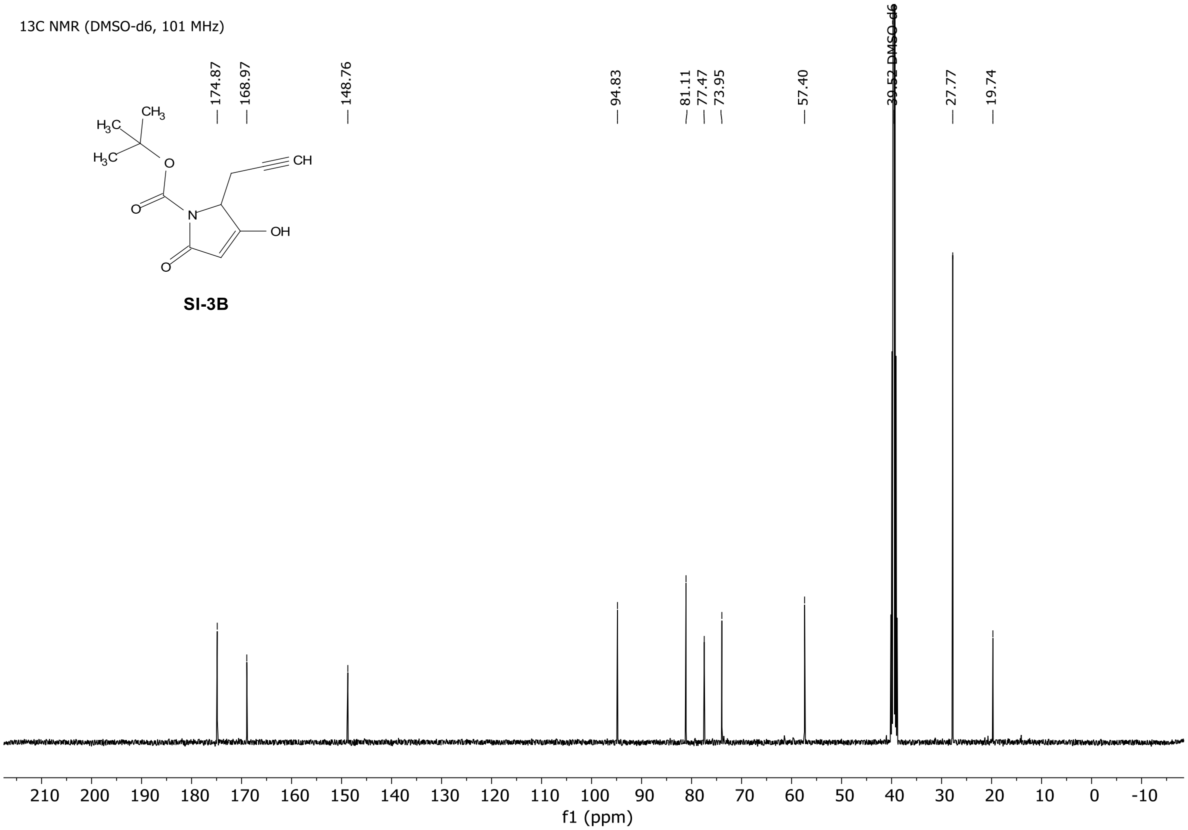

Figure S9: ^{13}C NMR of **SI-3B**.

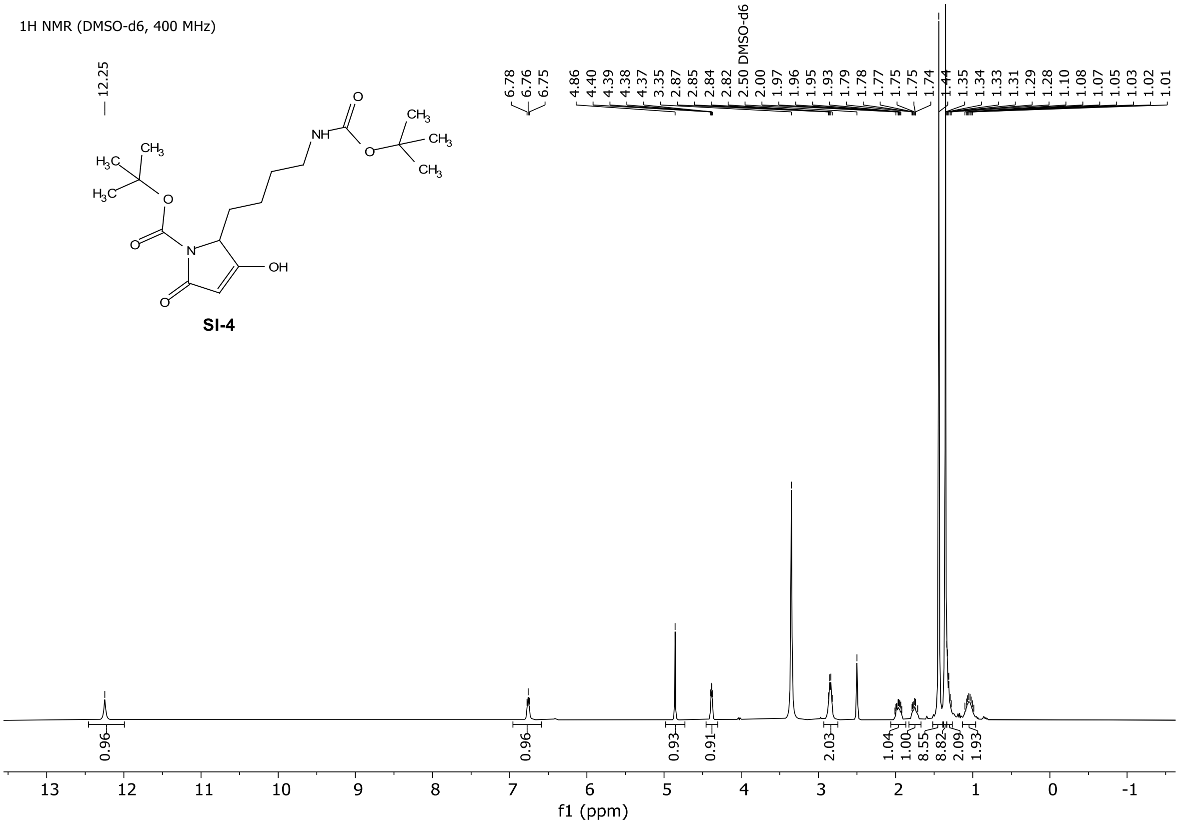

Figure S10: ^{1}H NMR of **SI-4**.

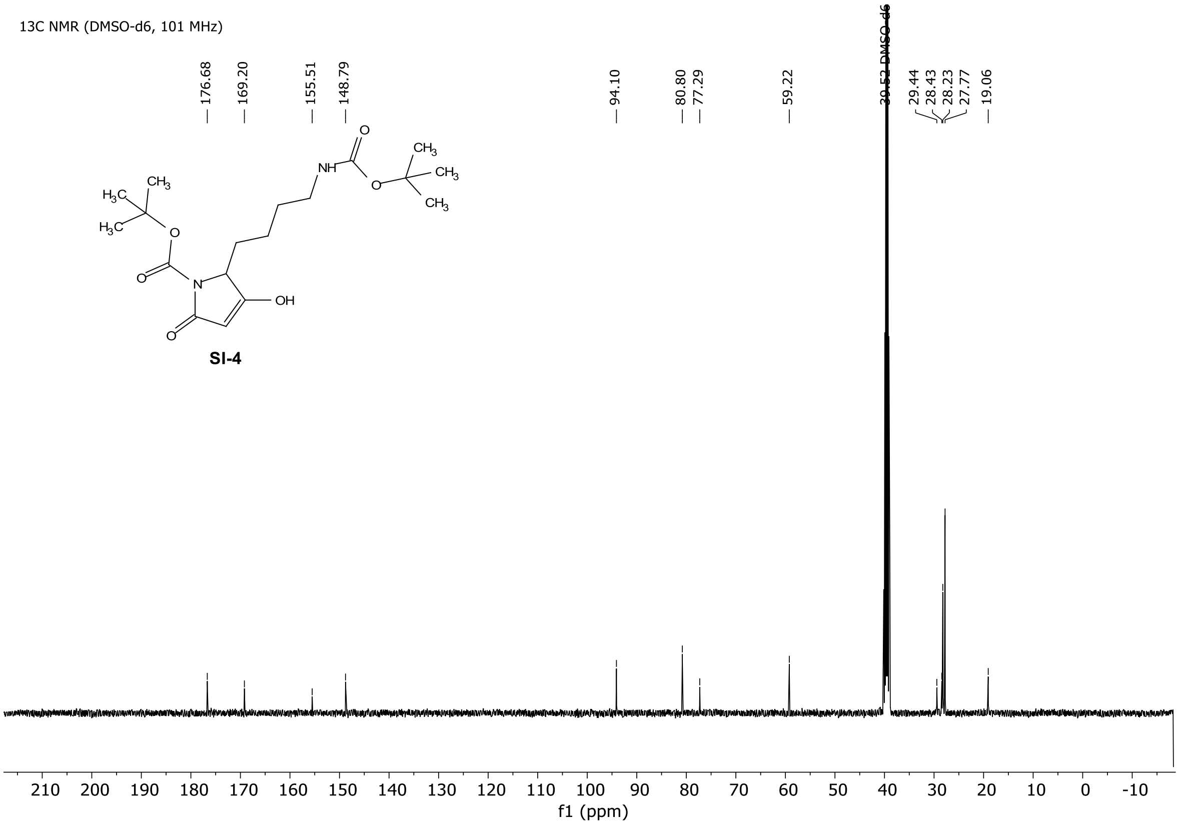

Figure S11: ^{13}C NMR of **SI-4**.

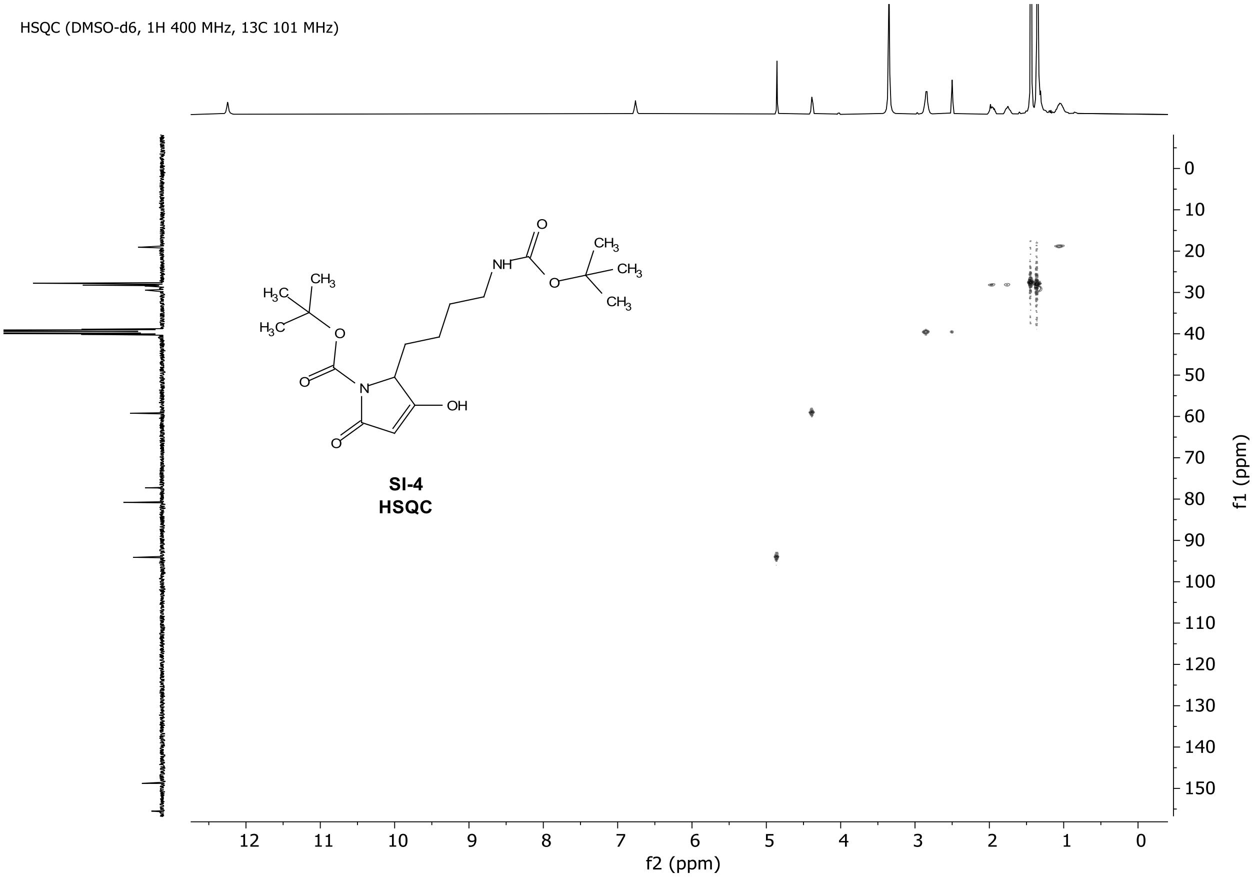

Figure S12: HSQC NMR of **SI-4**.

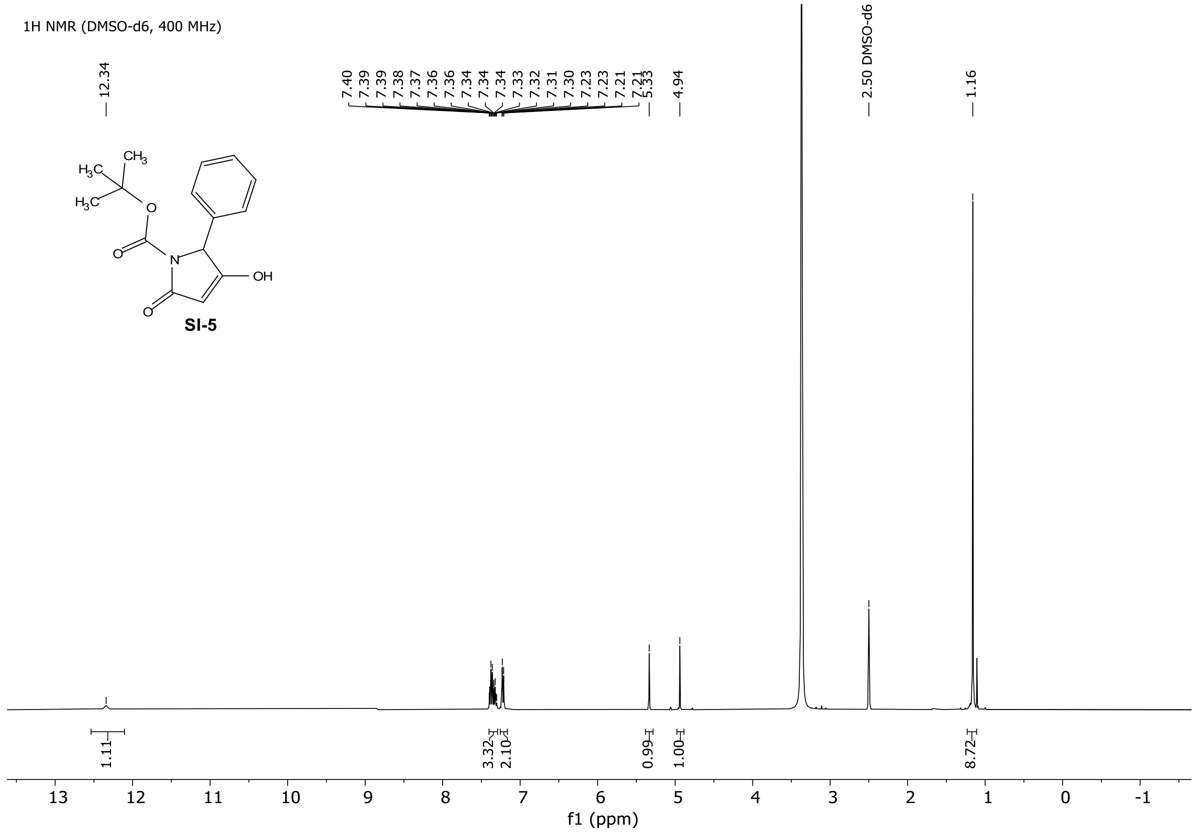

Figure S13: ^{1}H NMR of **SI-5**.

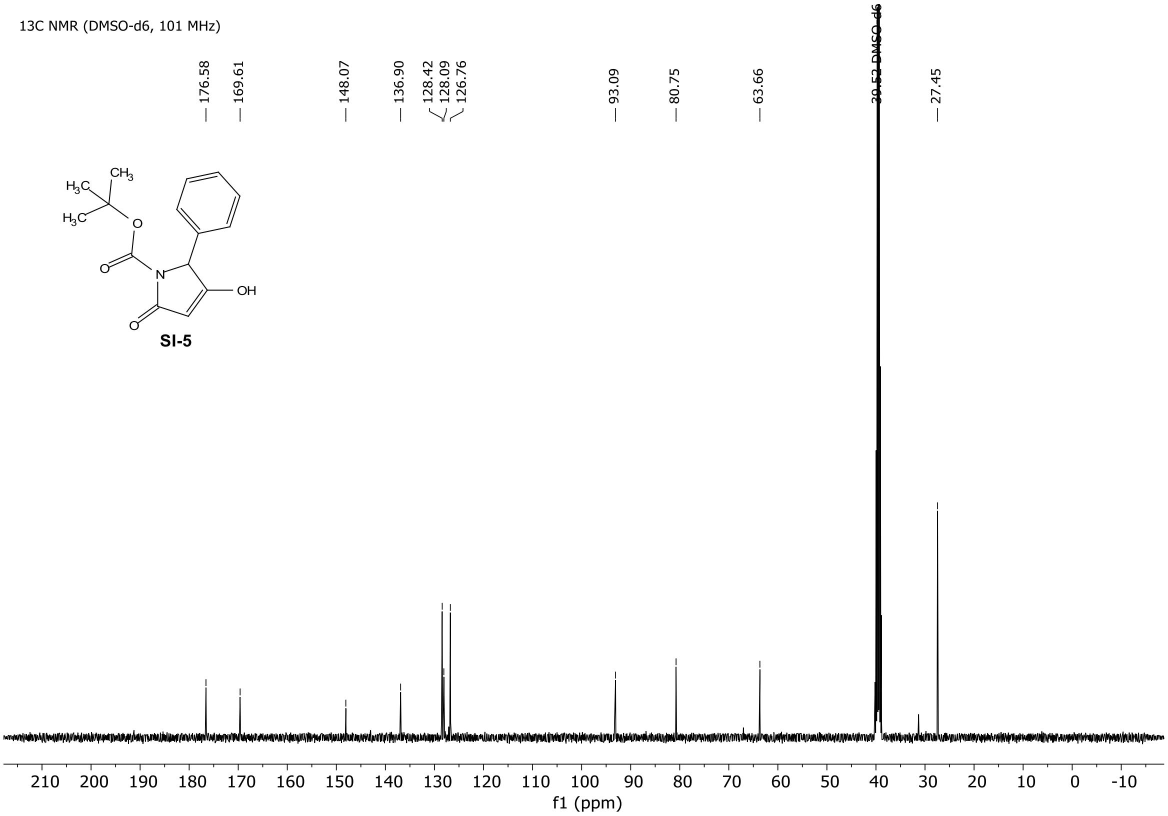

Figure S14: ^{13}C NMR of **SI-5**.

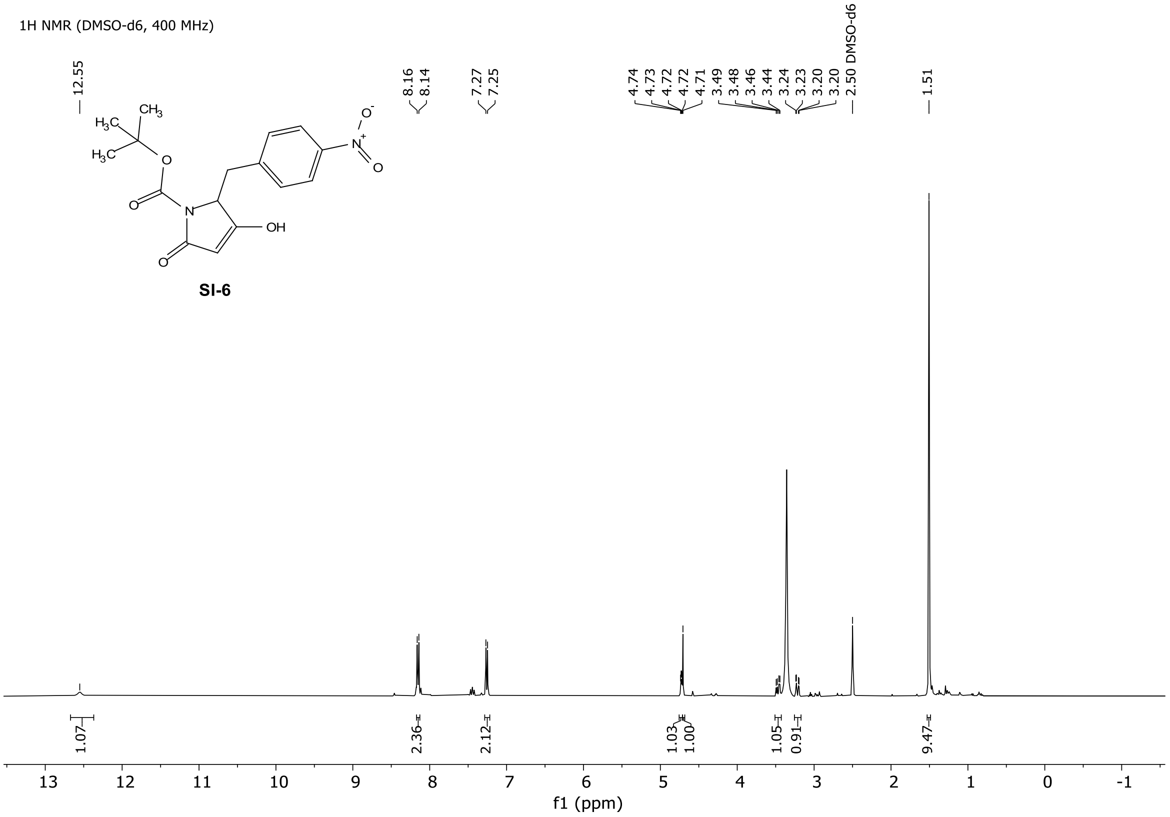

Figure S15: ^{1}H NMR of **SI-6**.

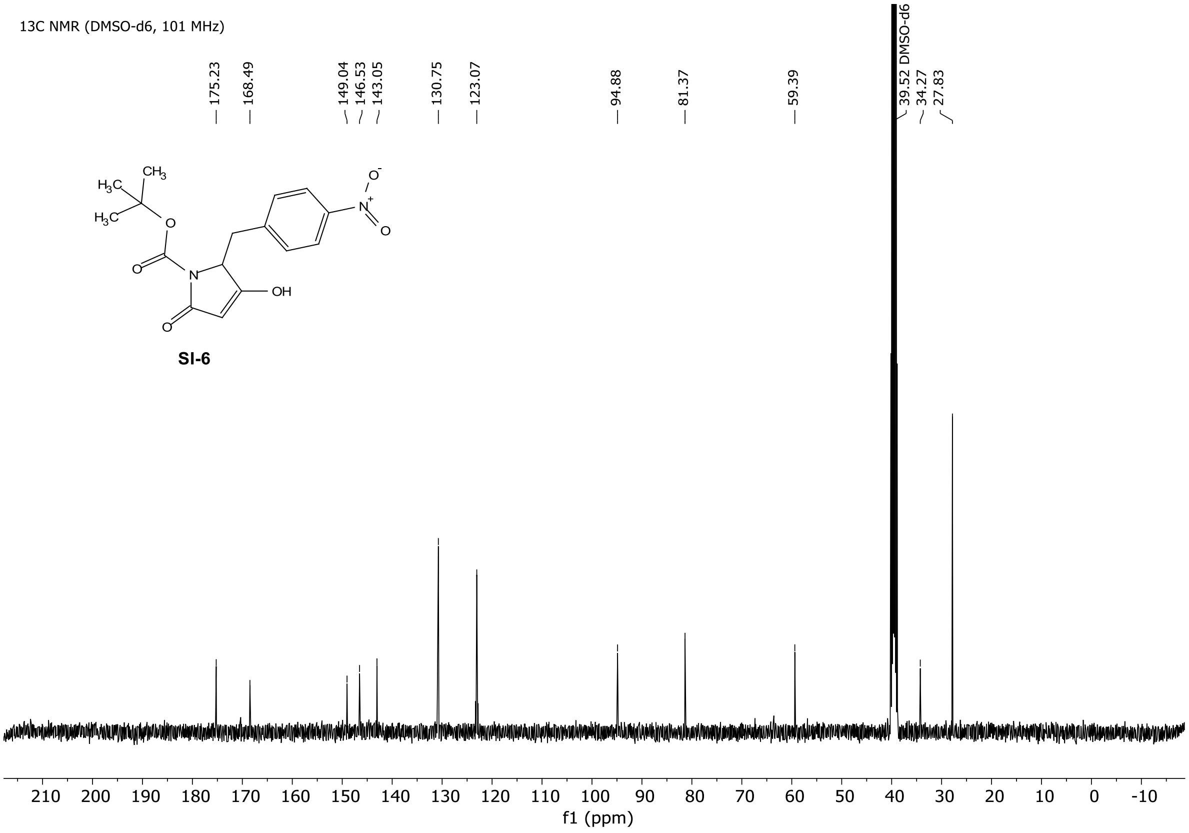

Figure S16: ^{13}C NMR of **SI-6**.

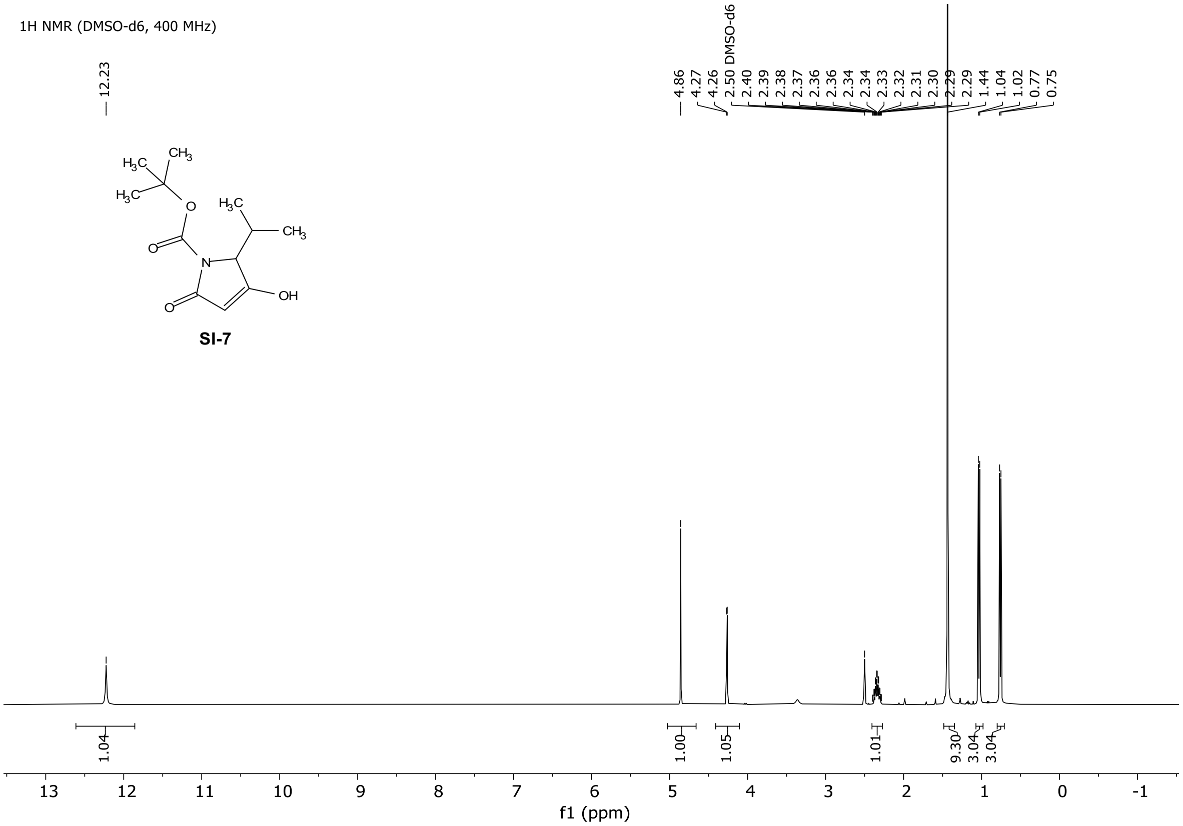

Figure S17: ^{1}H NMR of **SI-7**.

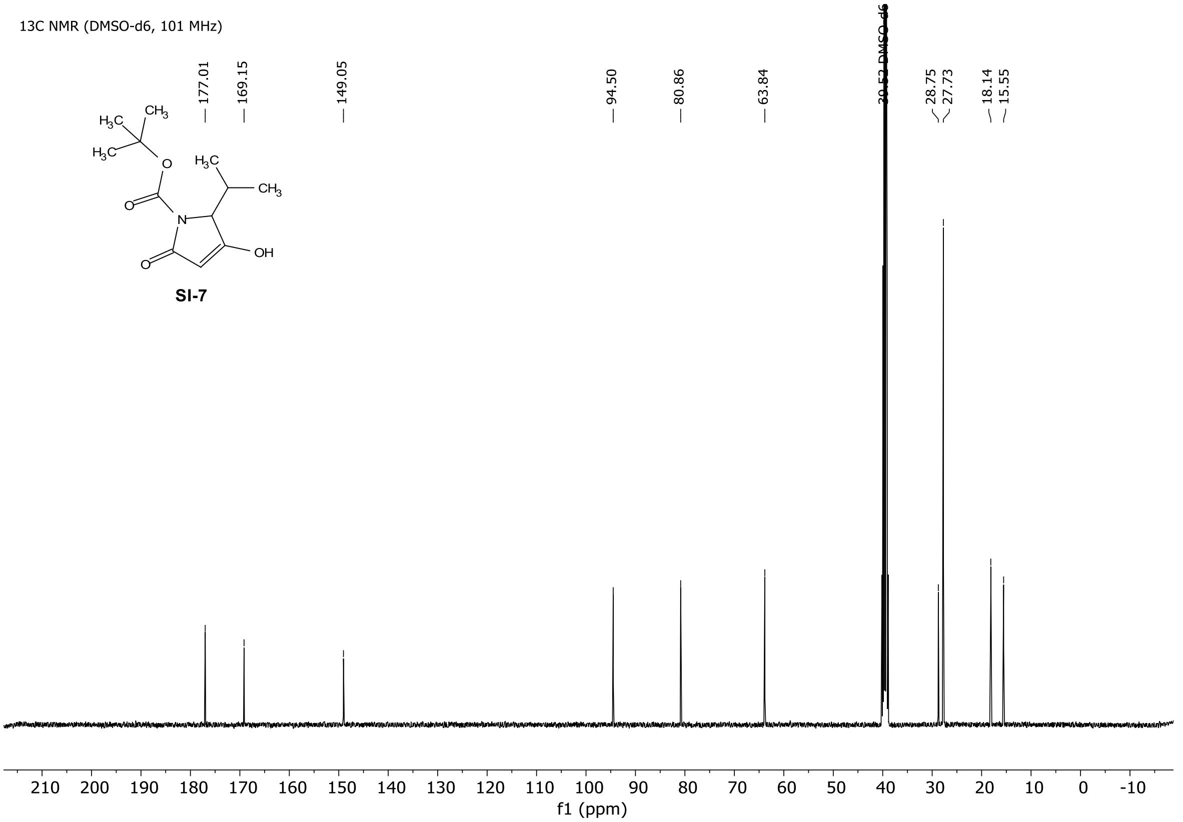

Figure S18: ^{13}C NMR of **SI-7**.

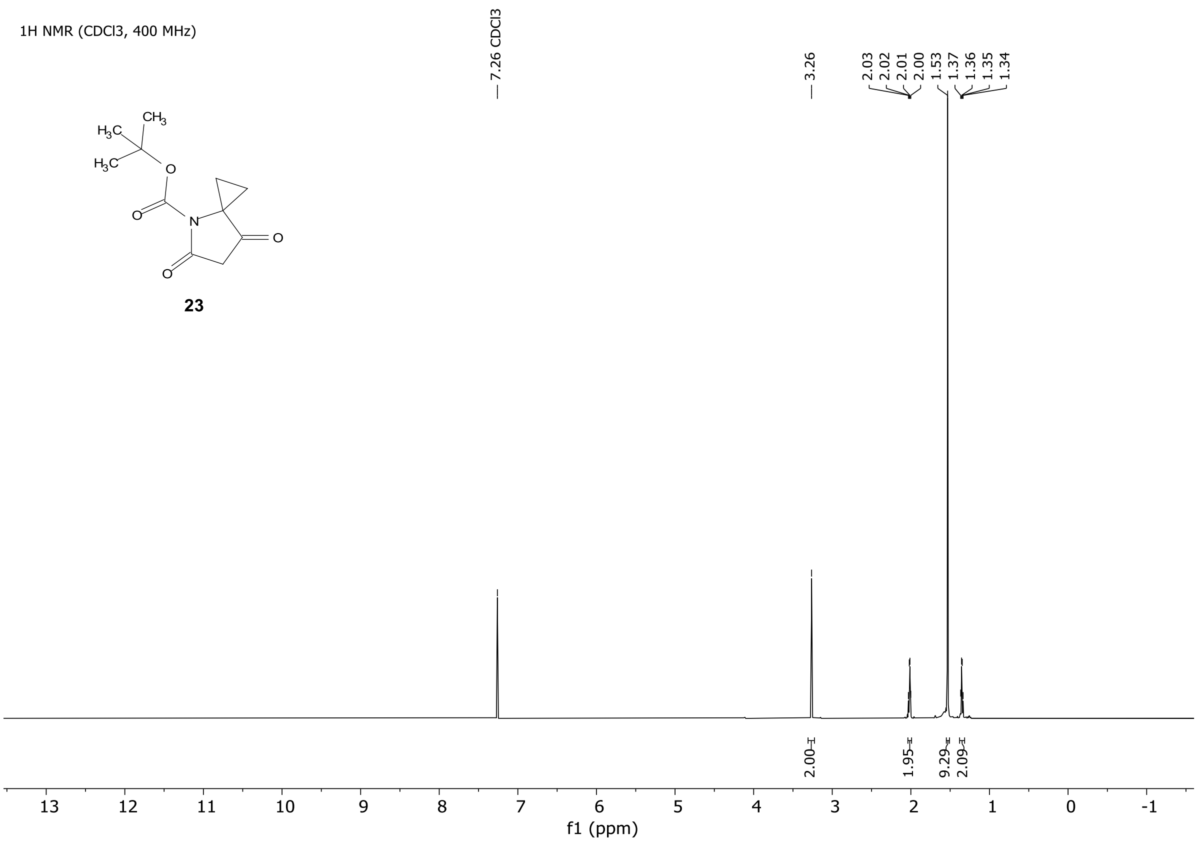

Figure S19: ^{1}H NMR of **23**.

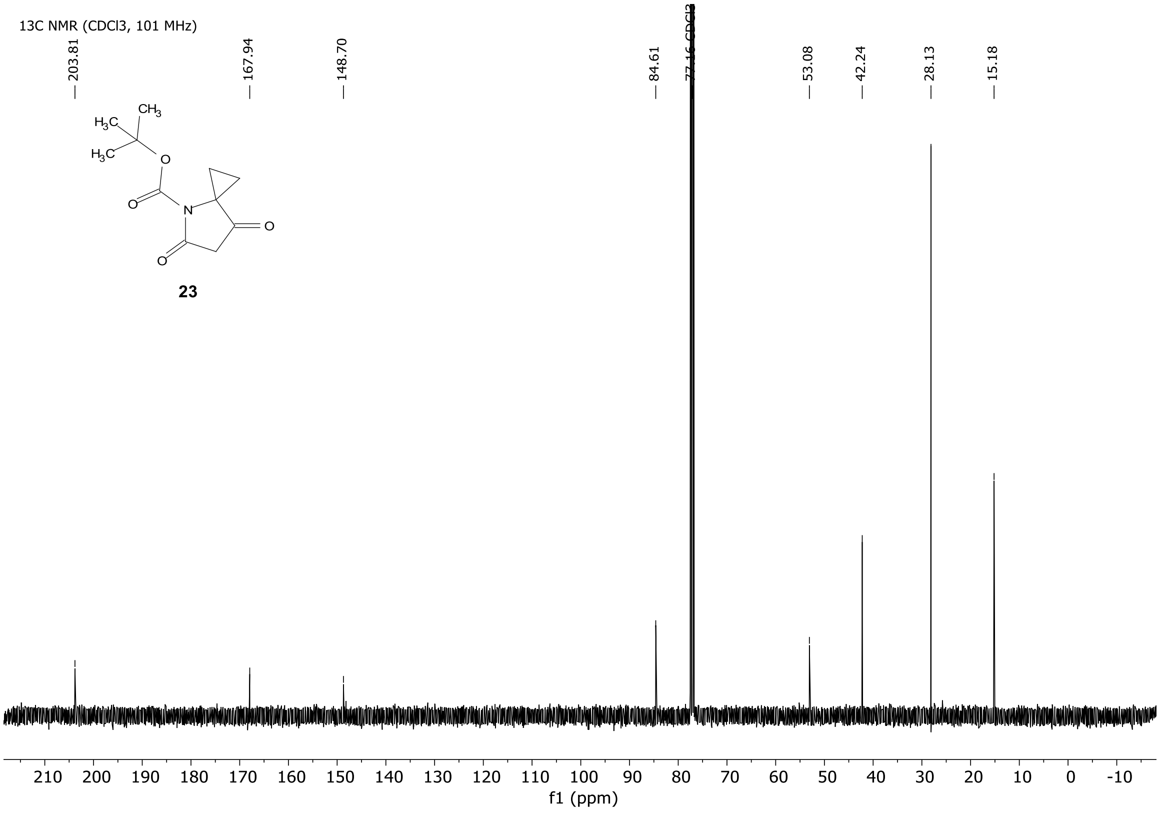

Figure S20: ^{13}C NMR of **23**.

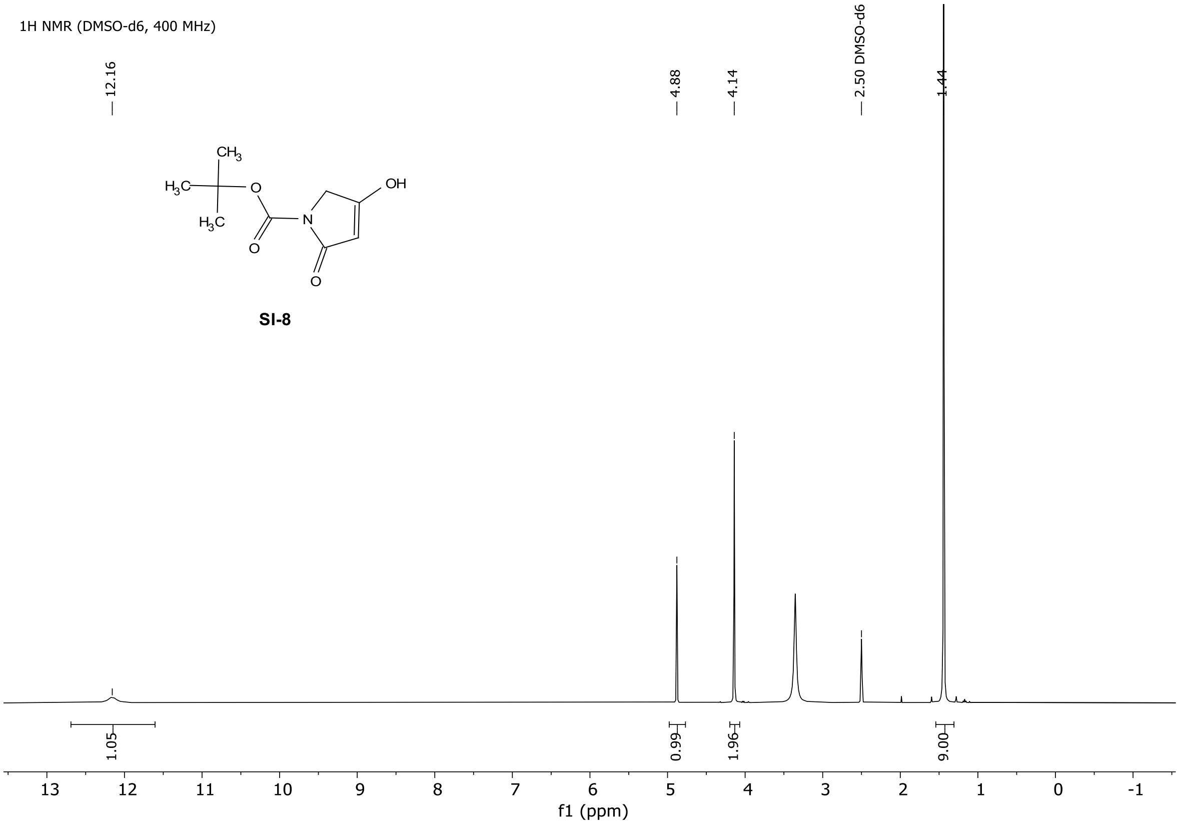

Figure S21: ^{1}H NMR of **SI-8**.

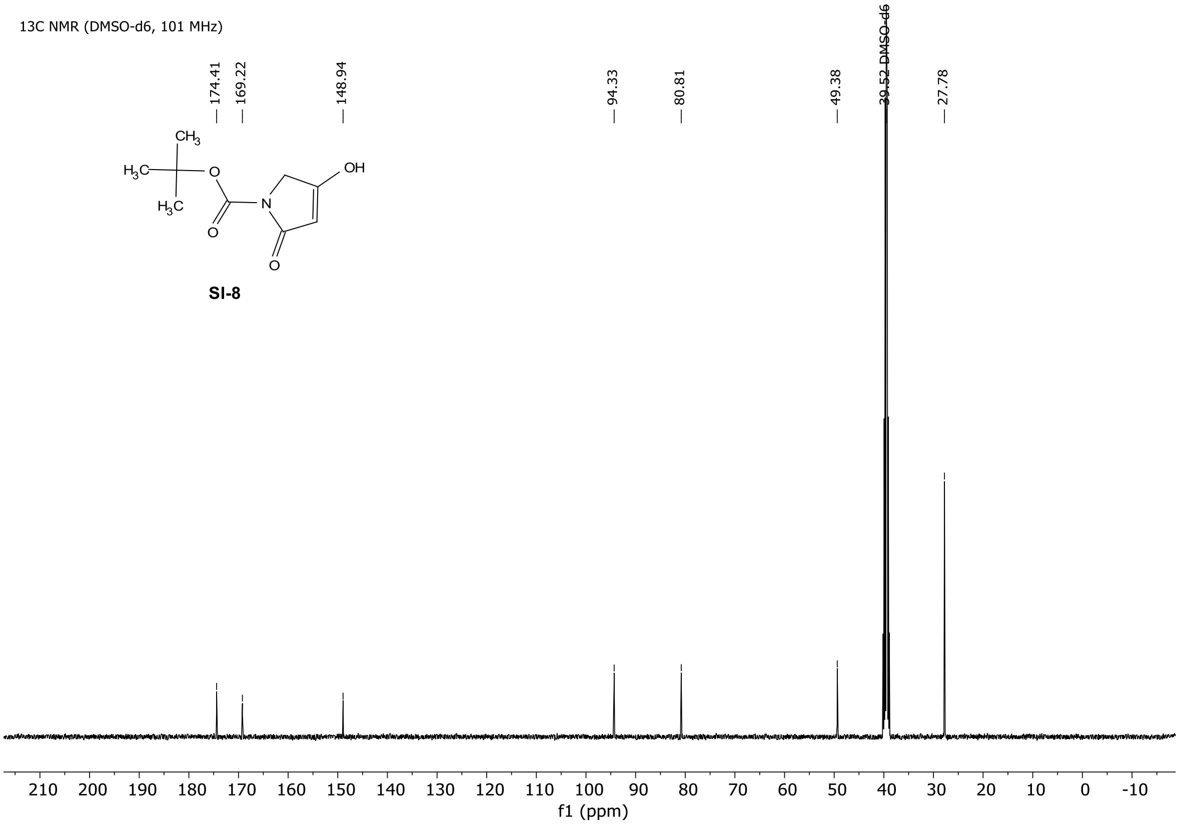

Figure S22: ^{13}C NMR of **SI-8**.

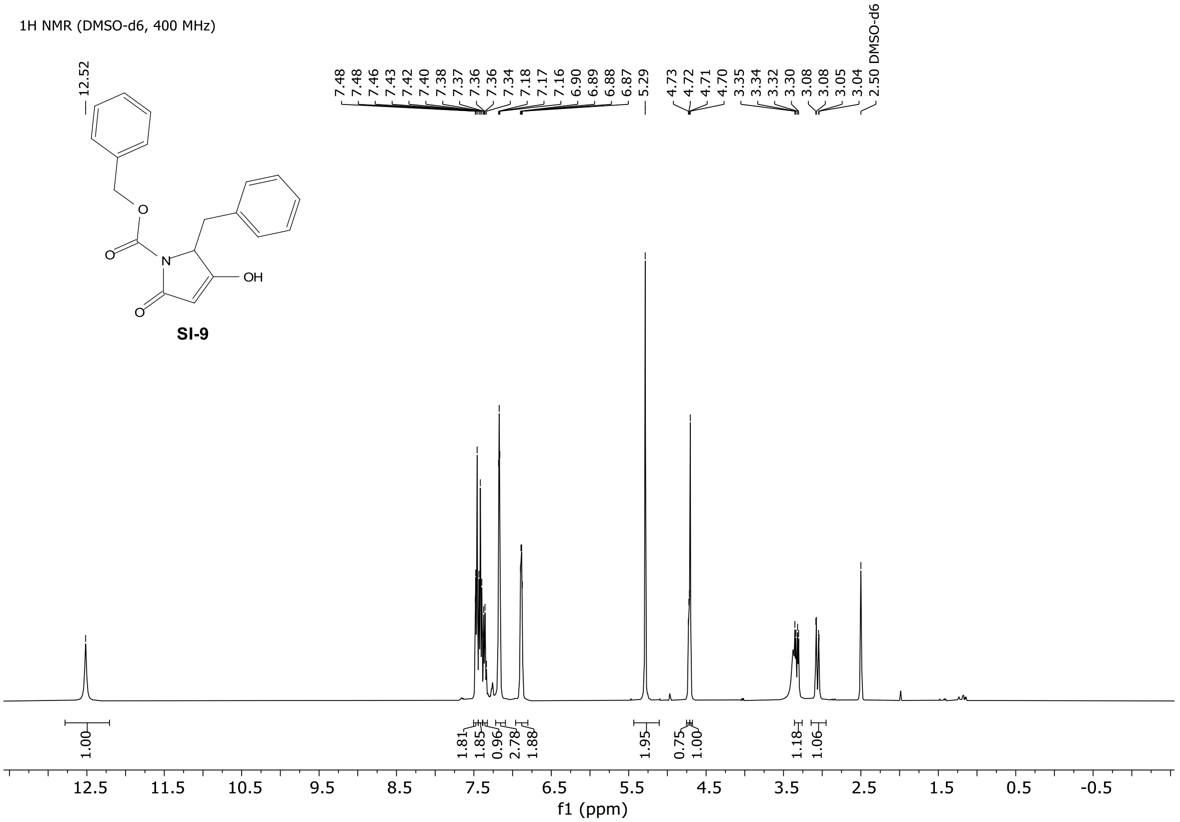

Figure S23: ^{1}H NMR of **SI-9**.

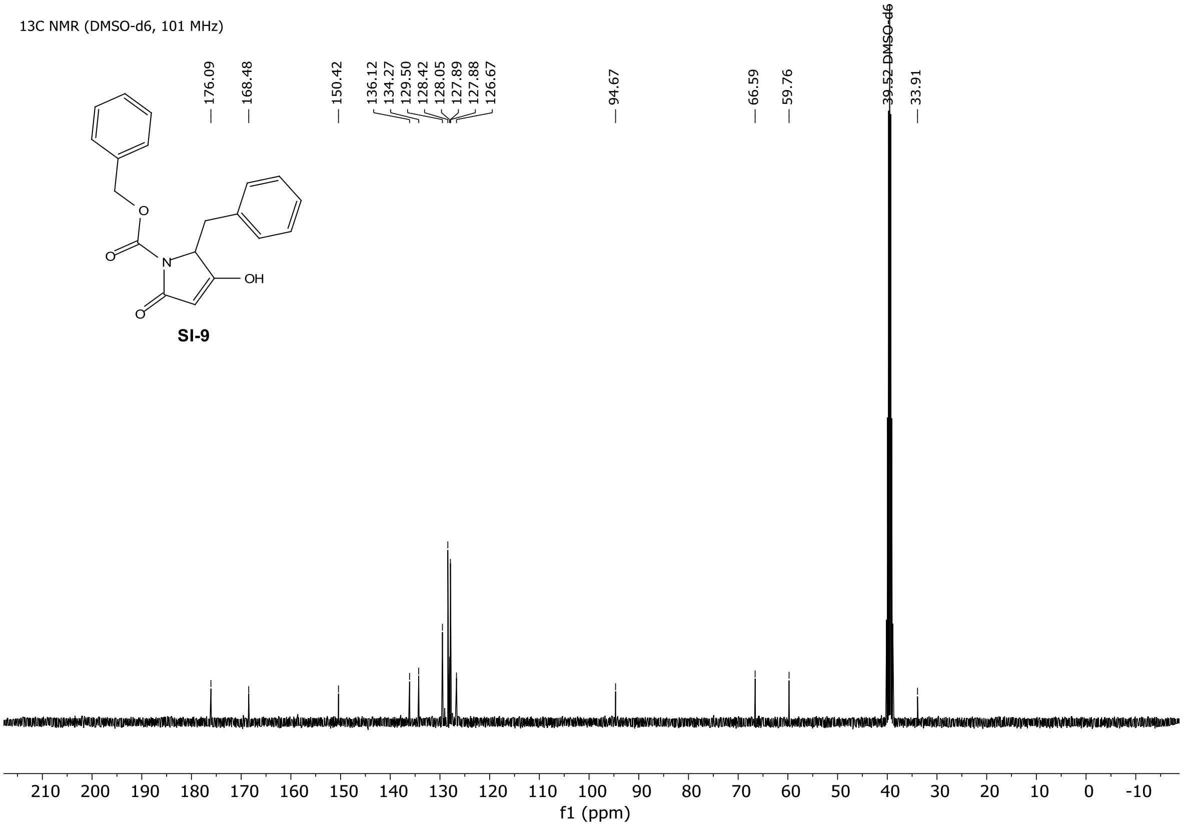

Figure S24: ^{13}C NMR of **SI-9**.

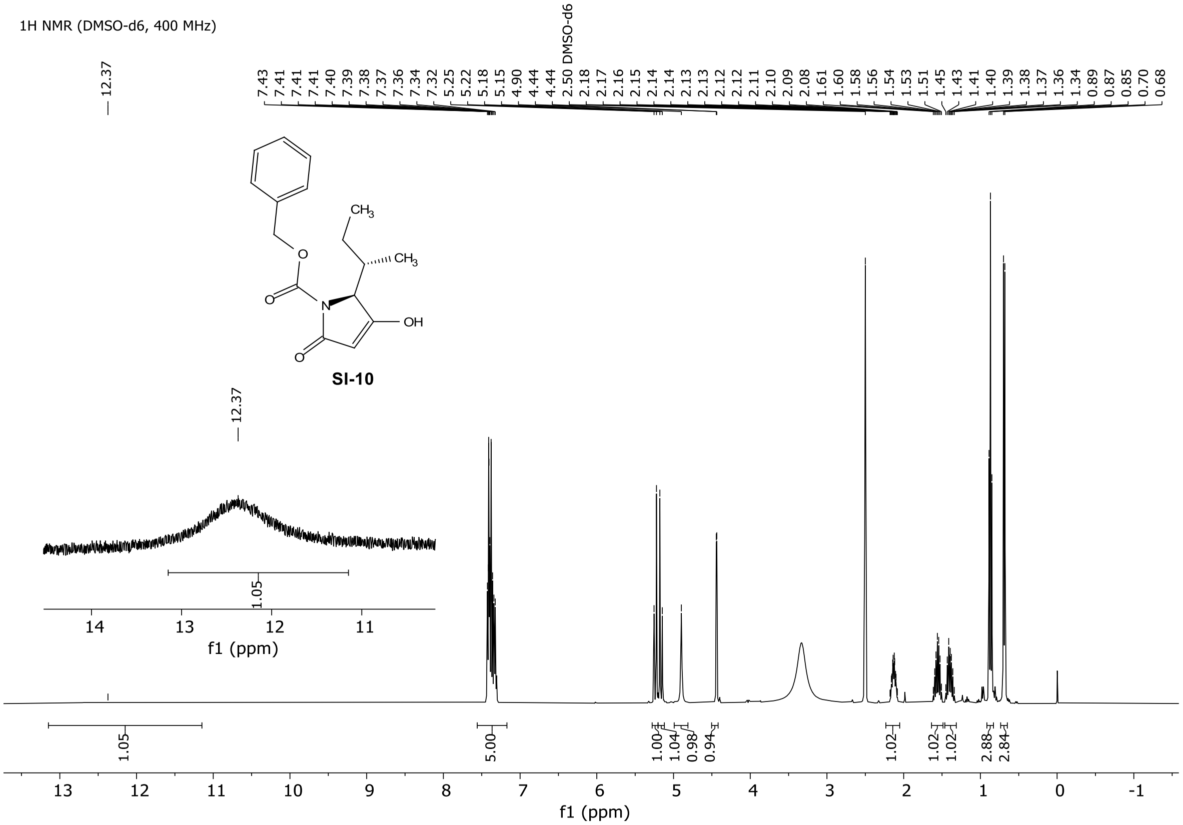

Figure S25: ^{1}H NMR of **SI-10**.

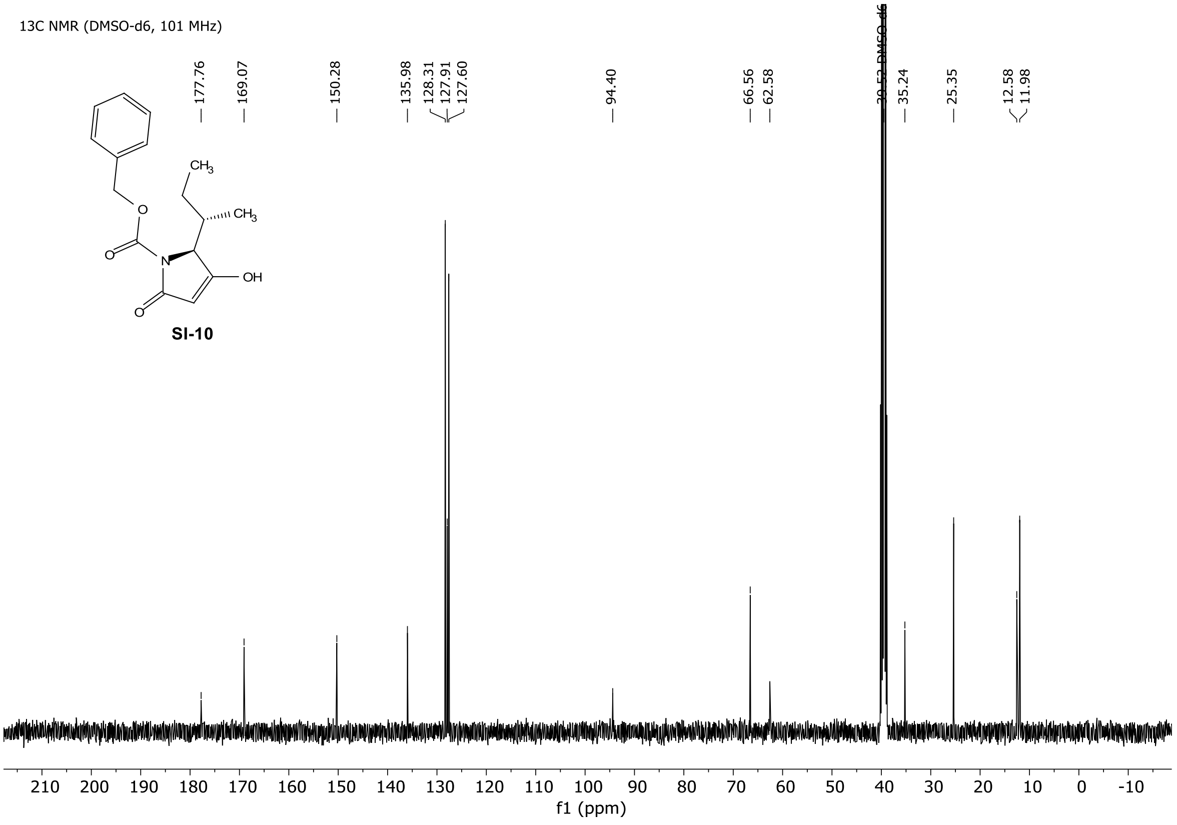

Figure S26: ^{13}C NMR of **SI-10**.

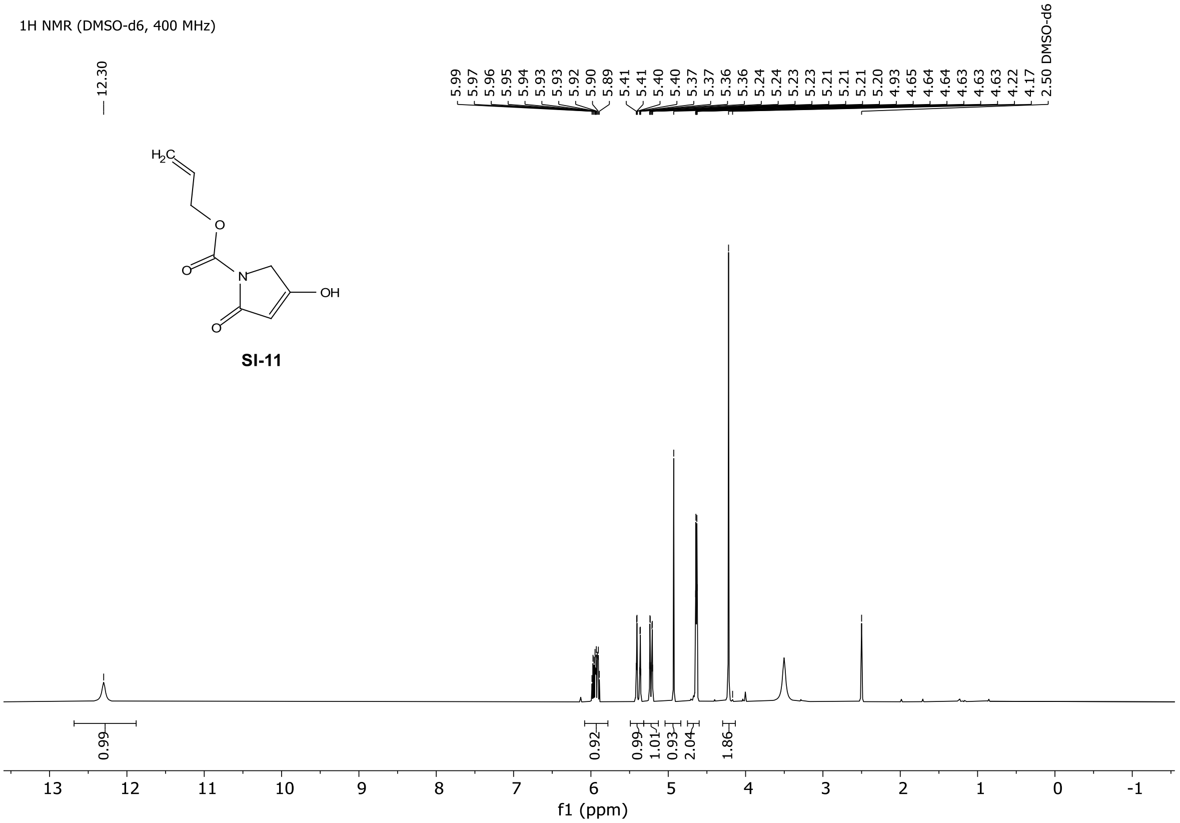

Figure S27: ^{1}H NMR of **SI-11**.

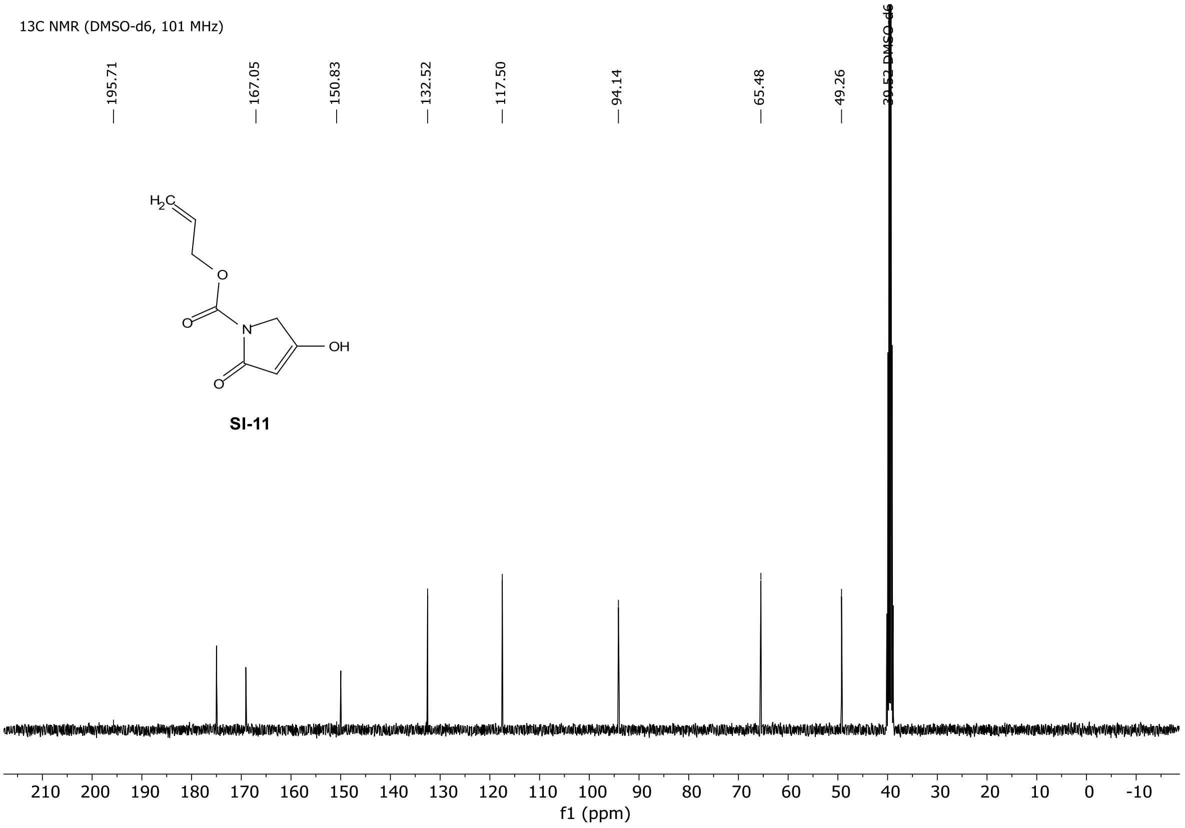

Figure S28: ^{13}C NMR of **SI-11**.

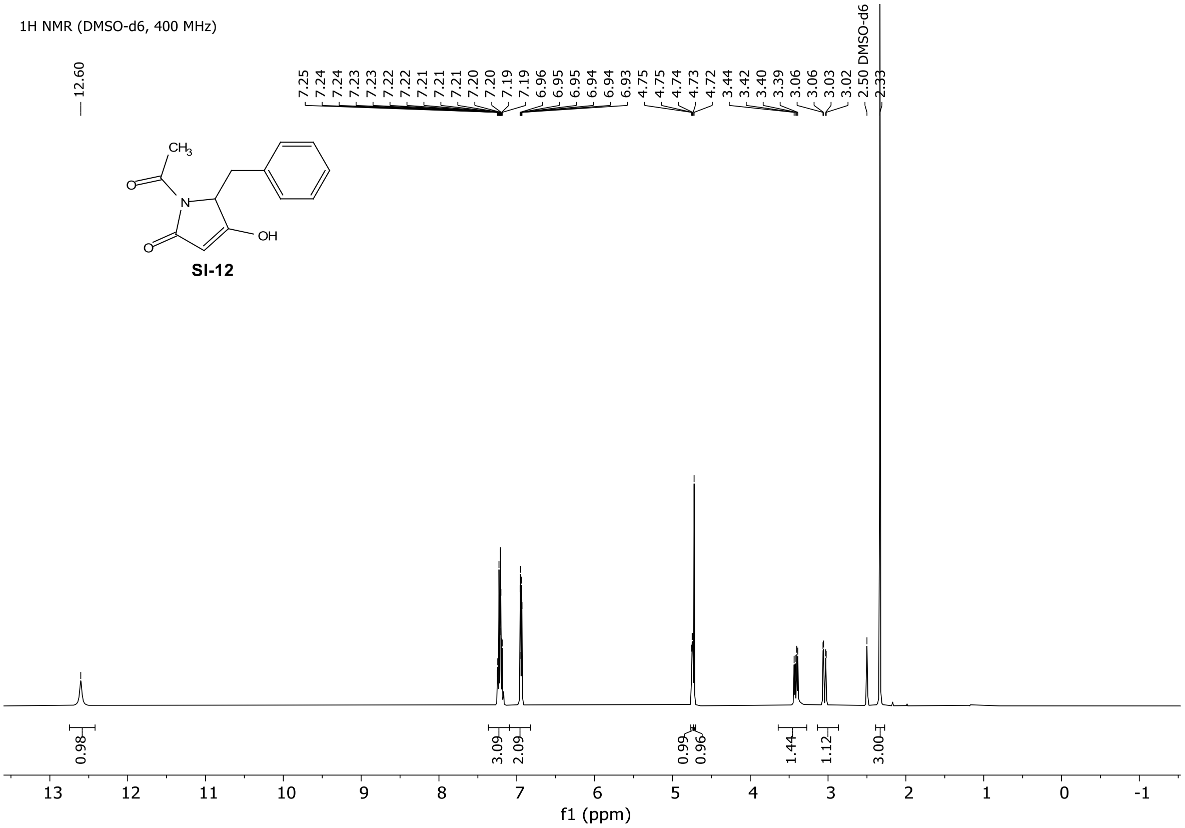

Figure S29: ^{1}H NMR of **SI-12**.

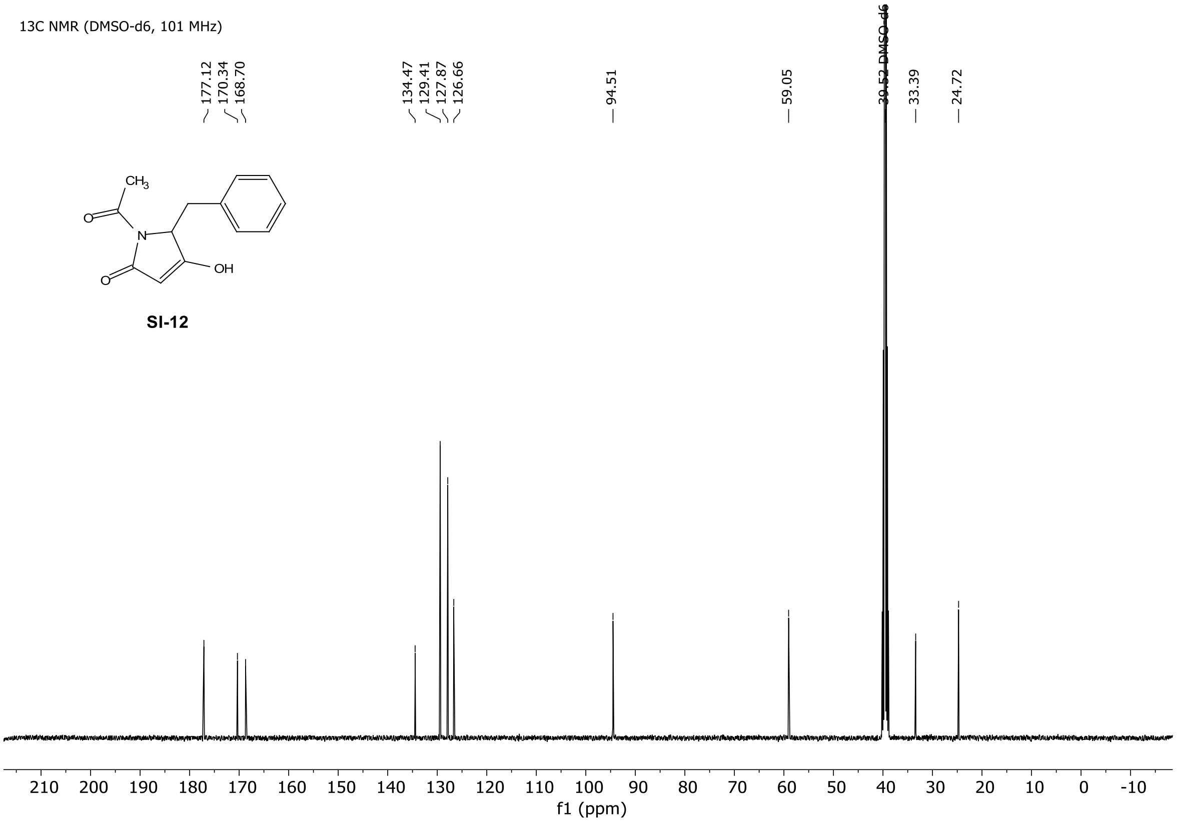

Figure S30: ^{13}C NMR of **SI-12**.

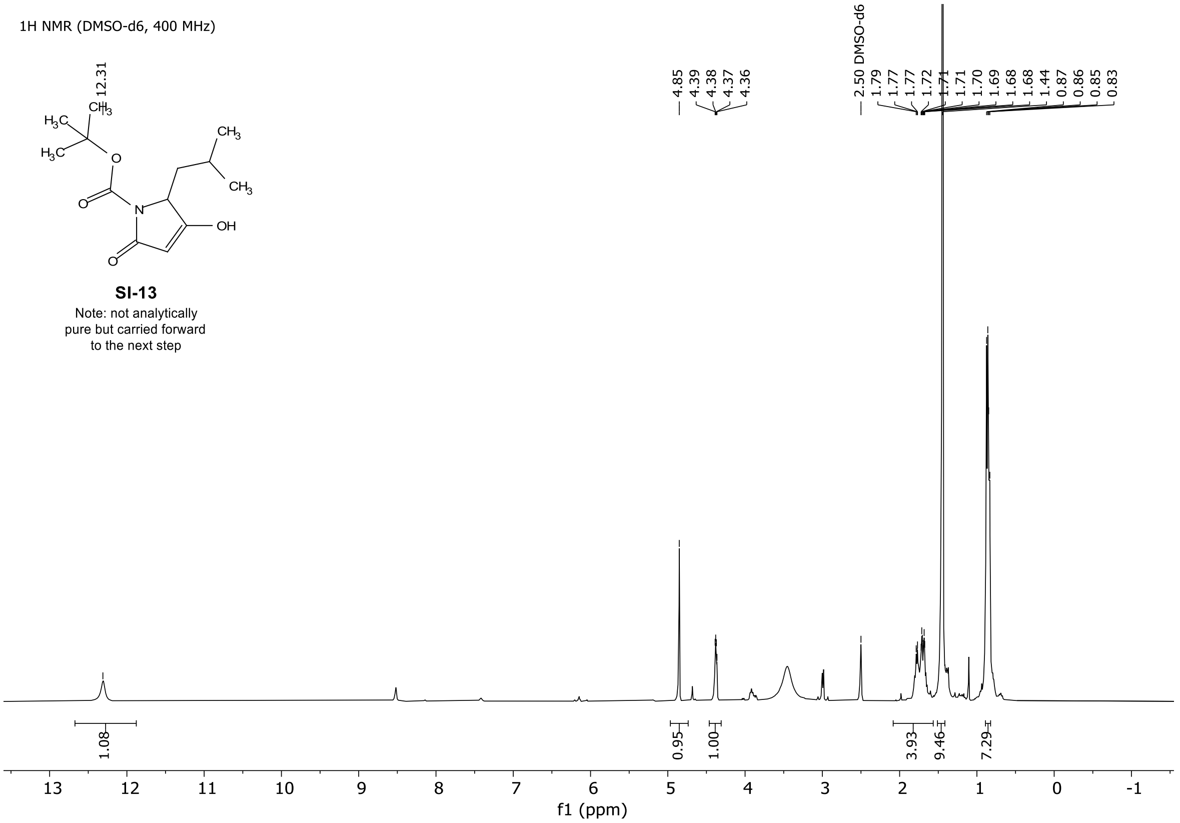

Figure S31: ^{1}H NMR of **SI-13**.

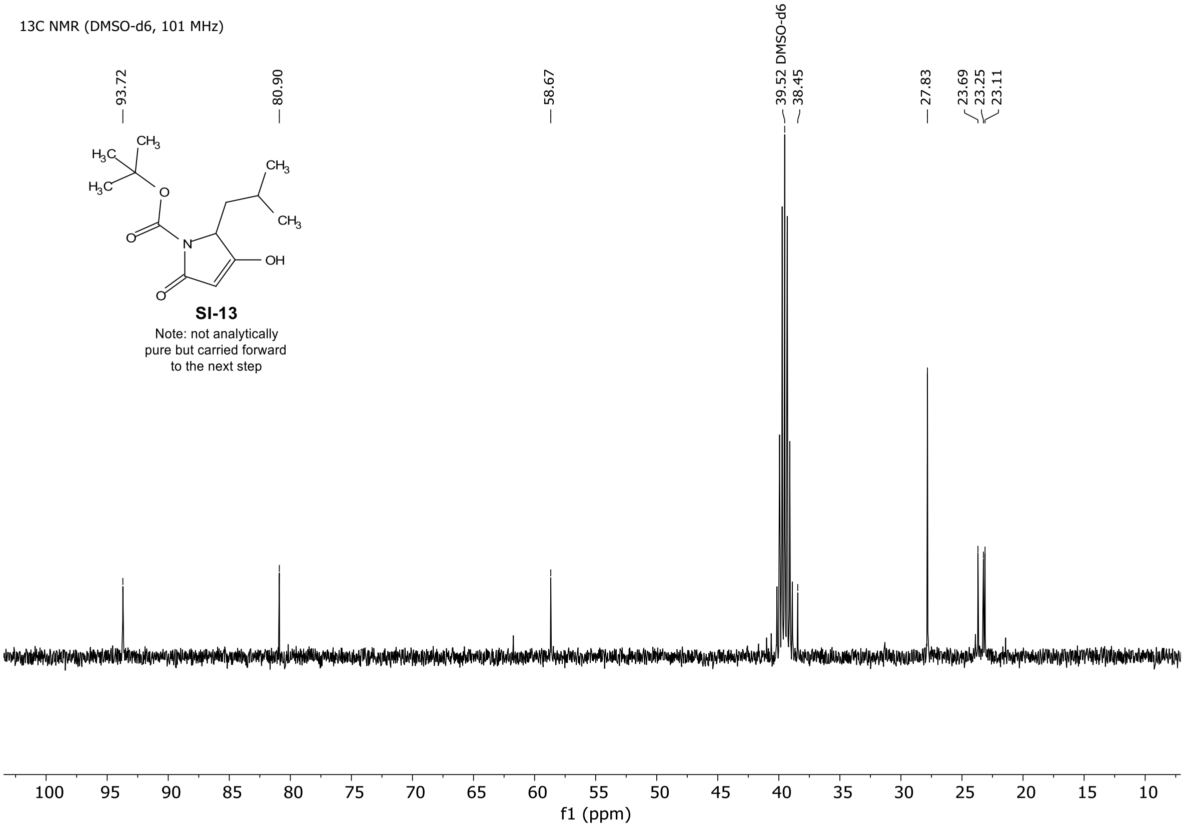

Figure S32: ^{13}C NMR of **SI-13**.

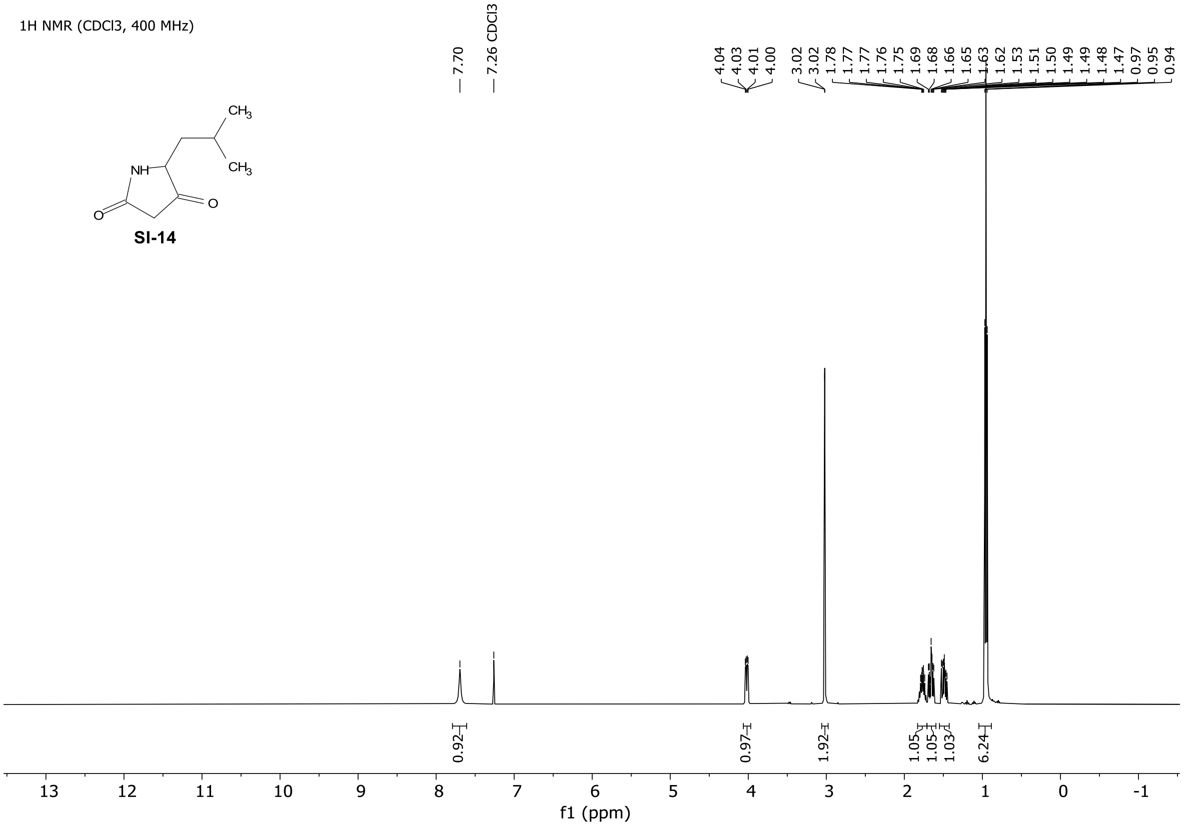

Figure S33: ^{1}H NMR of **SI-14**.

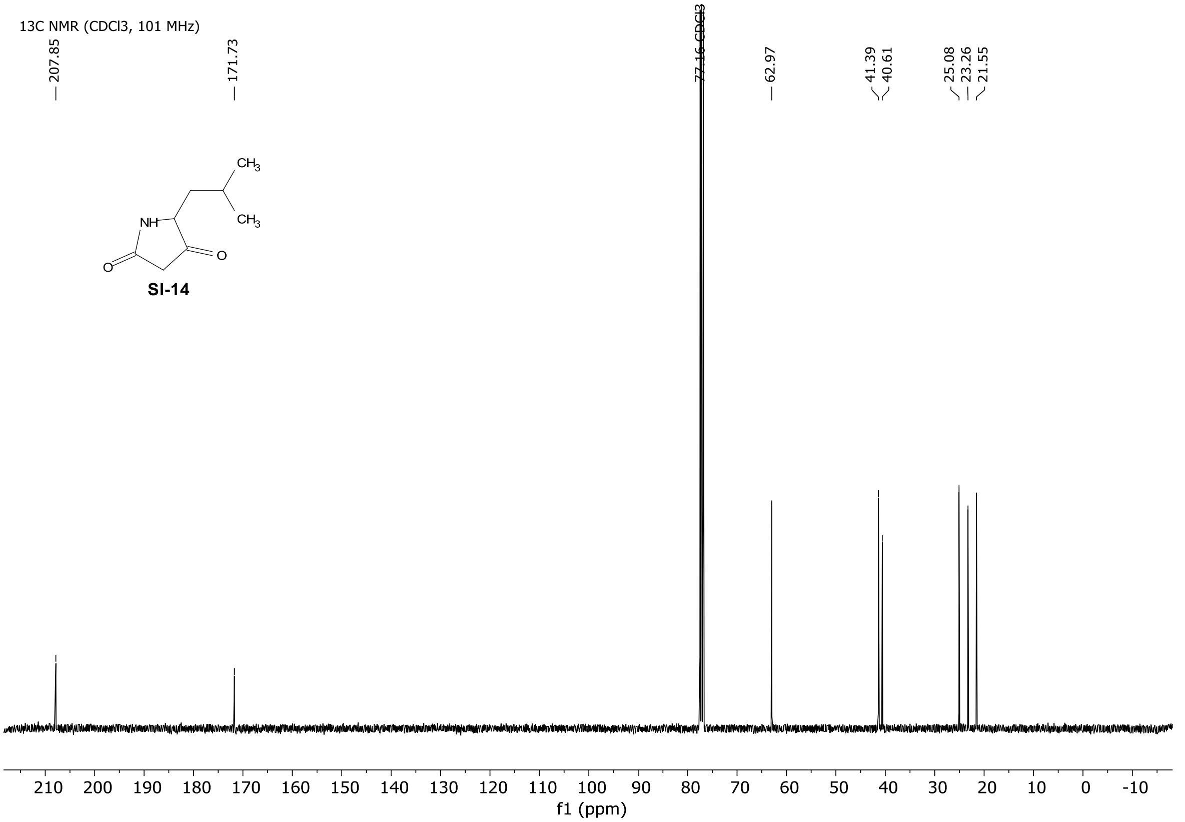

Figure S34: ^{13}C NMR of **SI-14**.

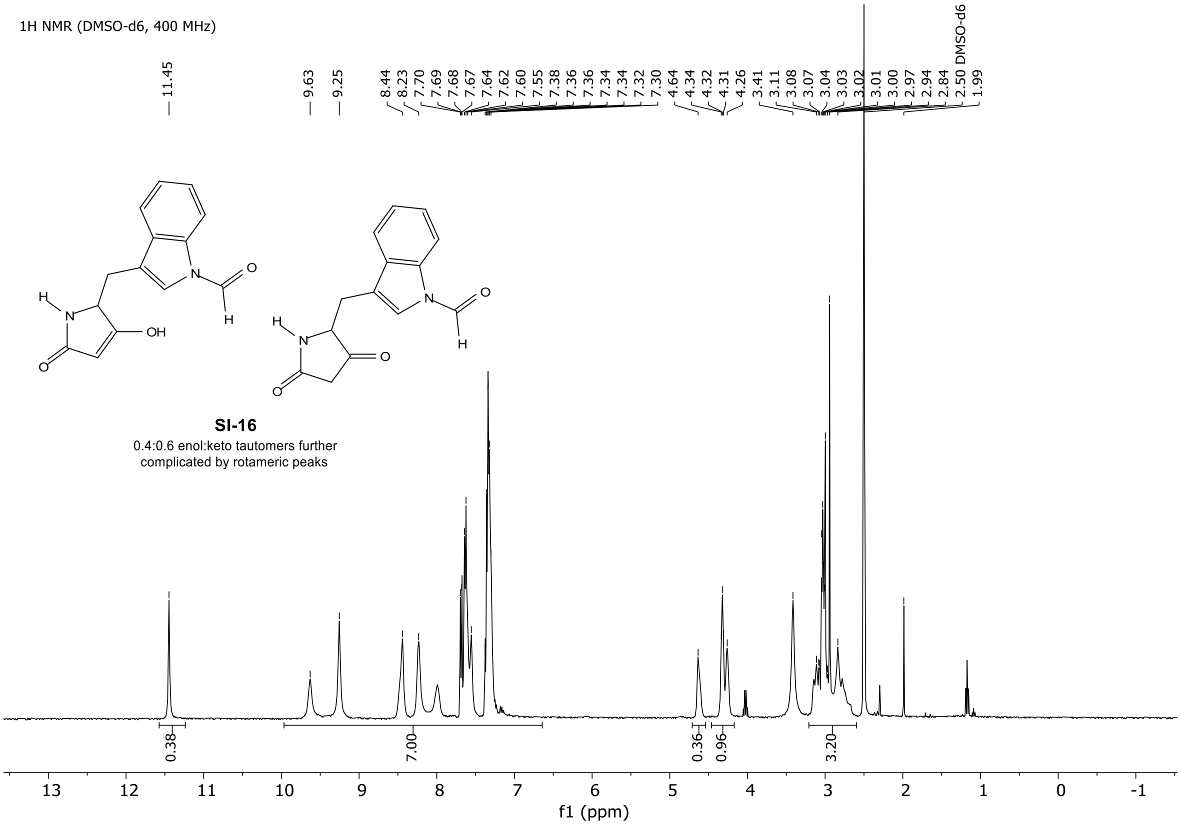

Figure S35: ^{1}H NMR of **SI-16**.

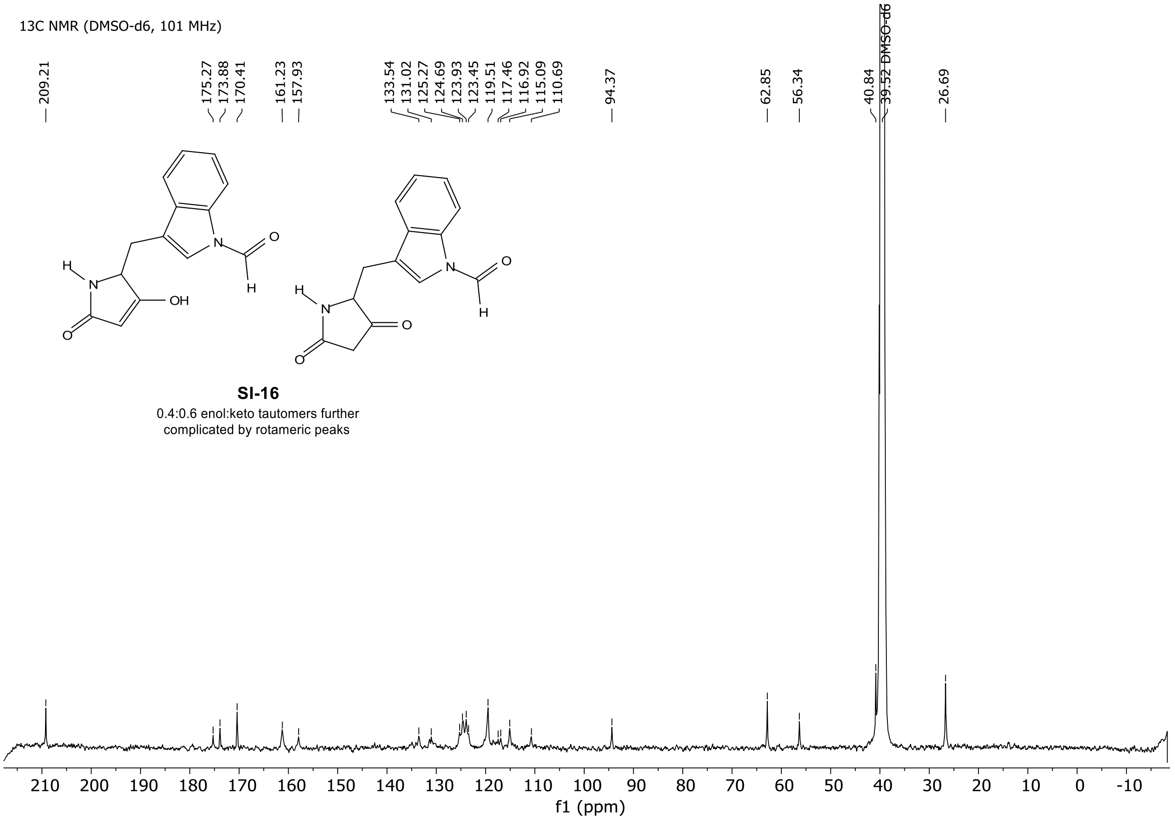

Figure S36: ^{13}C NMR of **SI-16**.

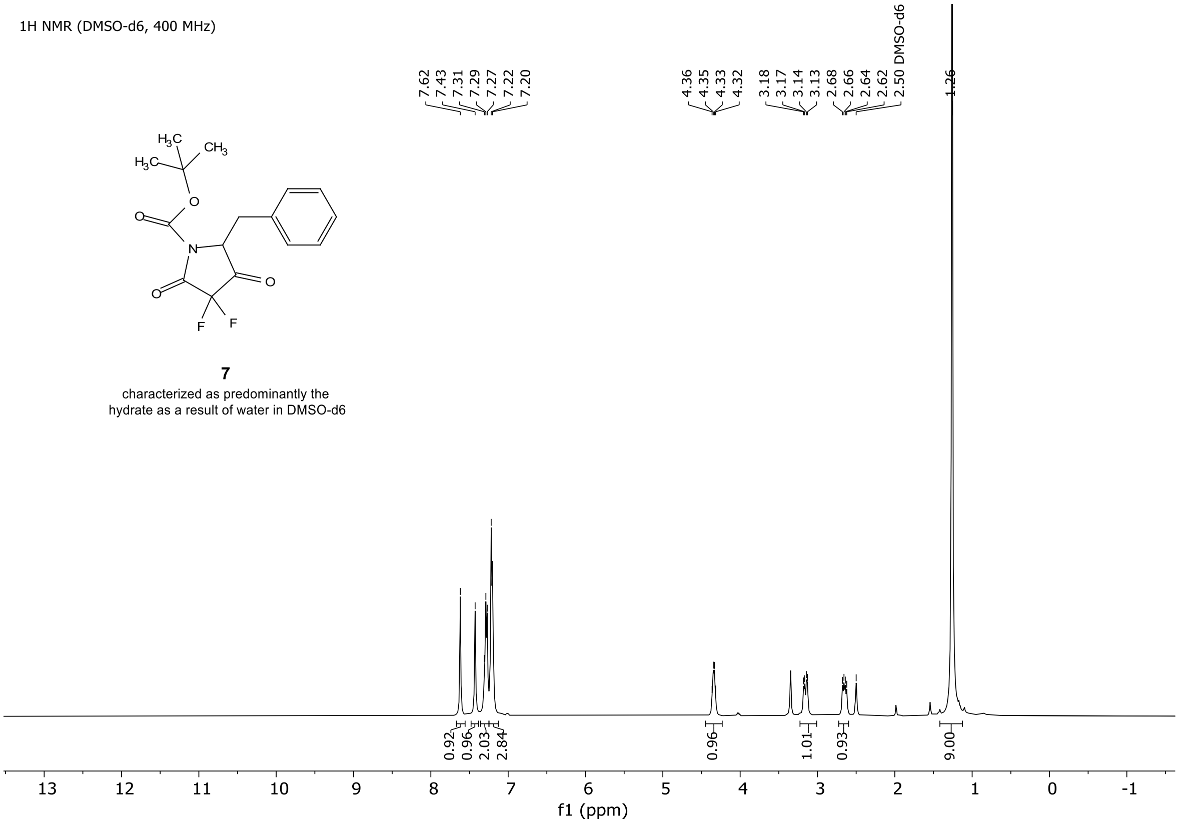

Figure S37: ^{1}H NMR for the characterization of **7**.

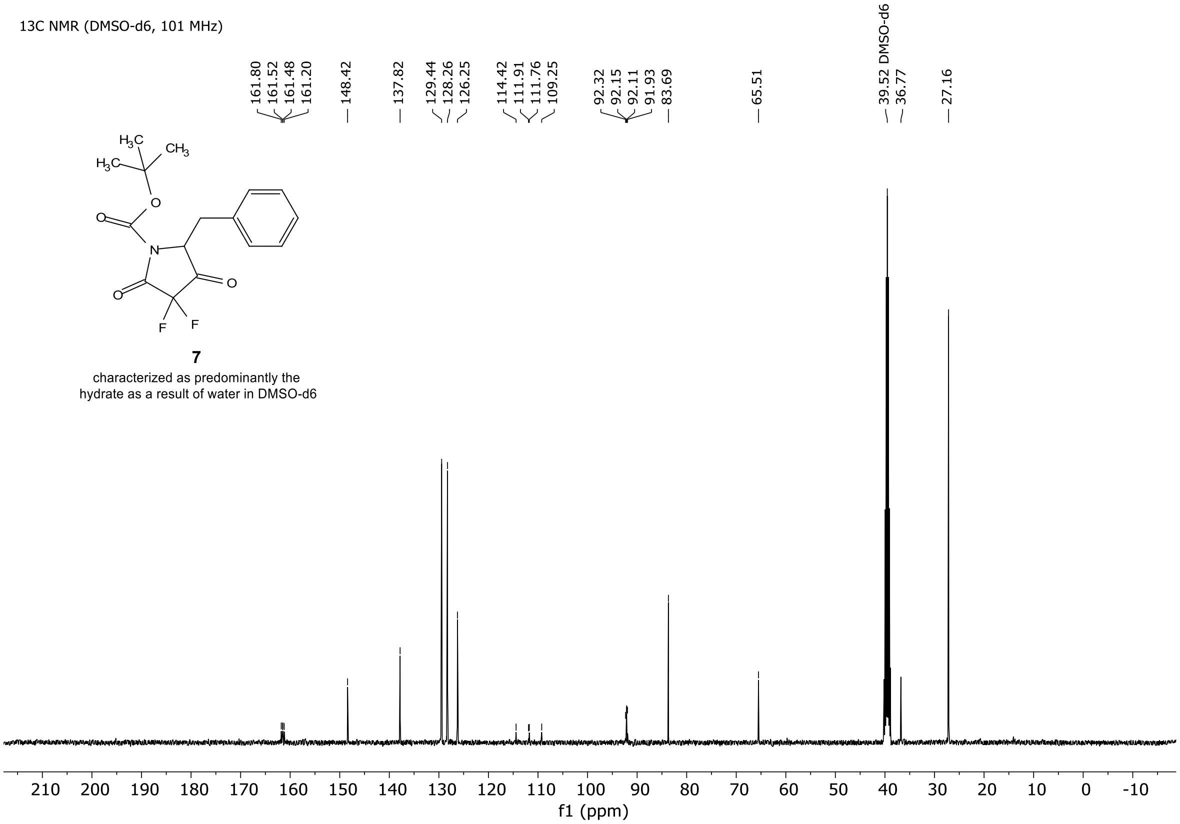

Figure S38: ^{13}C NMR for the characterization of **7**.

19F NMR (DMSO-d6, 376 MHz)

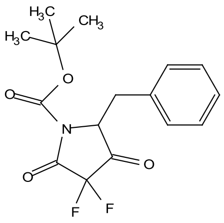

7

characterized as predominantly the hydrate as a result of water in DMSO-d6

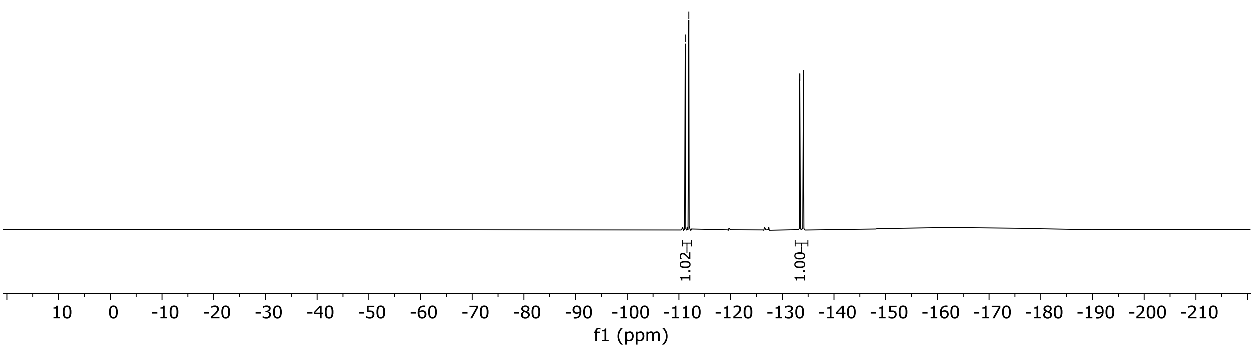

Figure S39: ^{19}F NMR for the characterization of **7**.

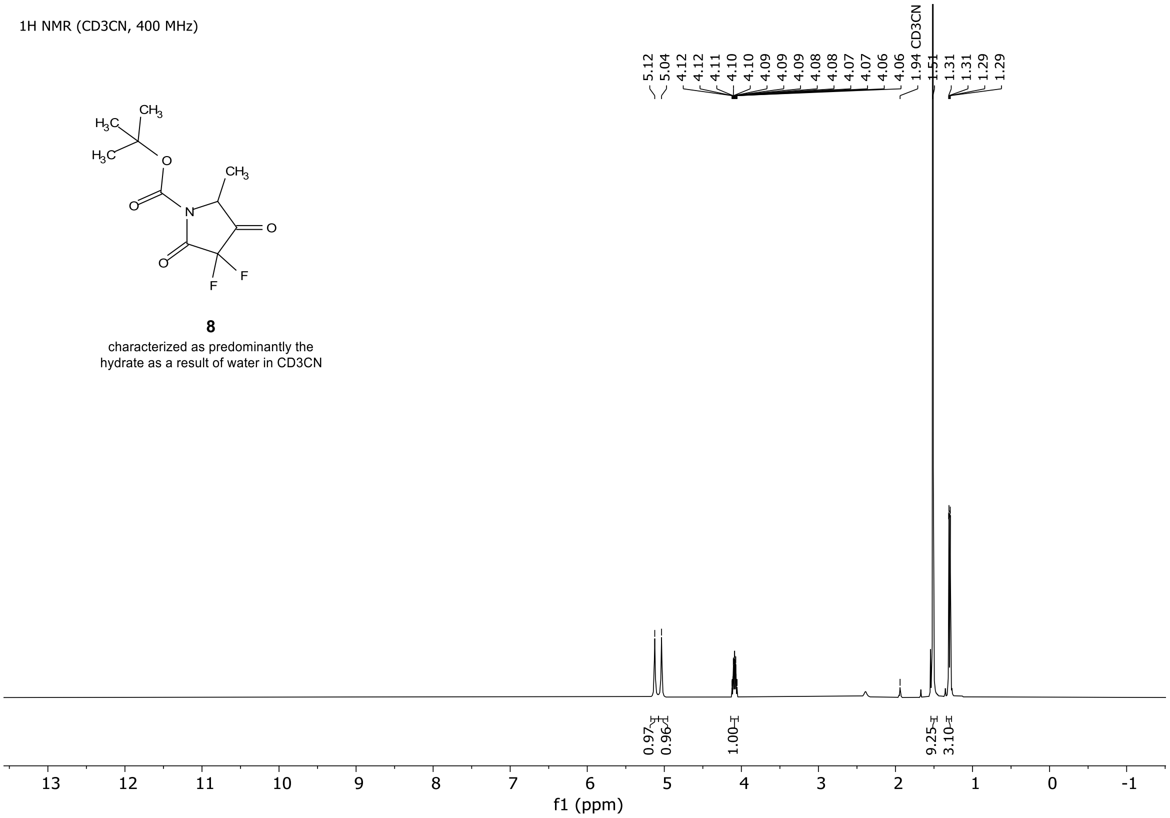

Figure S40: ^{1}H NMR for the characterization of **8**.

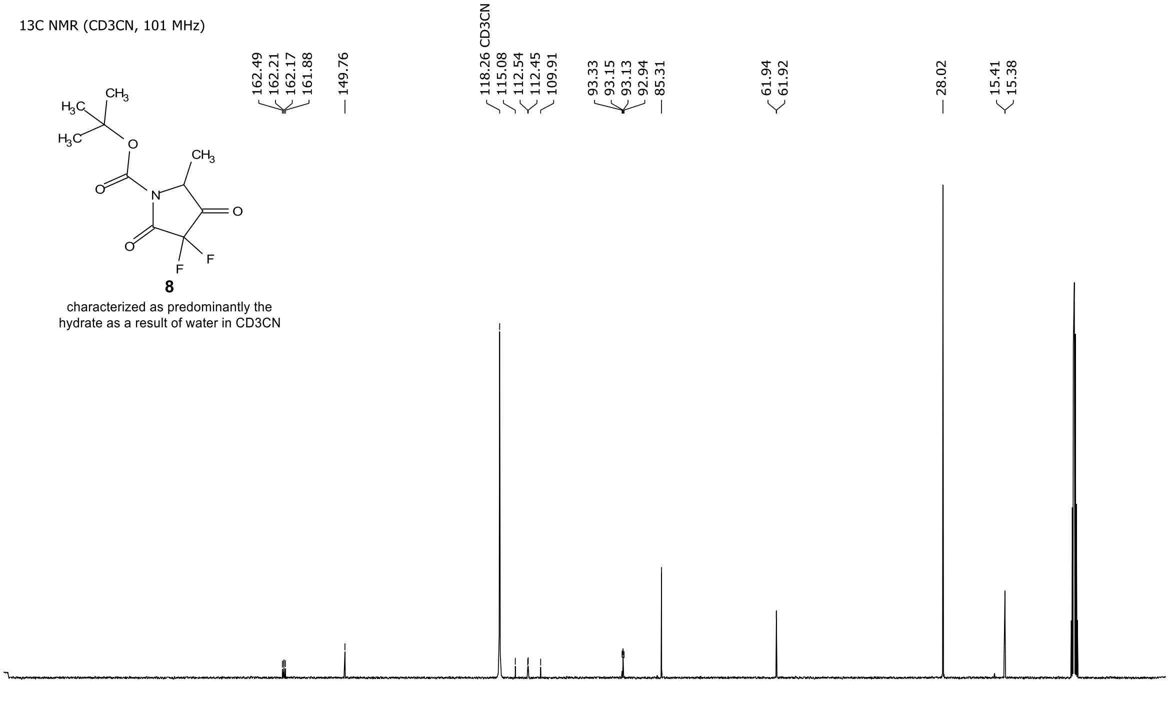

Figure S41: ^{13}C NMR for the characterization of **8**.

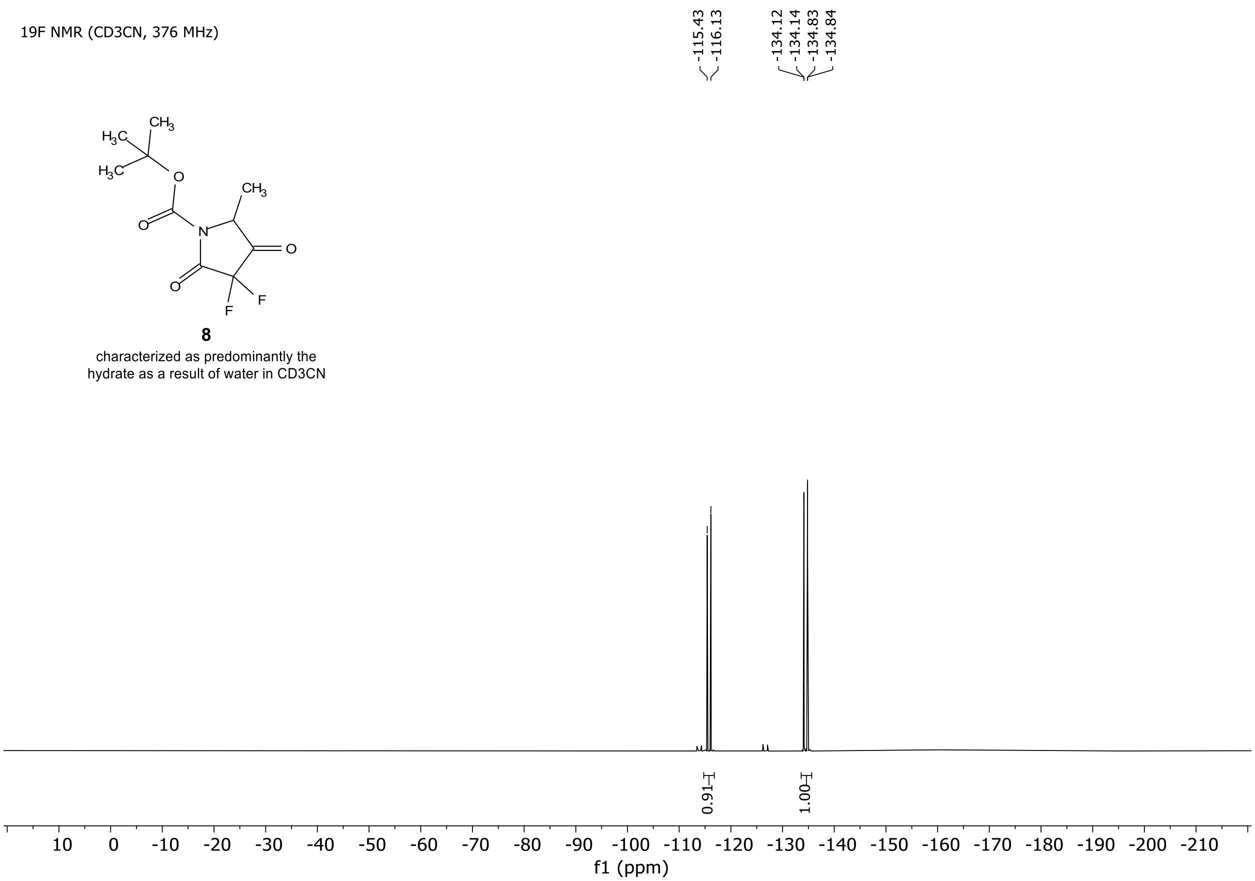

Figure S42: ^{19}F NMR for the characterization of **8**.

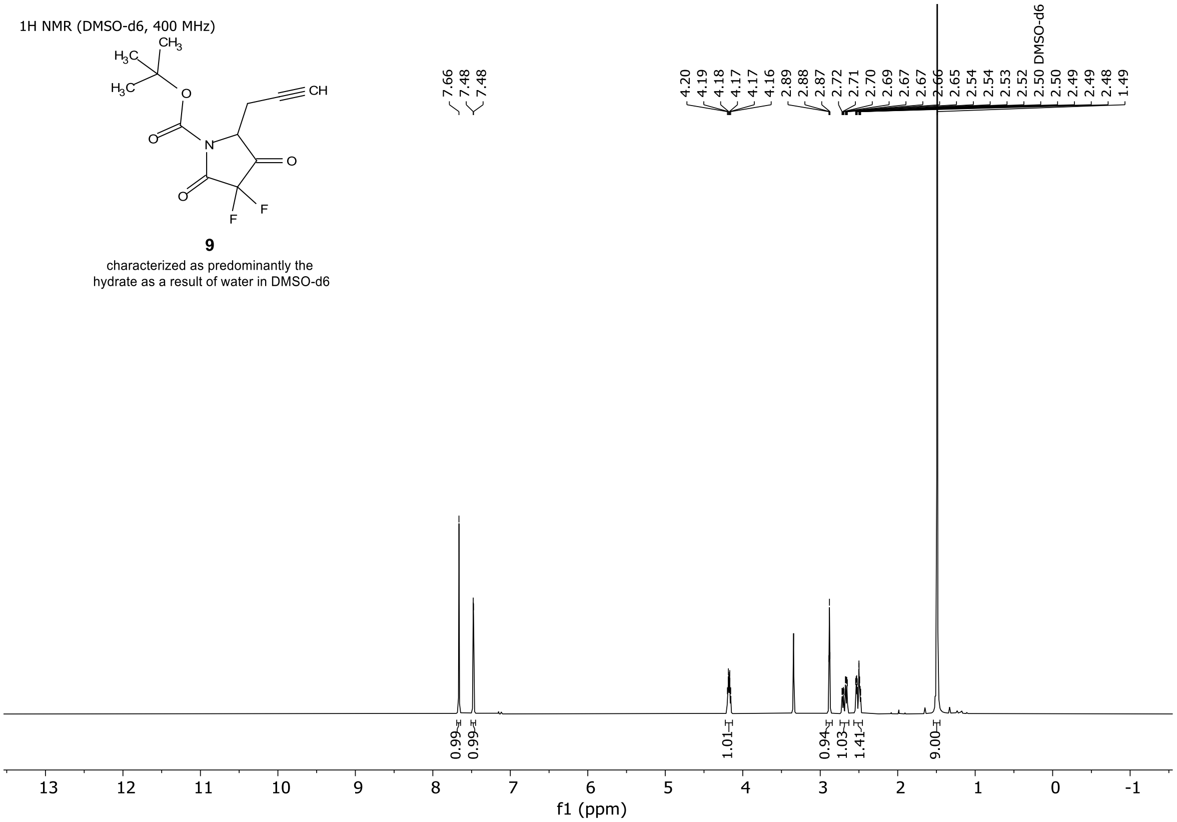

Figure S43: ^{1}H NMR for the characterization of **9**.

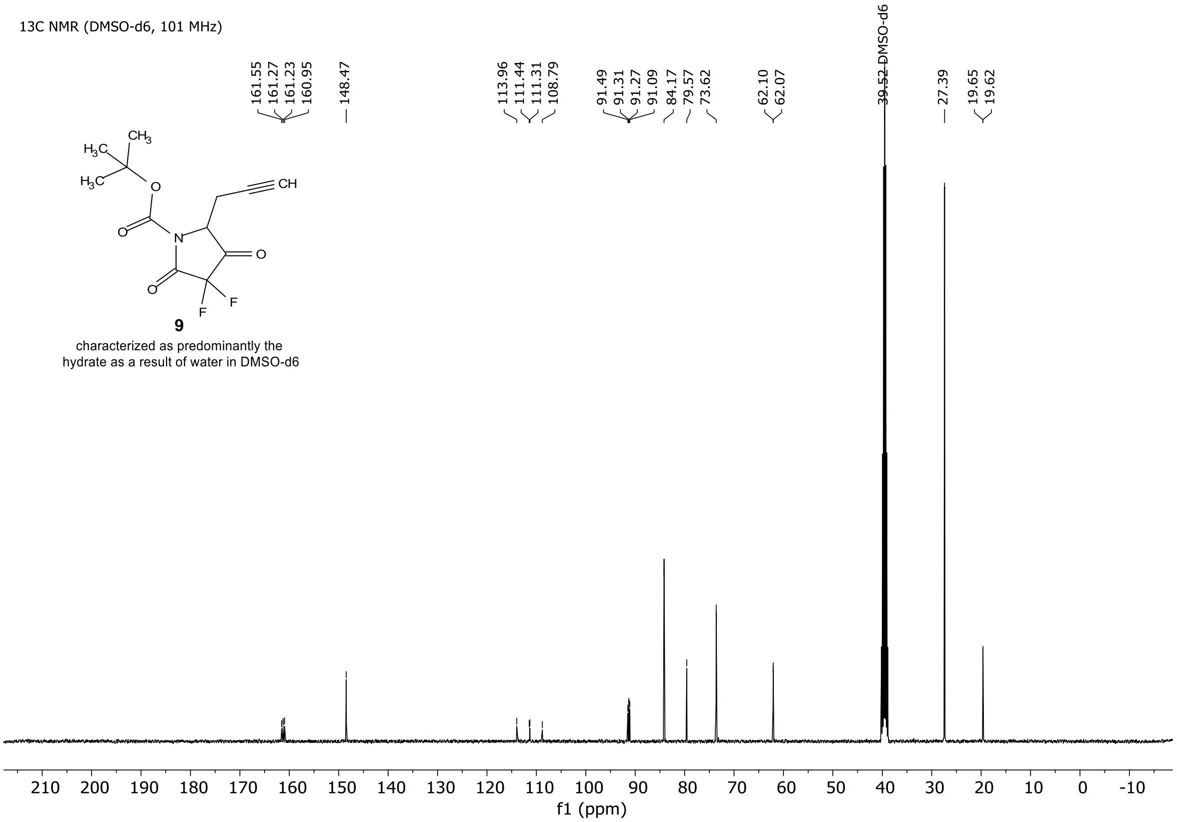

Figure S44: ^{13}C NMR for the characterization of **9**.

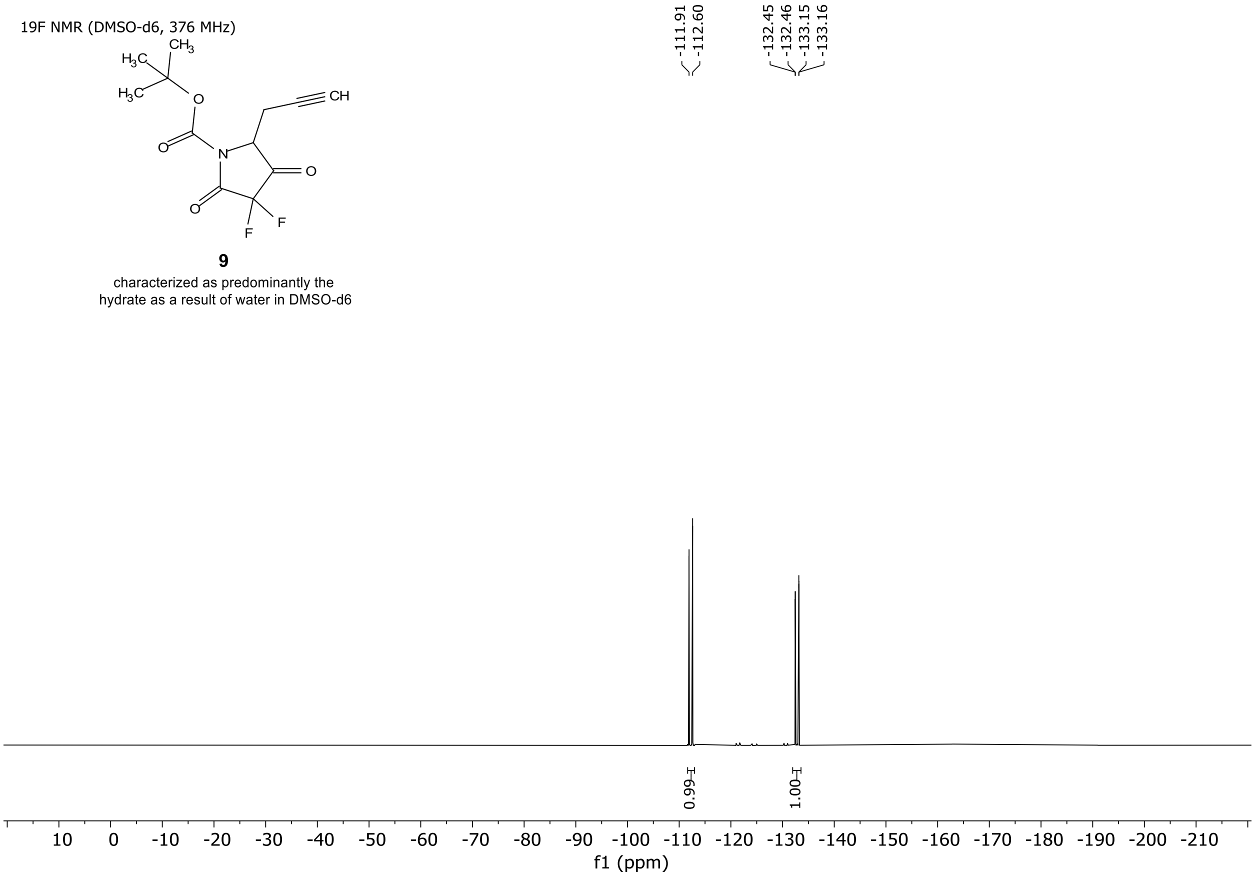

Figure S45: ^{19}F NMR for the characterization of **9**.

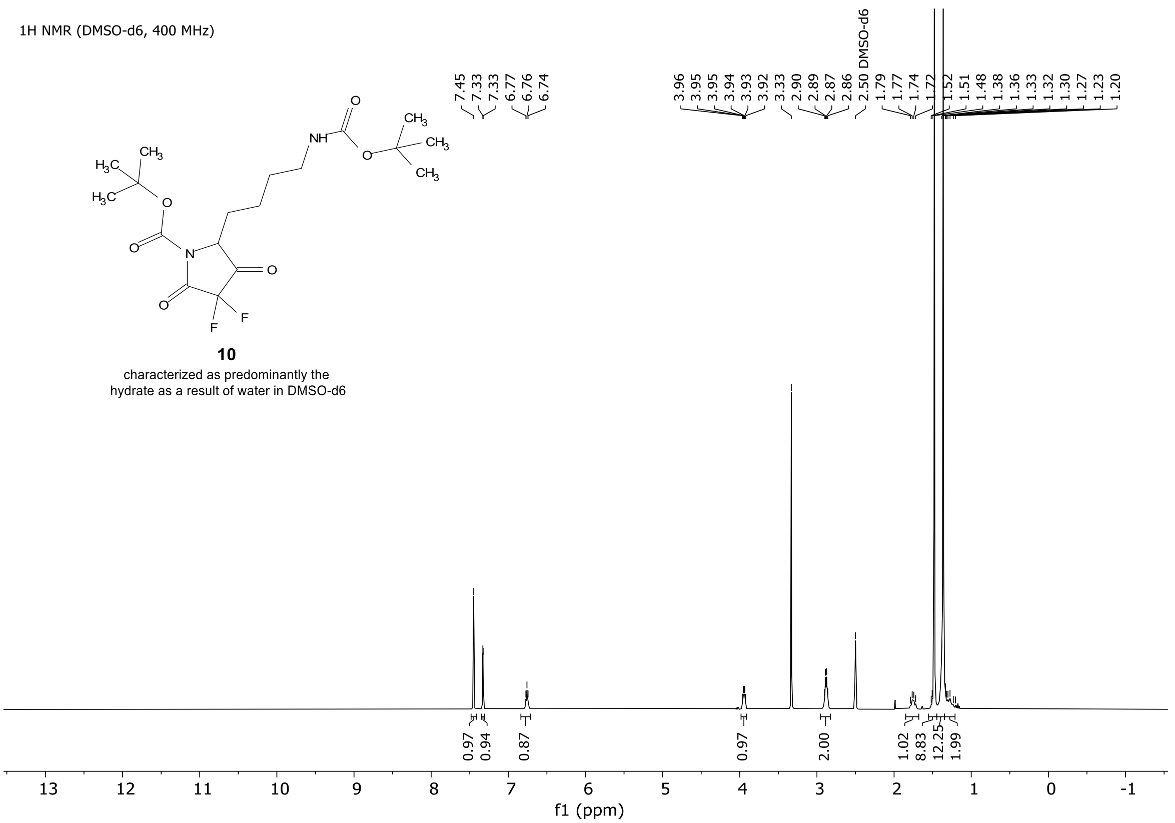

Figure S46: ^{1}H NMR for the characterization of **10**.

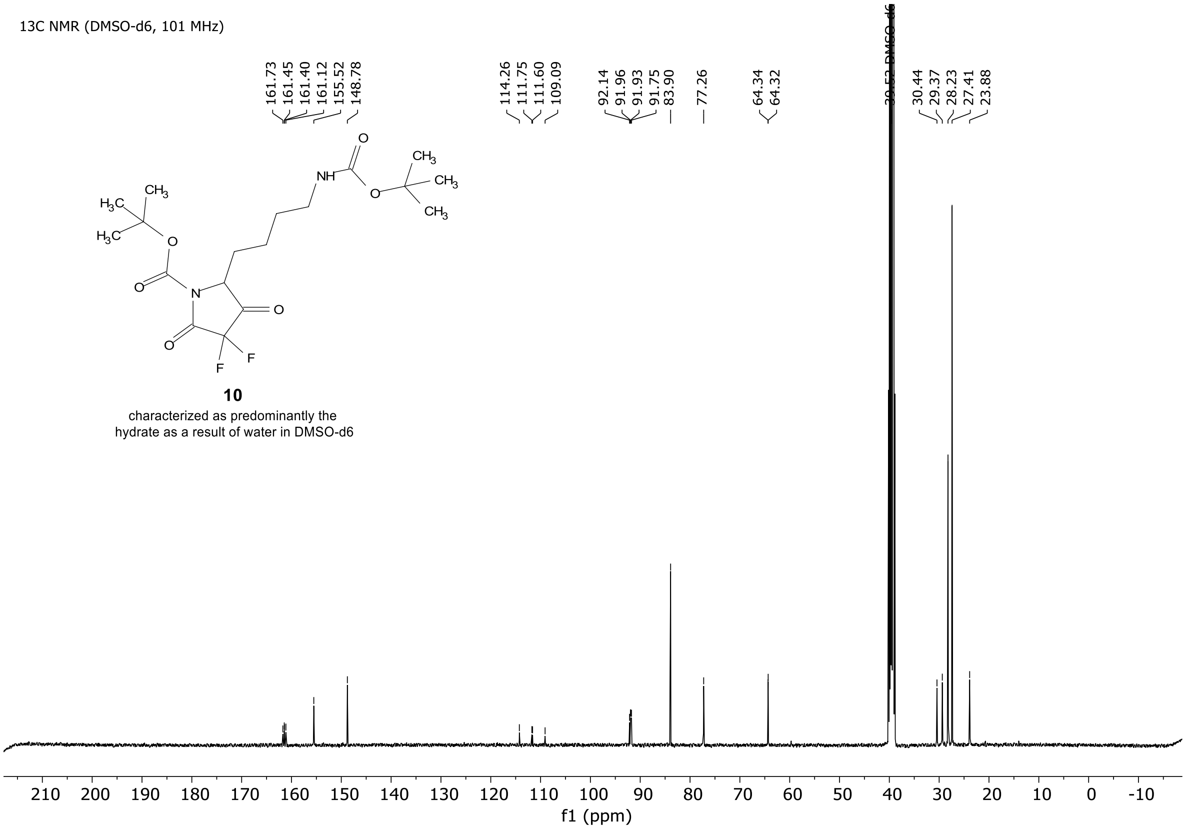

Figure S47: ^{13}C NMR for the characterization of **10**.

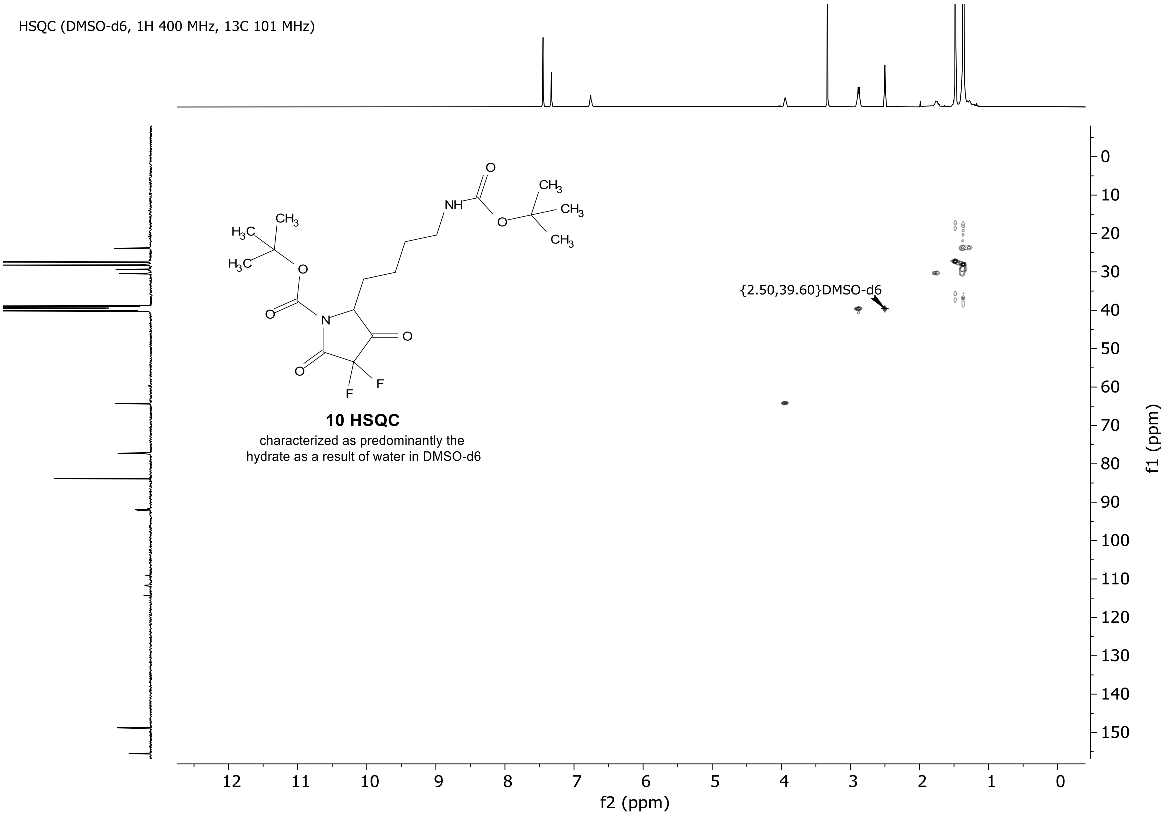

Figure S48: HSQC NMR for the characterization of **10**.

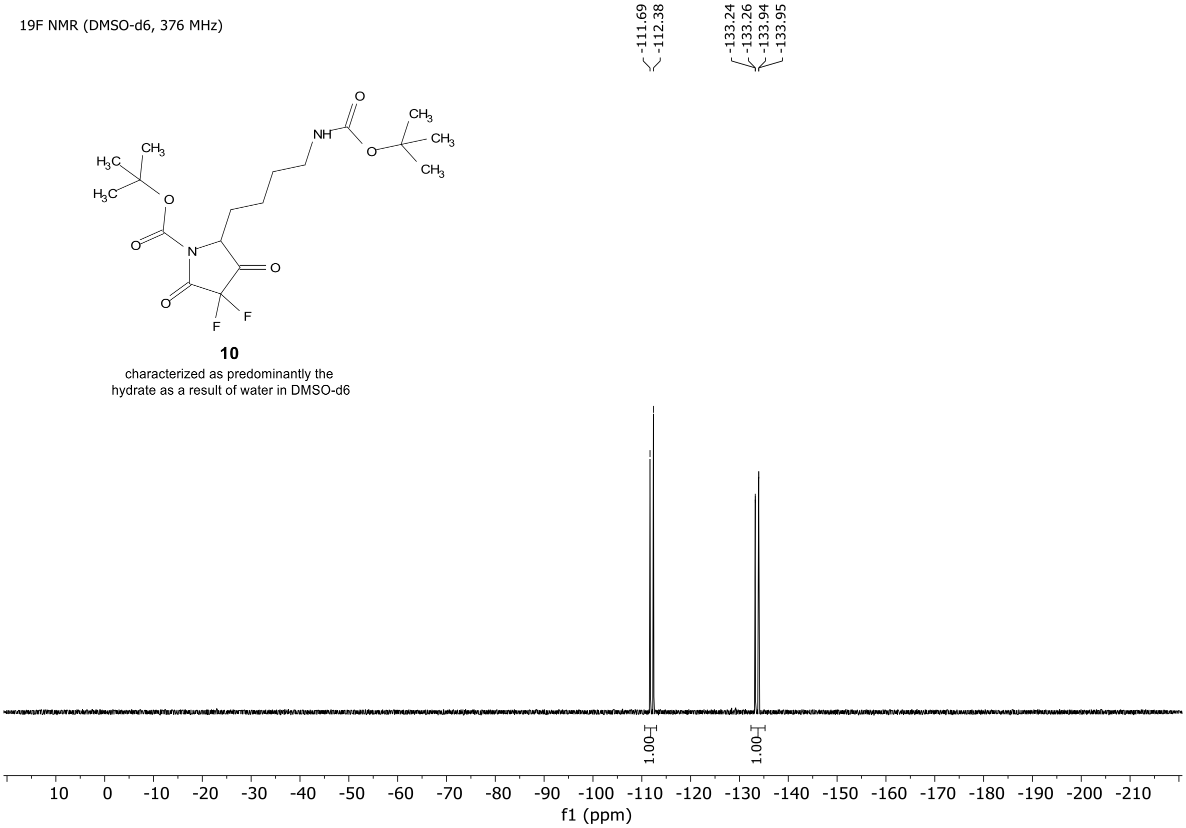

Figure S49: ^{19}F NMR for the characterization of **10**.

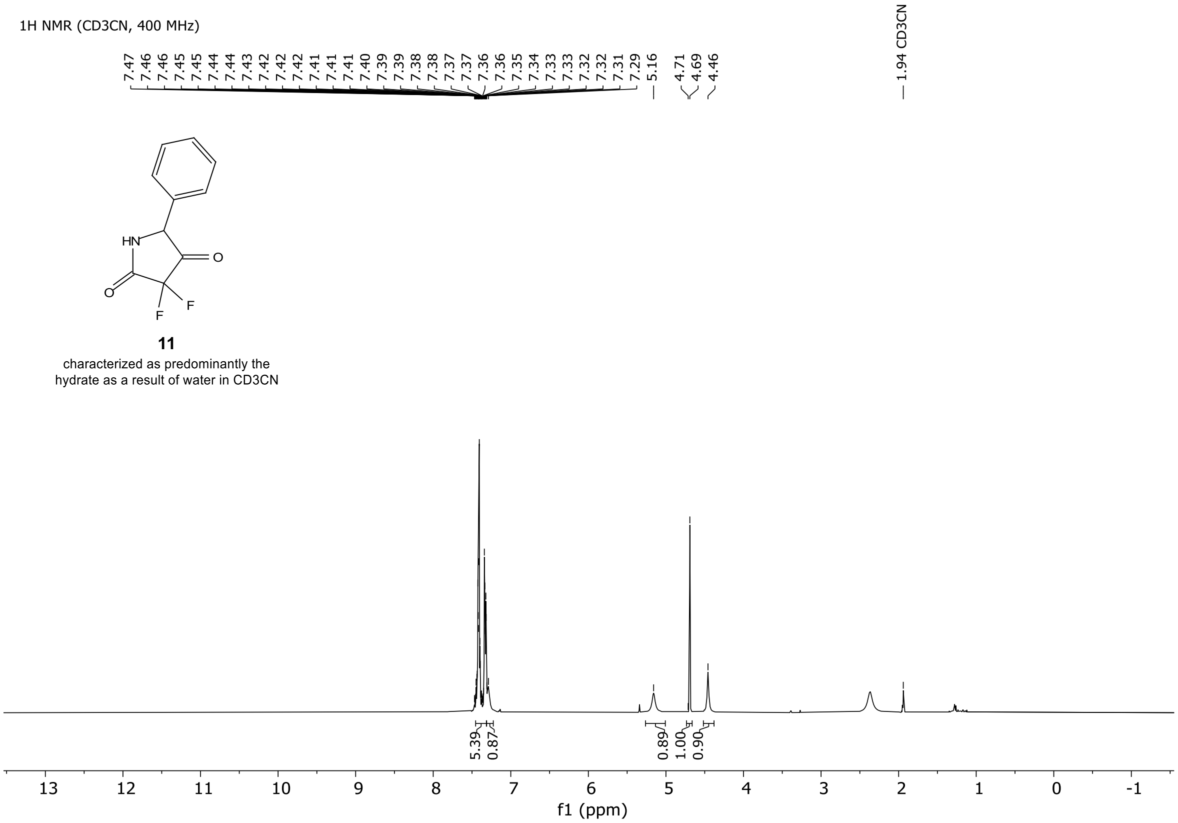

Figure S50: ^{1}H NMR for the characterization of **11**.

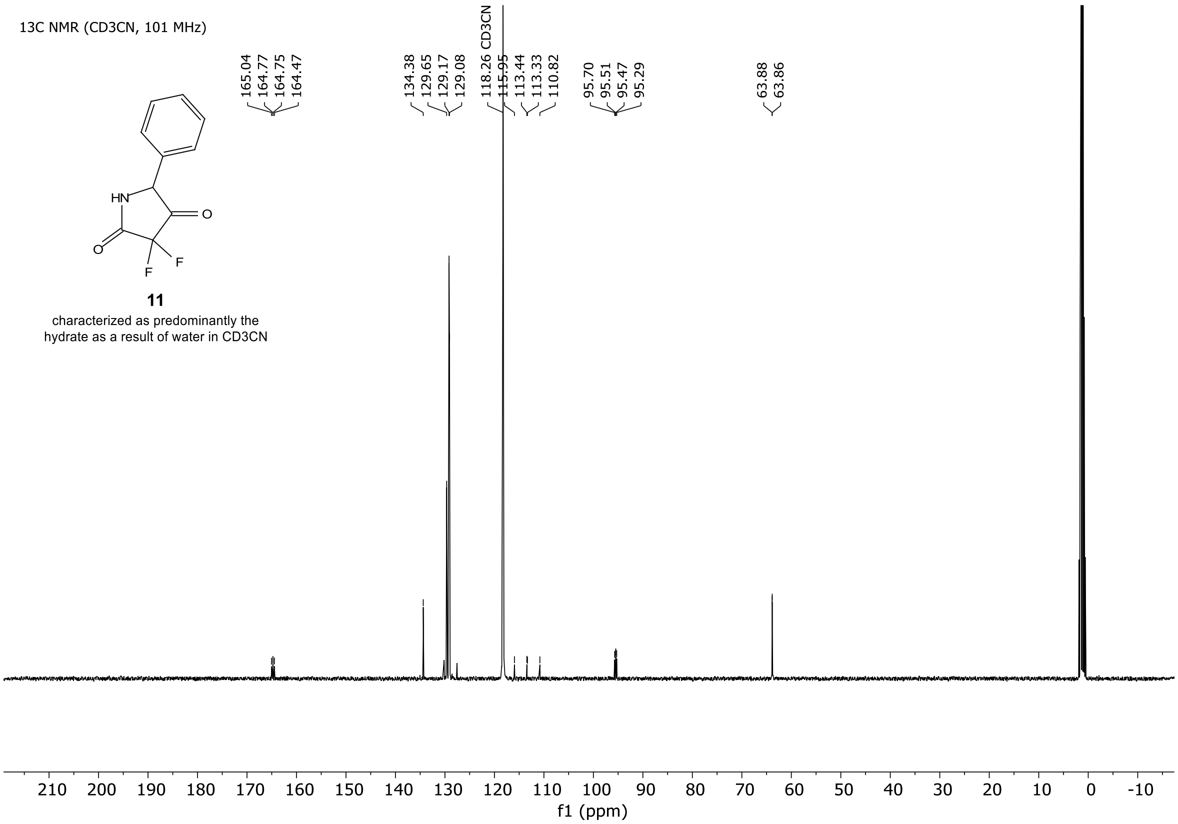

Figure S51: ^{13}C NMR for the characterization of **11**.

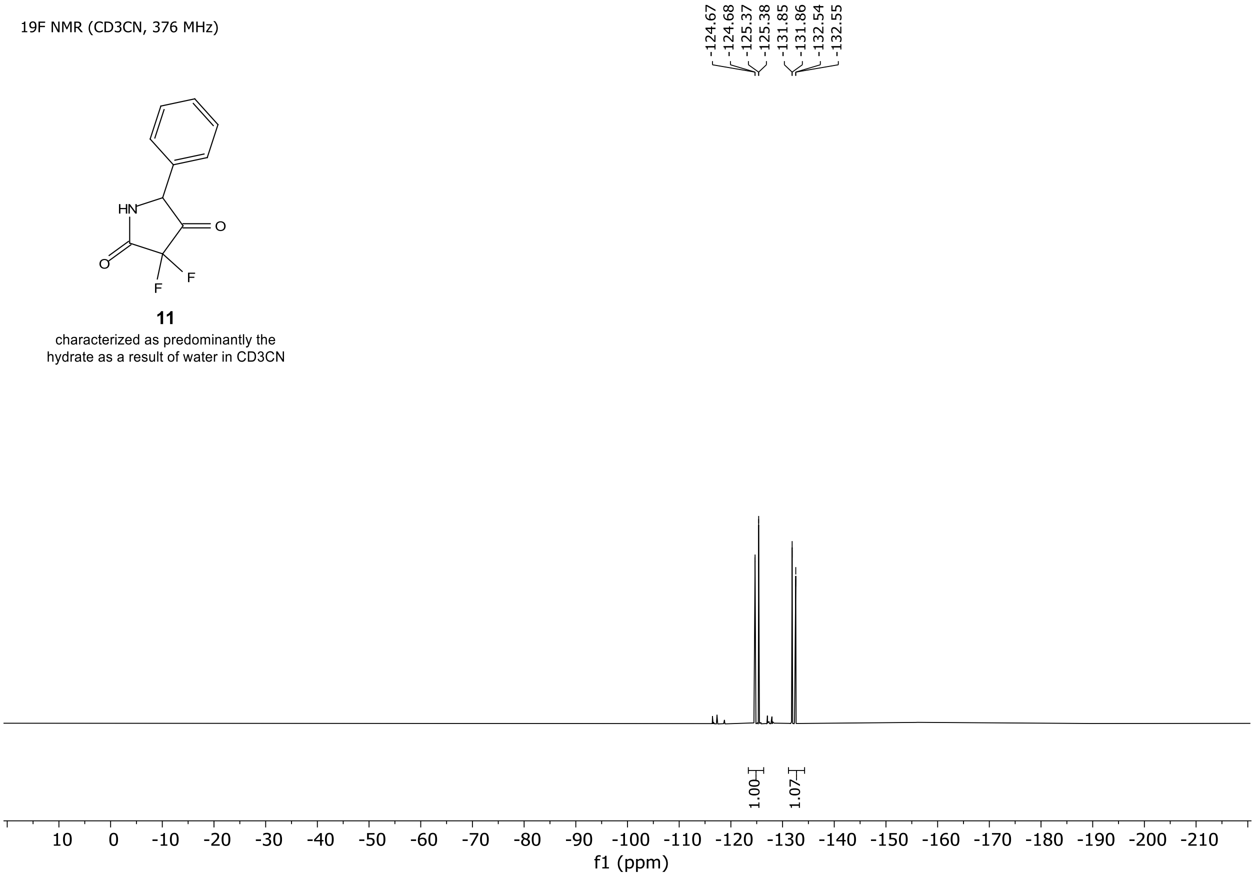

Figure S52: ^{19}F NMR for the characterization of **11**.

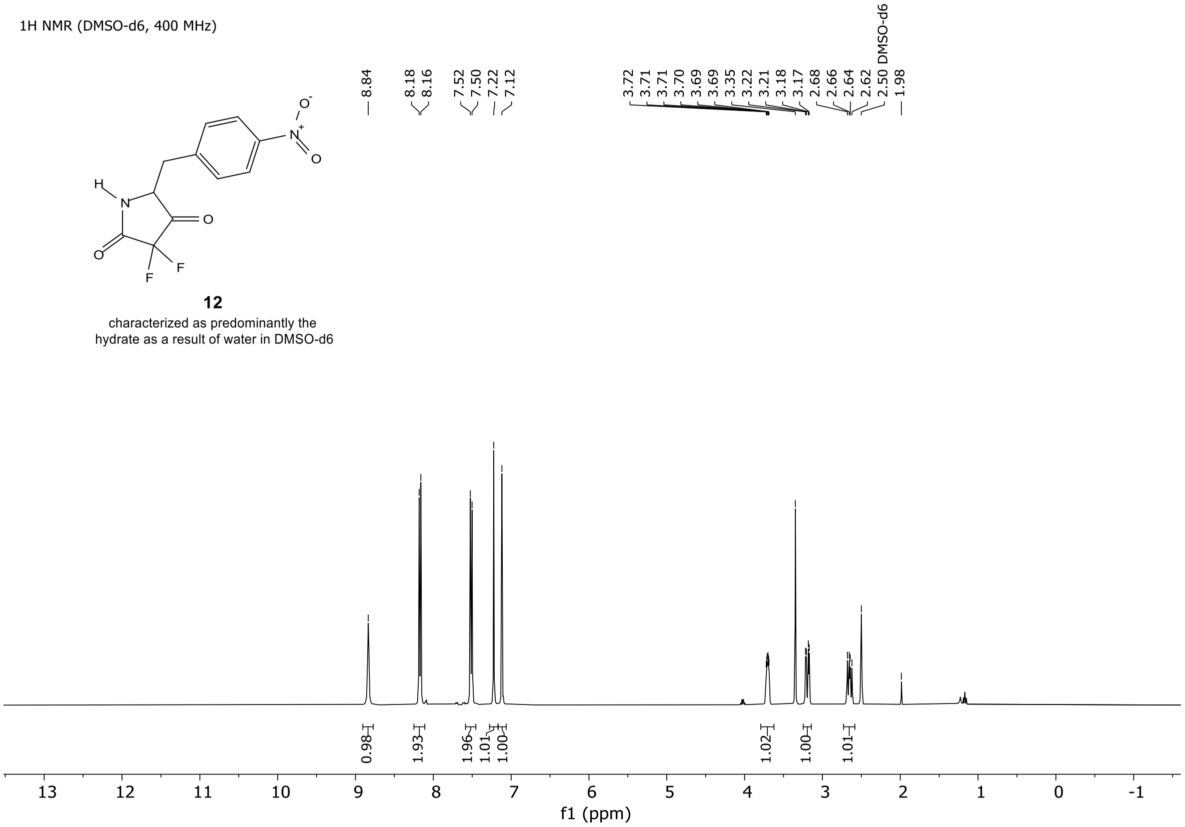

Figure S53: ^{1}H NMR for the characterization of **12**.

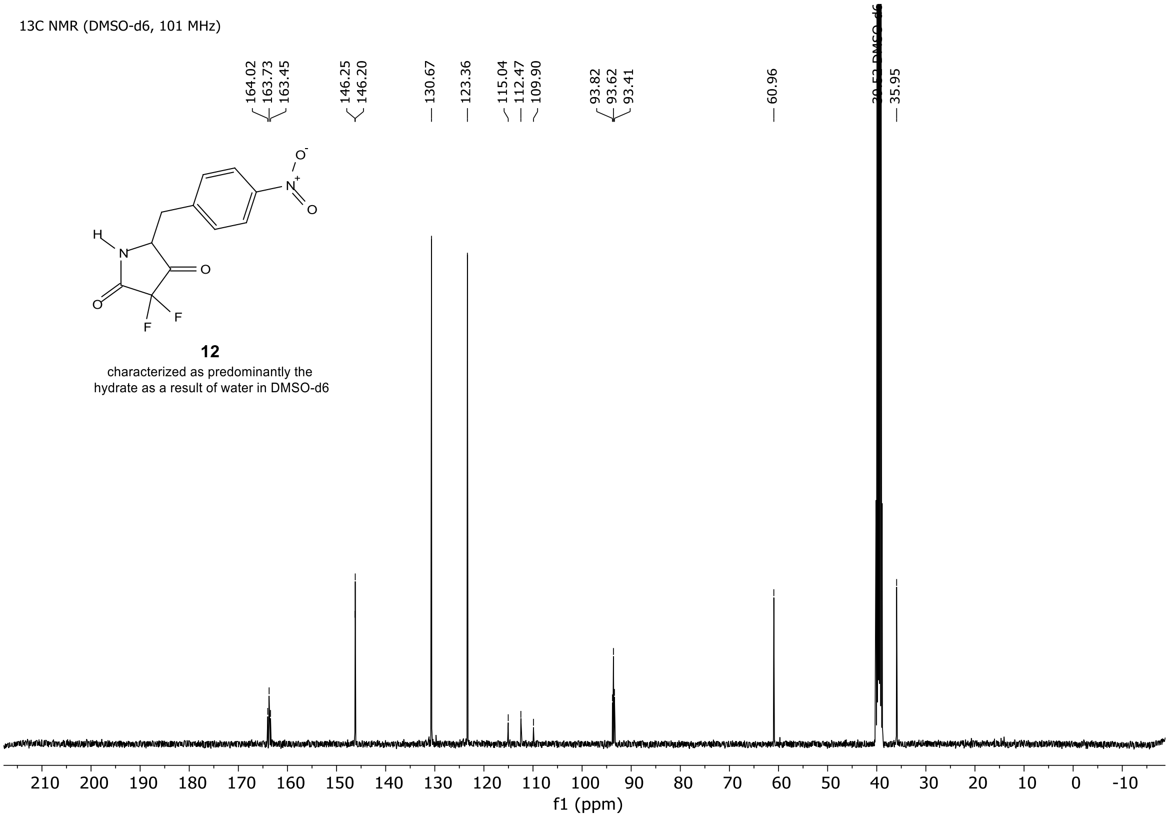

Figure S54: ^{13}C NMR for the characterization of **12**.

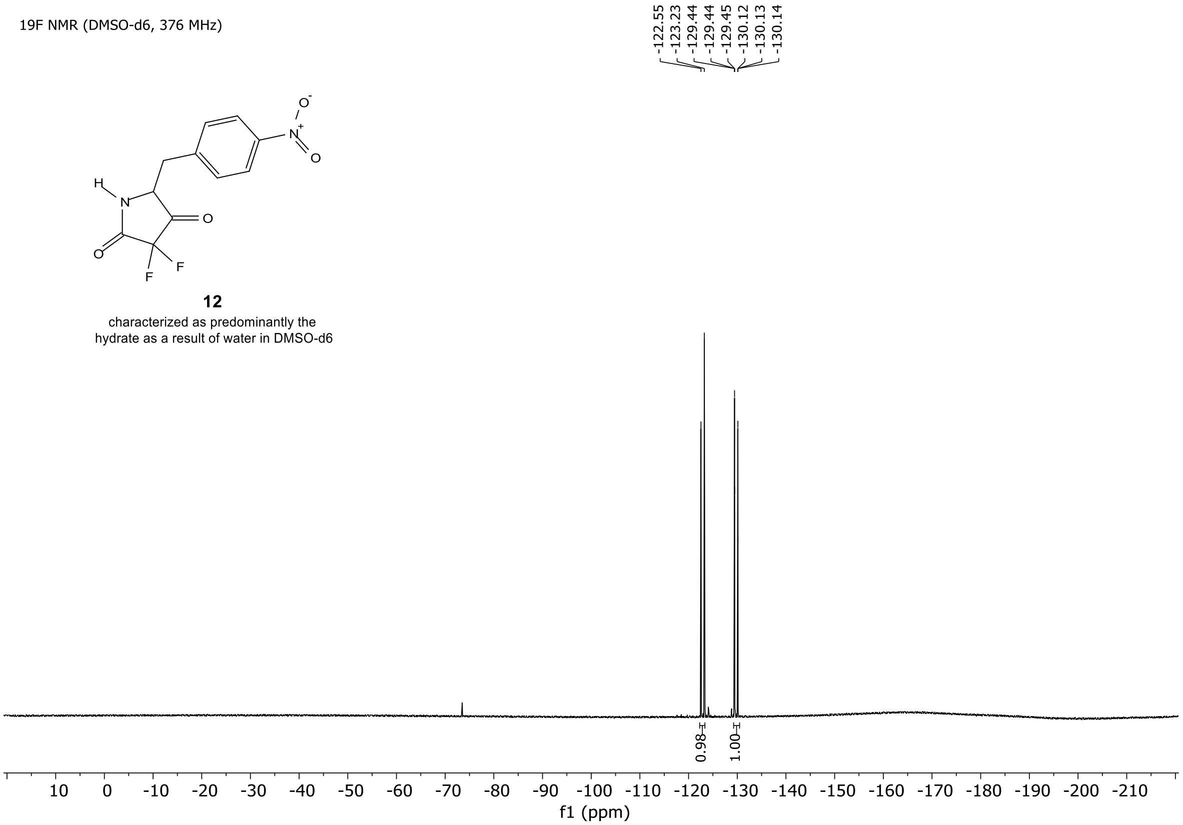

Figure S55: ^{19}F NMR for the characterization of **12**.

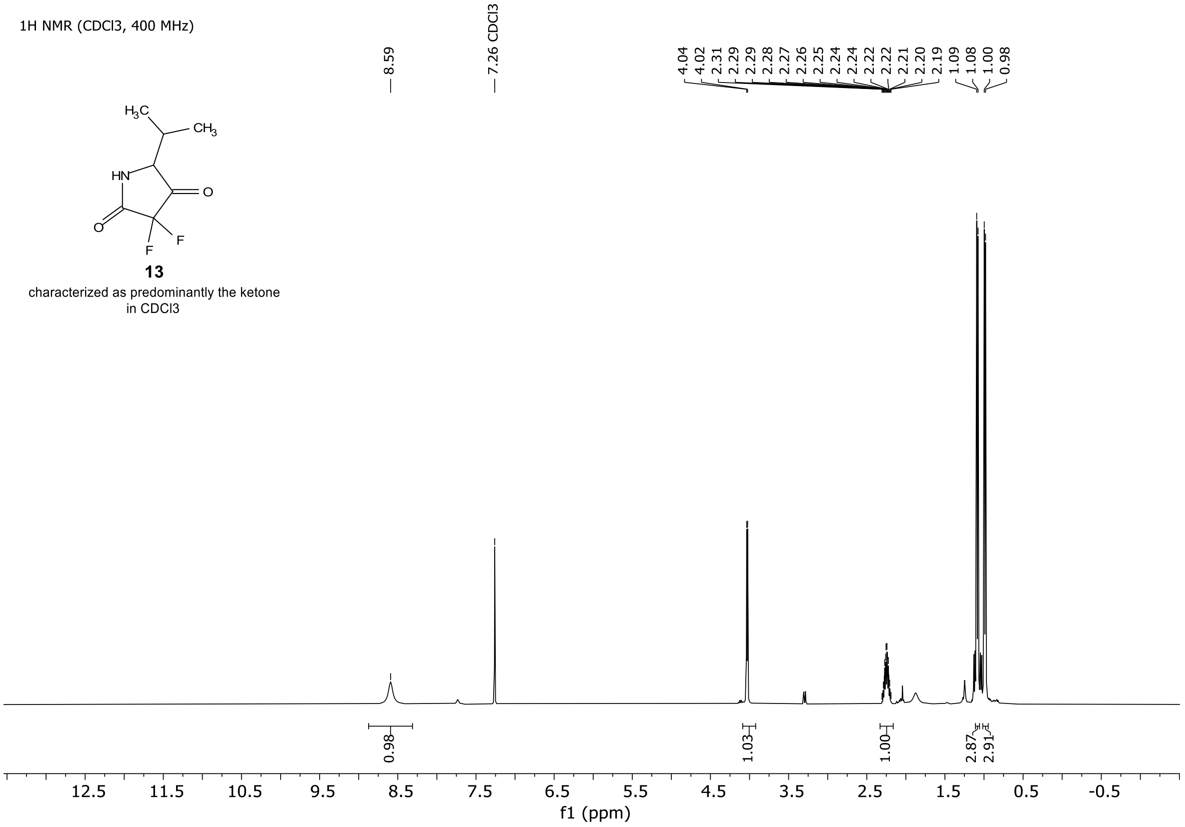

Figure S56: ^{1}H NMR for the characterization of **13**.

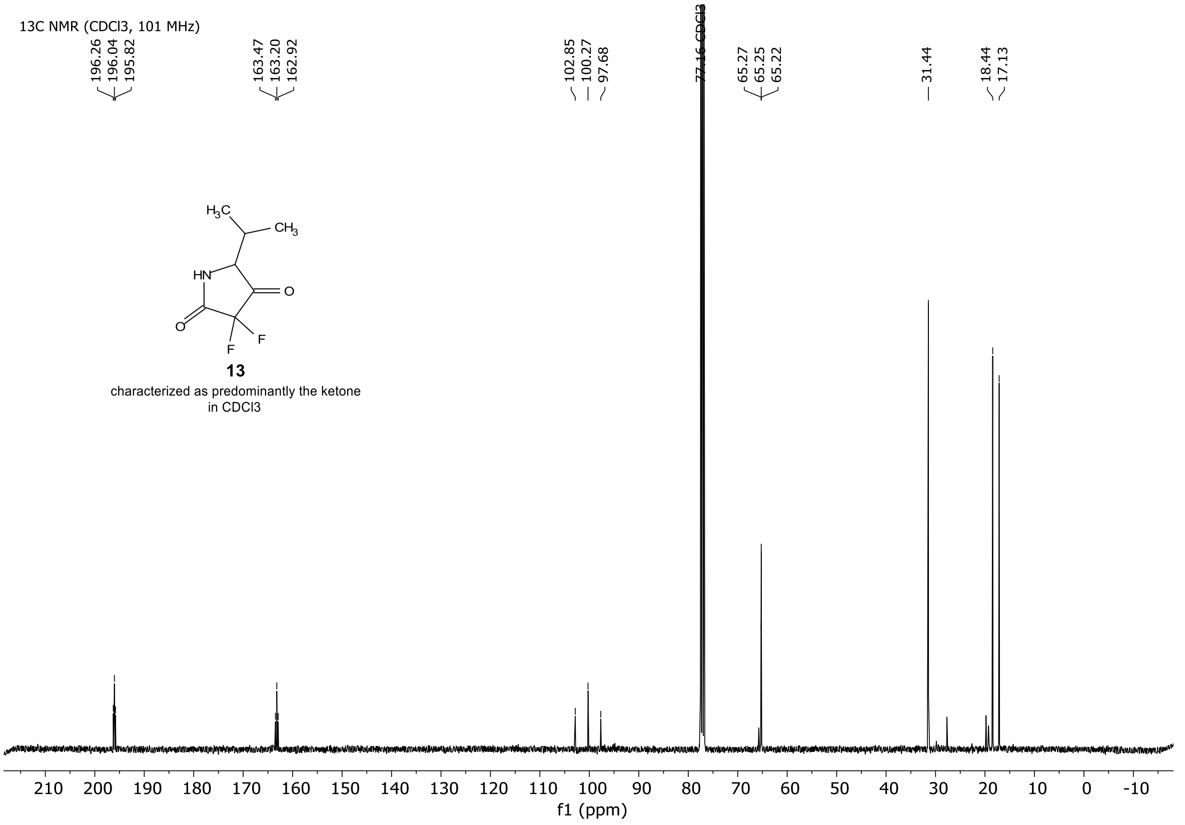

Figure S57: ^{13}C NMR for the characterization of **13**.

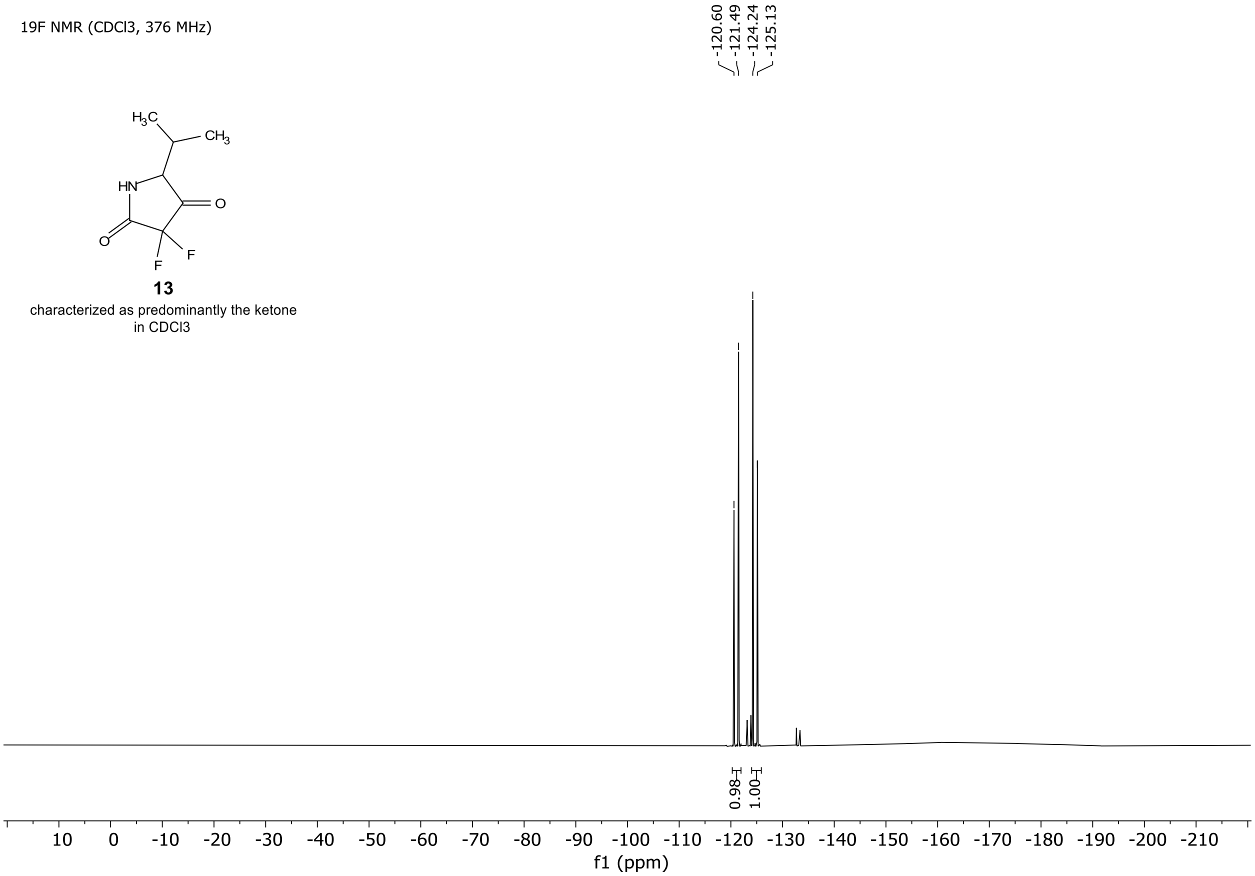

Figure S58: ^{19}F NMR for the characterization of **13**.

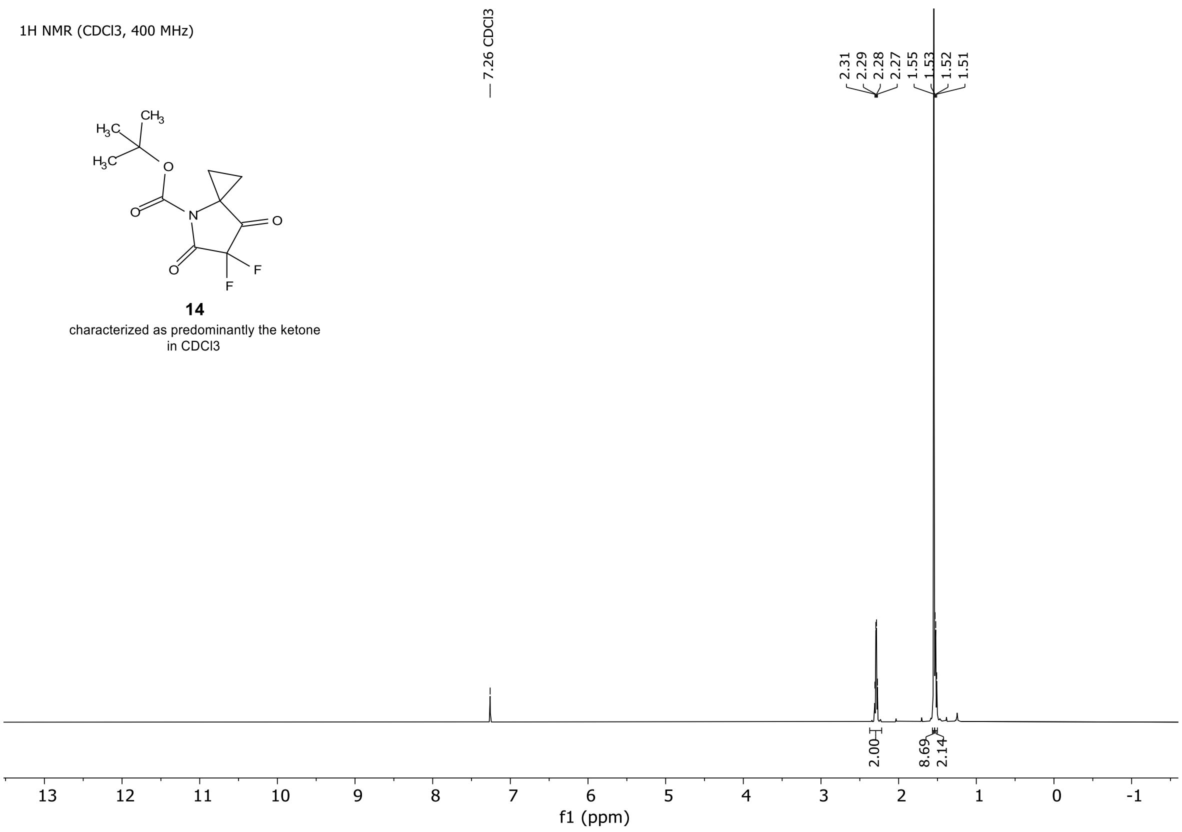

Figure S59: ^{1}H NMR for the characterization of **14**.

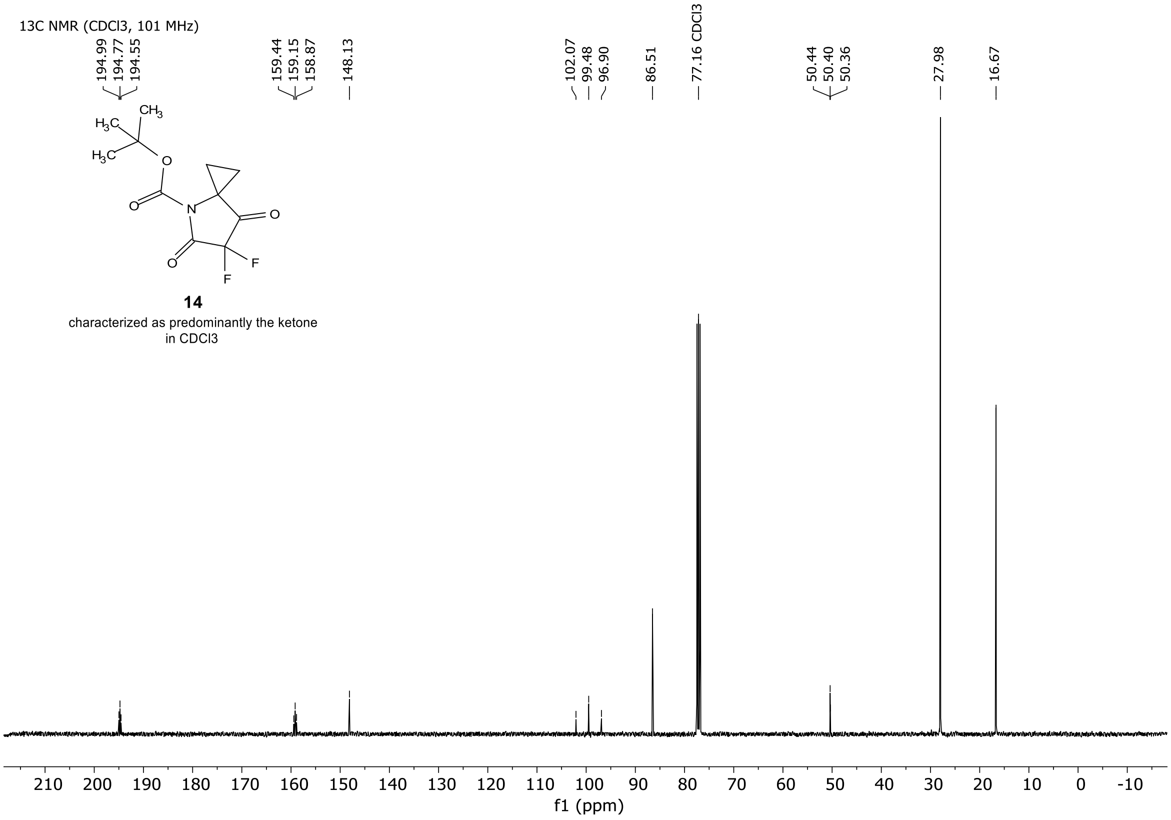

Figure S60: ^{13}C NMR for the characterization of **14**.

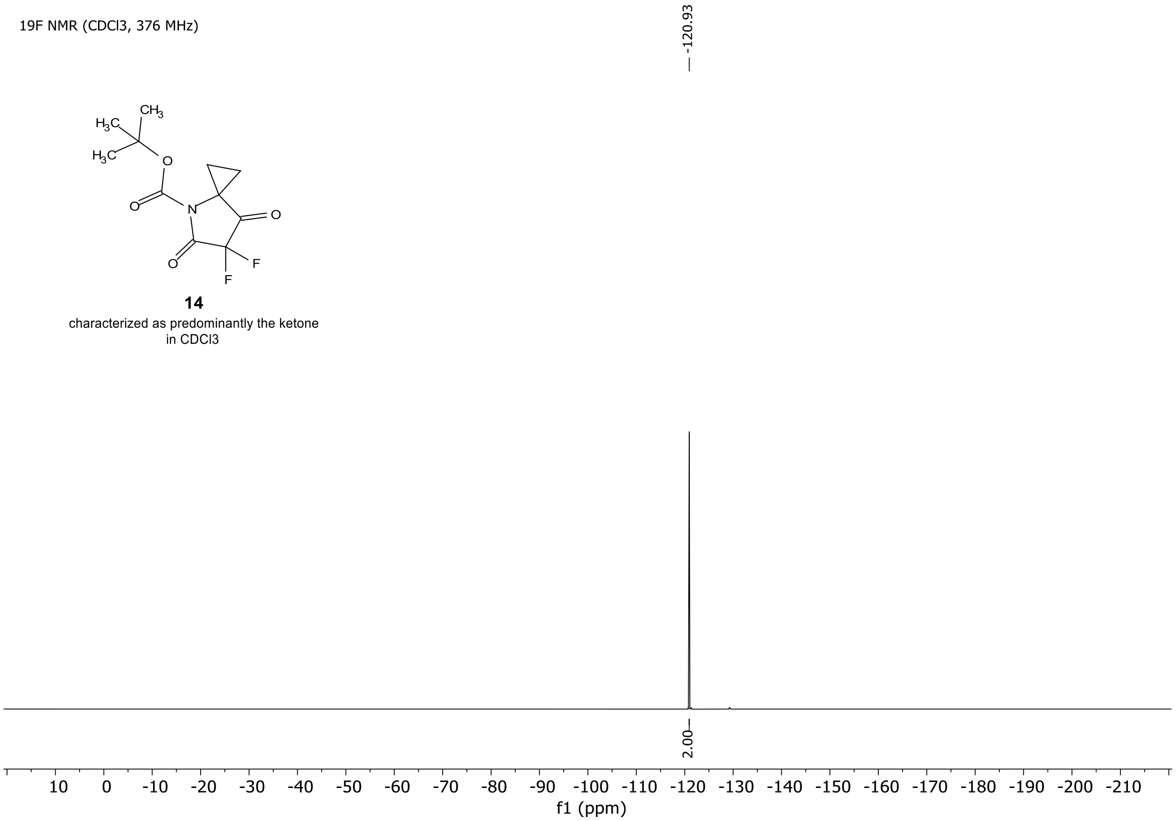

Figure S61: ^{19}F NMR for the characterization of **14**.

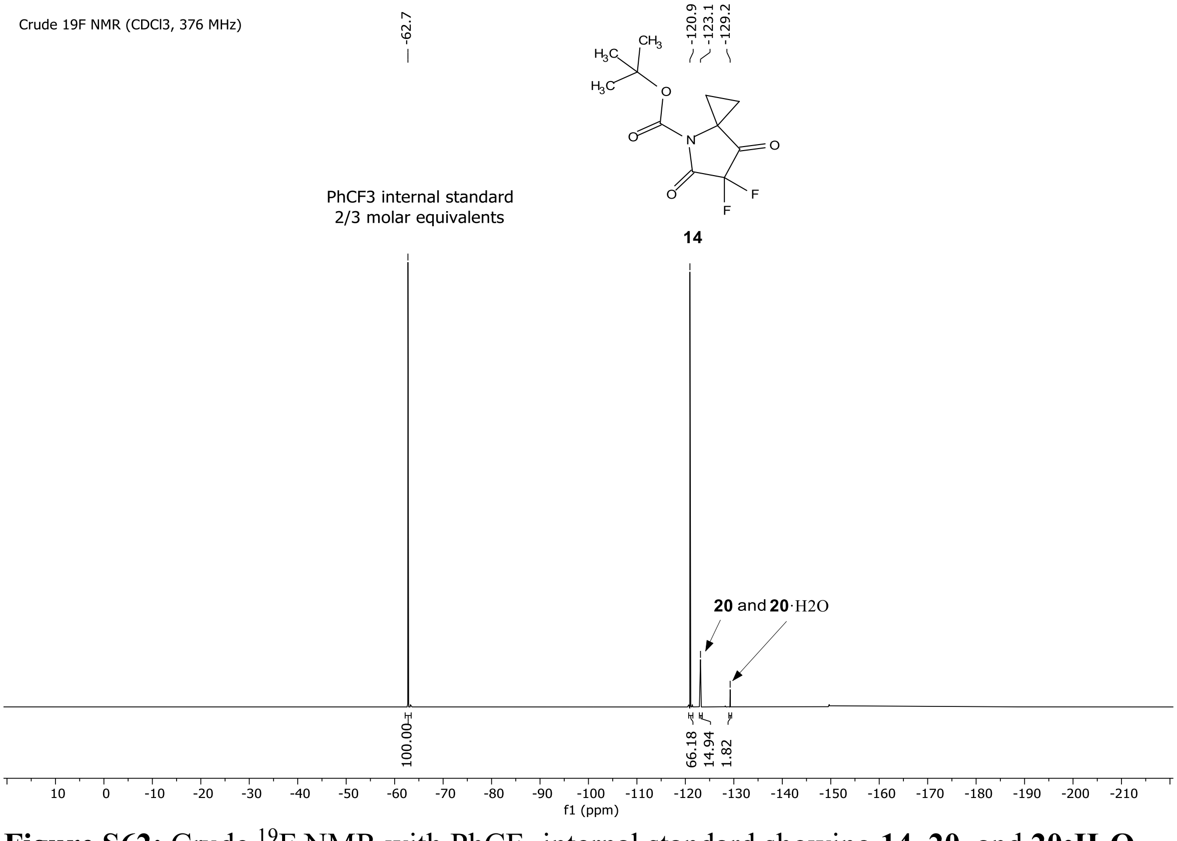

Figure S62: Crude ^{19}F NMR with $PhCF_3$ internal standard showing **14**, **20**, and **20•H$_2$O**.

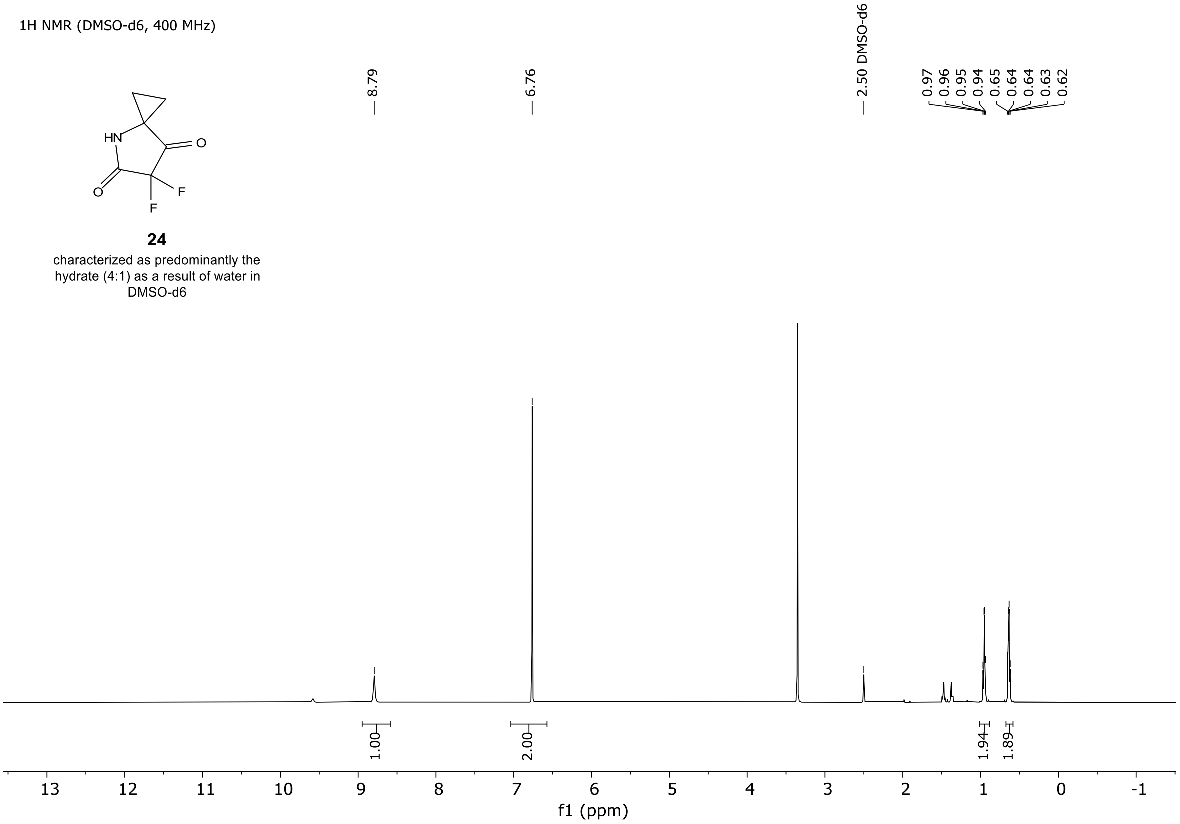

Figure S63: ^{1}H NMR for the characterization of **24**.

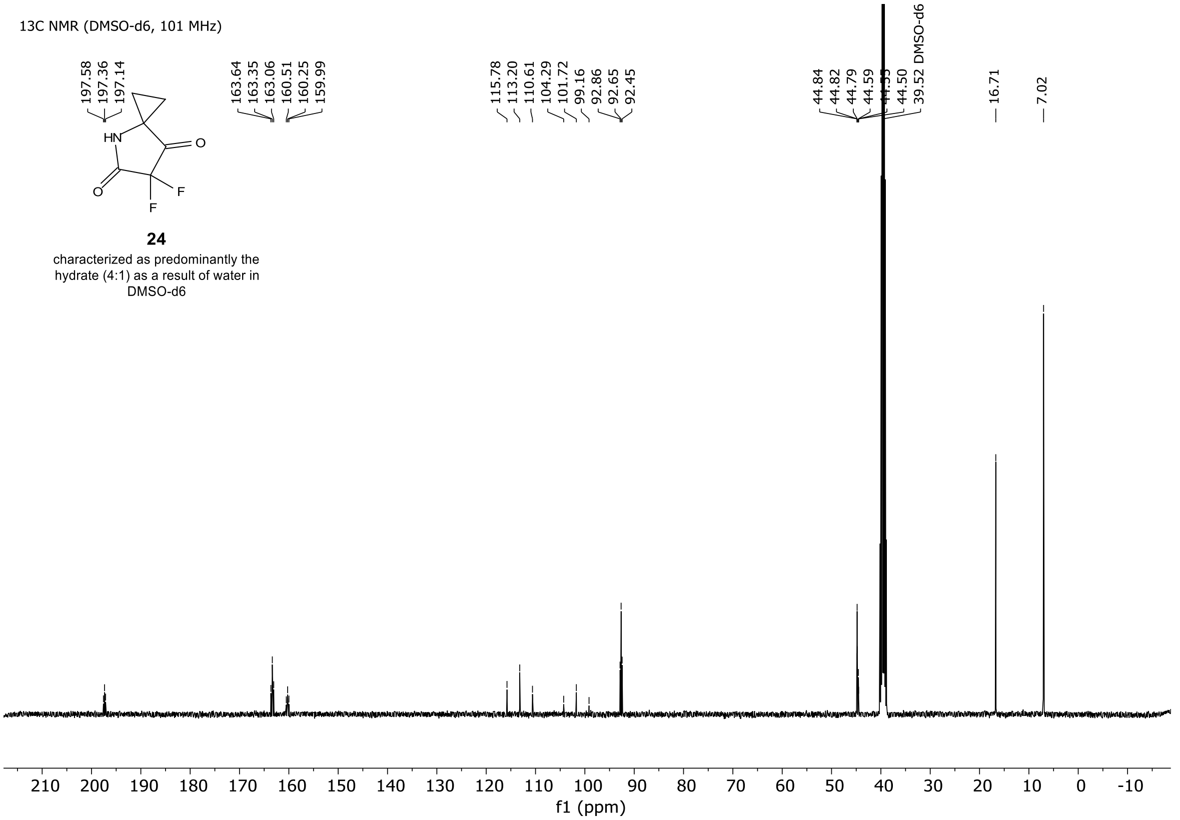

Figure S64: ^{13}C NMR for the characterization of **24**.

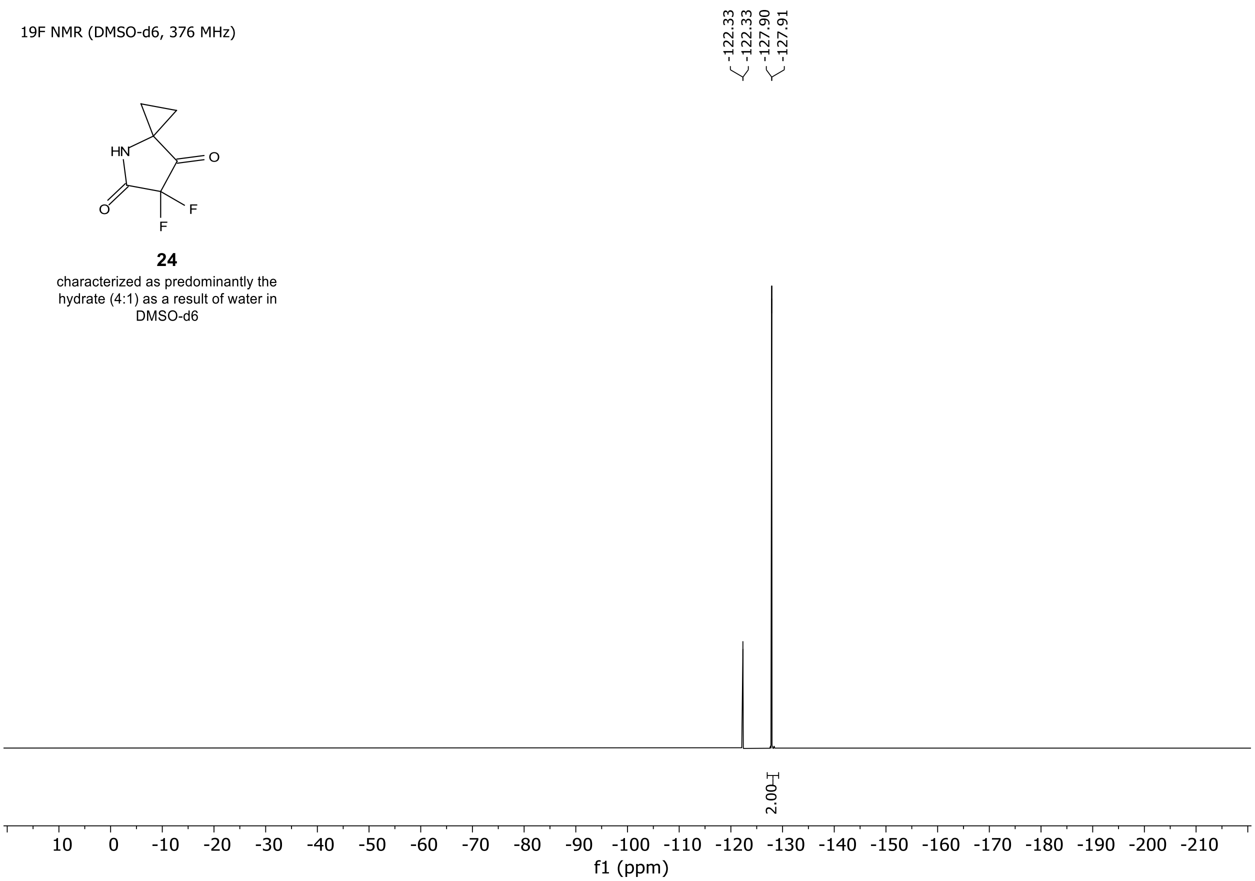

Figure S65: ^{19}F NMR for the characterization of **24**.

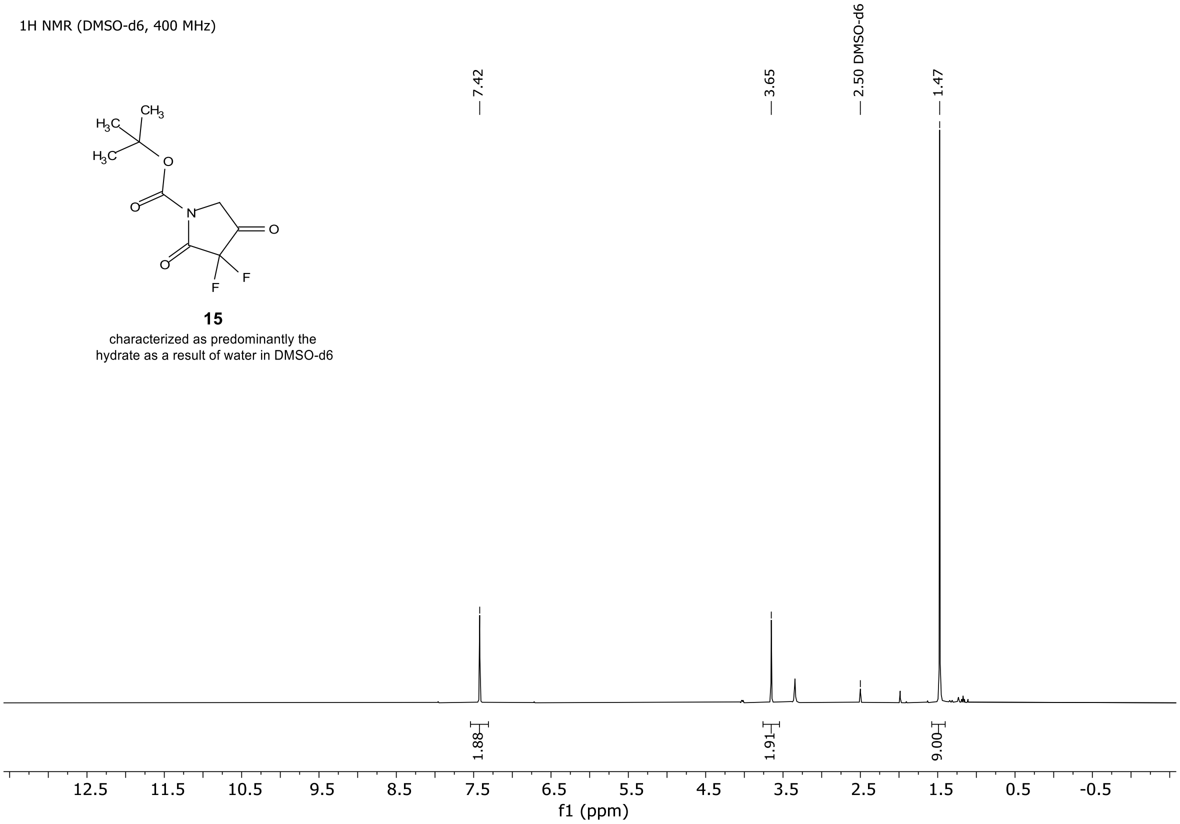

Figure S66: ^{1}H NMR for the characterization of **15**.

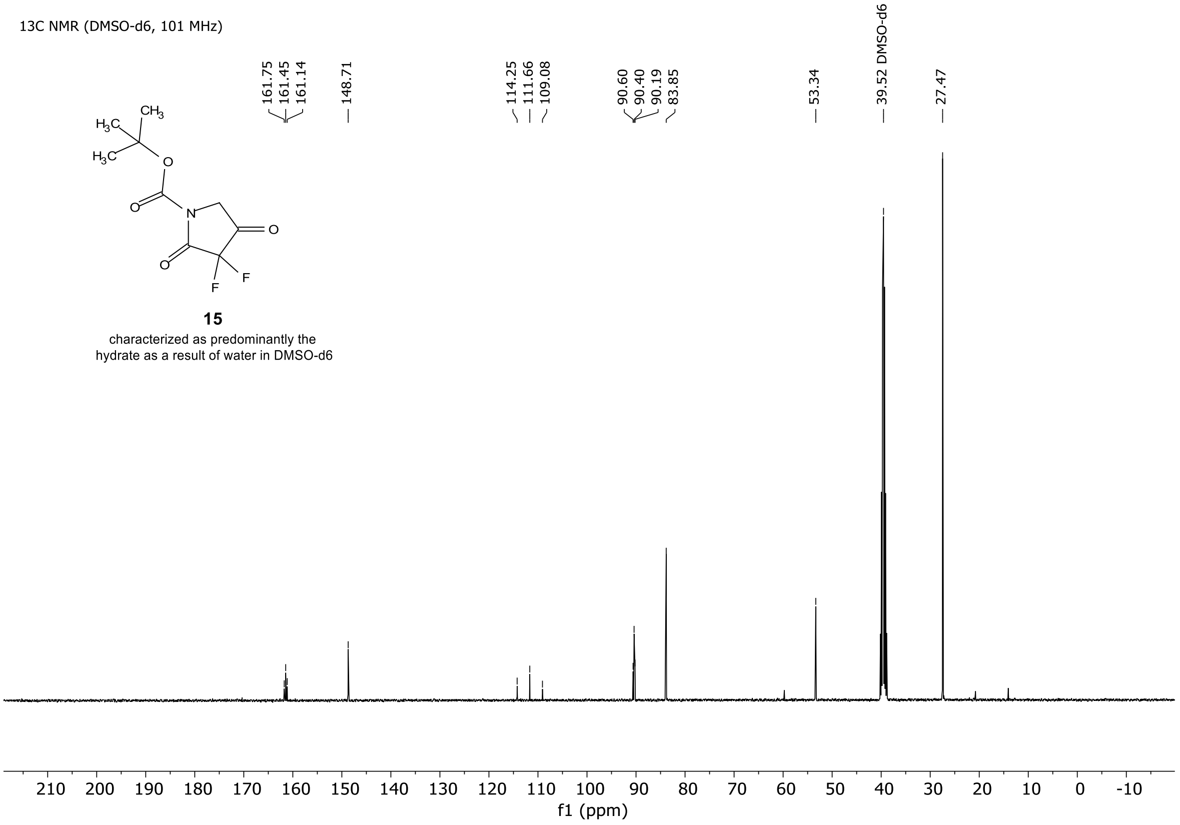

Figure S67: ^{13}C NMR for the characterization of **15**.

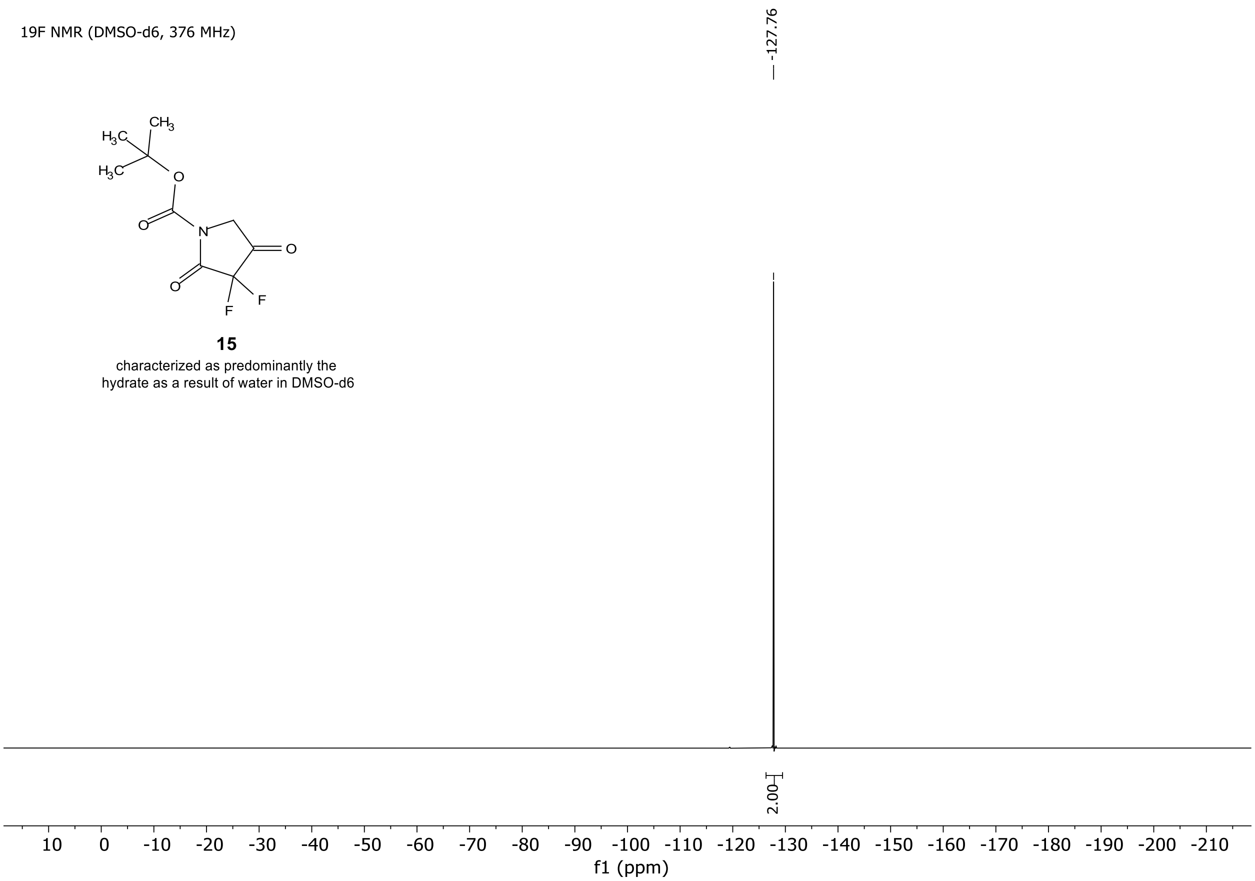

Figure S68: ^{19}F NMR for the characterization of **15**.

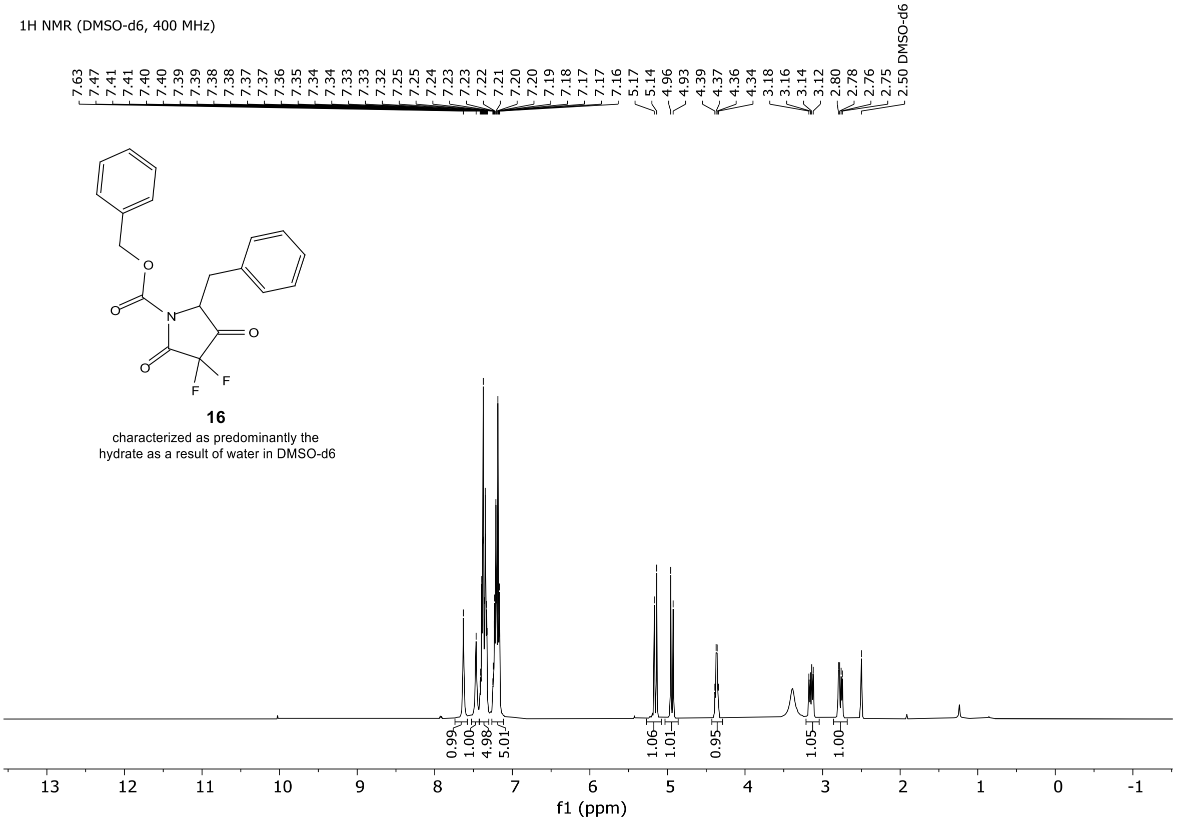

Figure S69: ^{1}H NMR for the characterization of **16**.

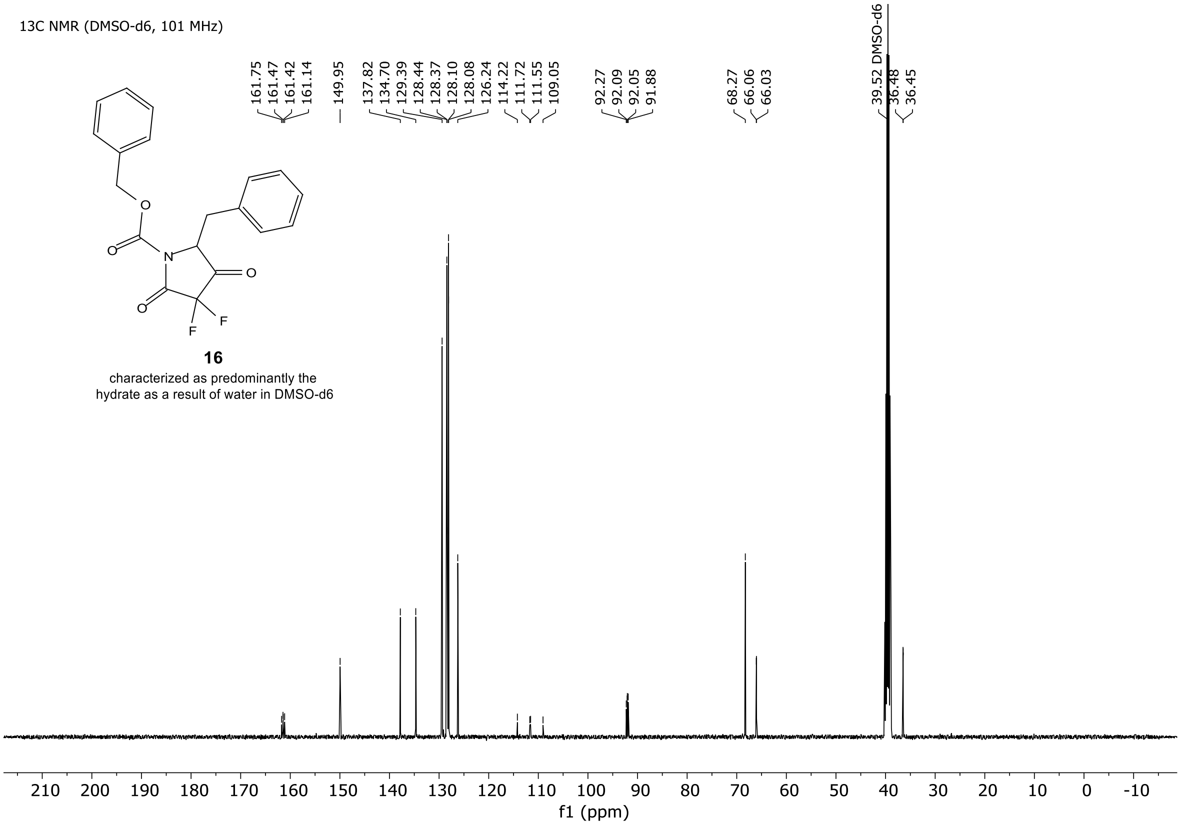

Figure S70: ^{13}C NMR for the characterization of **16**.

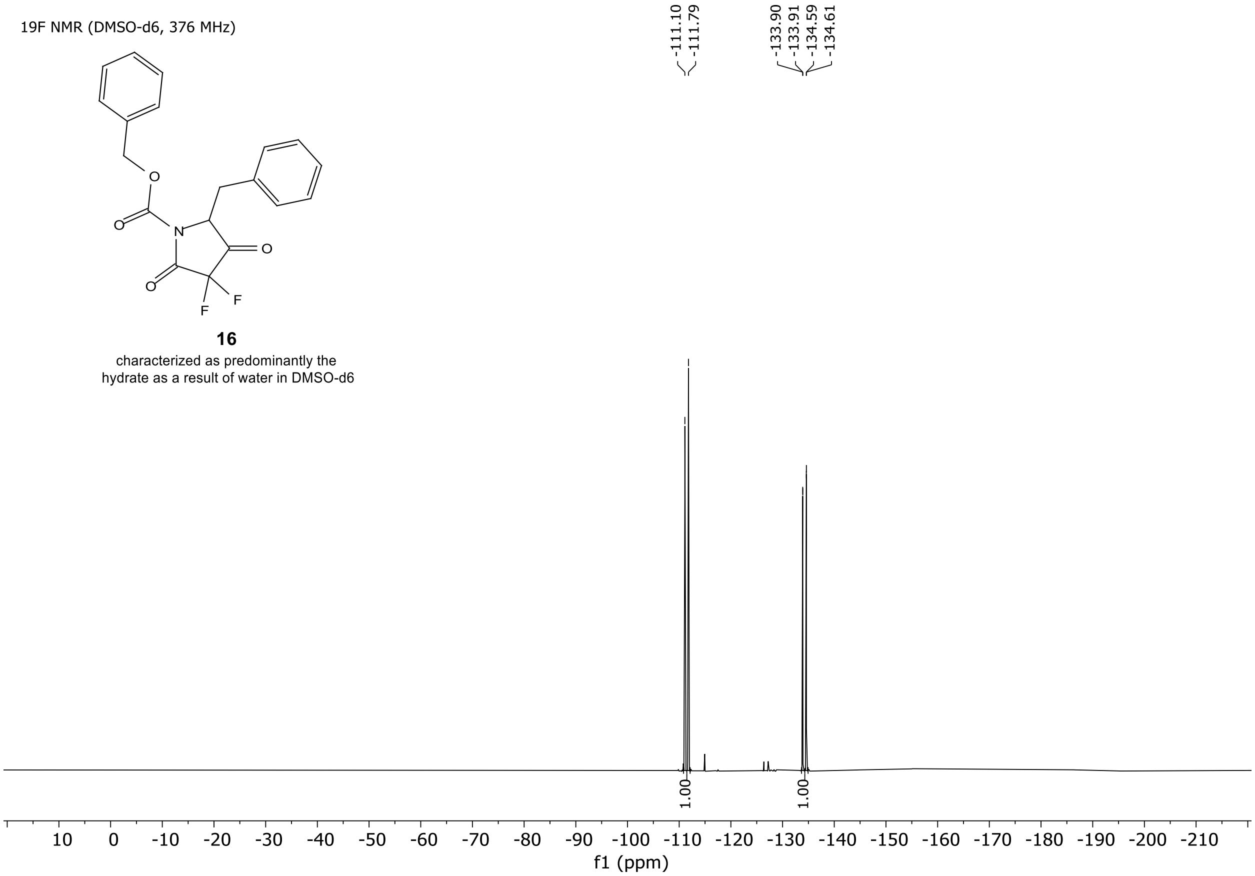

Figure S71: ^{19}F NMR for the characterization of **16**.

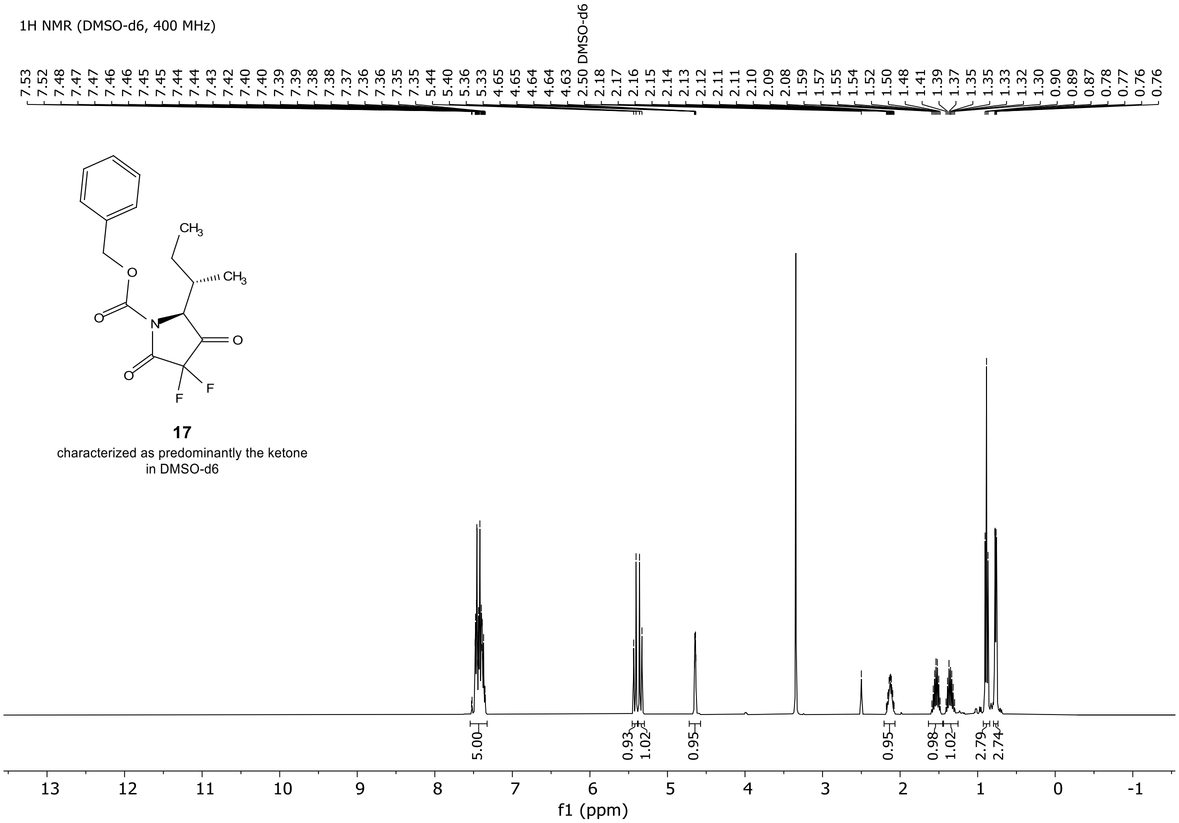

Figure S72: ^{1}H NMR for the characterization of **17**.

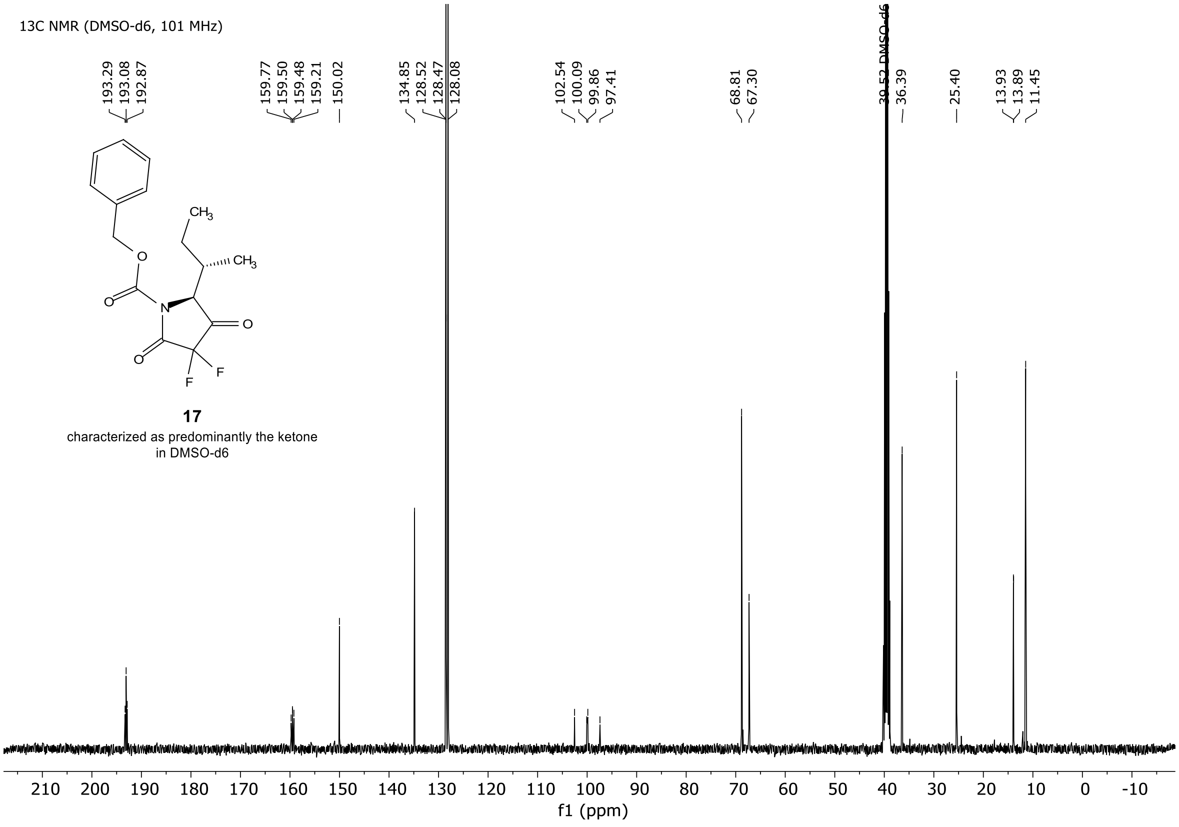

Figure S73: ^{13}C NMR for the characterization of **17**.

19F NMR (DMSO-d6, 376 MHz)

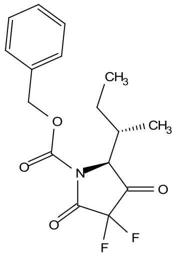

17
characterized as predominantly the ketone
in DMSO-d6

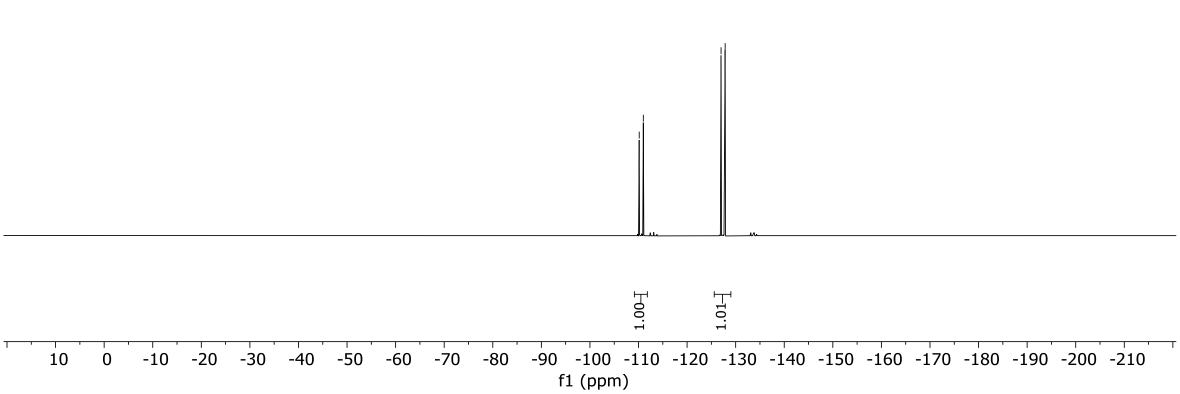

Figure S74: ^{19}F NMR for the characterization of **17**.

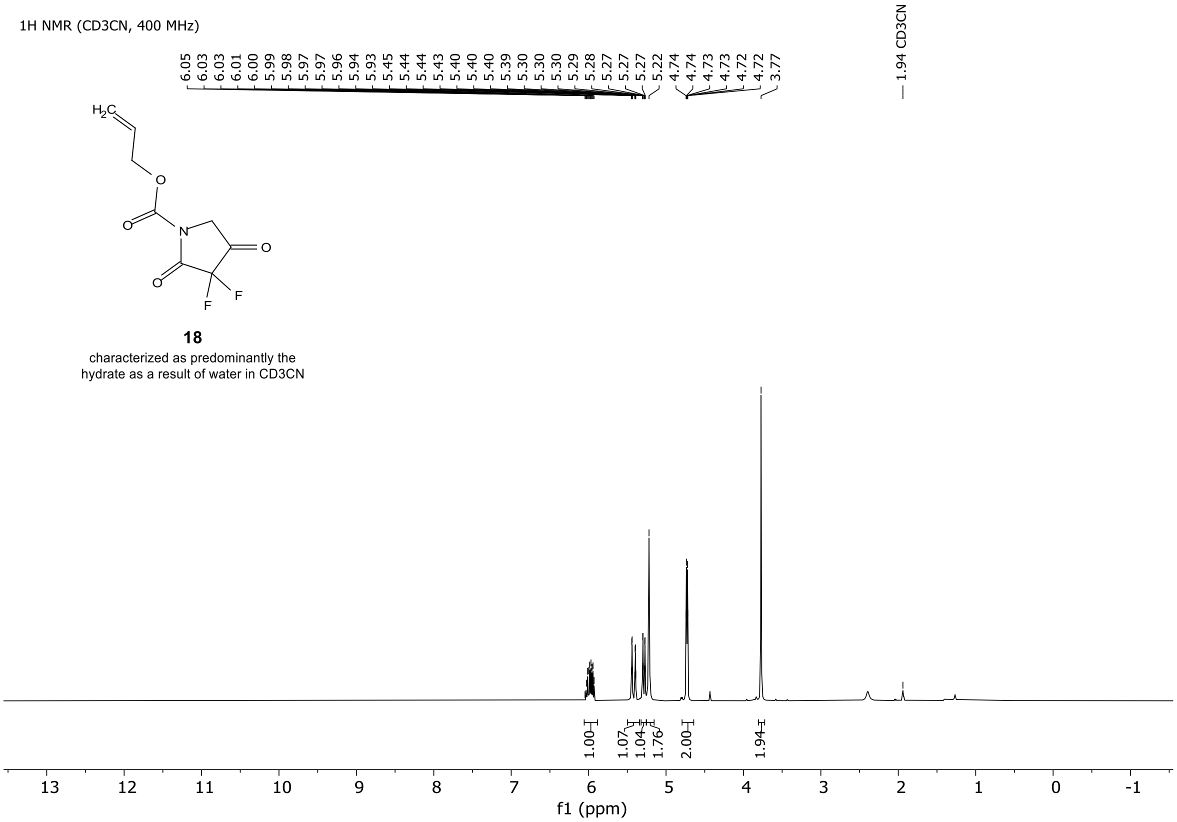

Figure S75: ^{1}H NMR for the characterization of **18**.

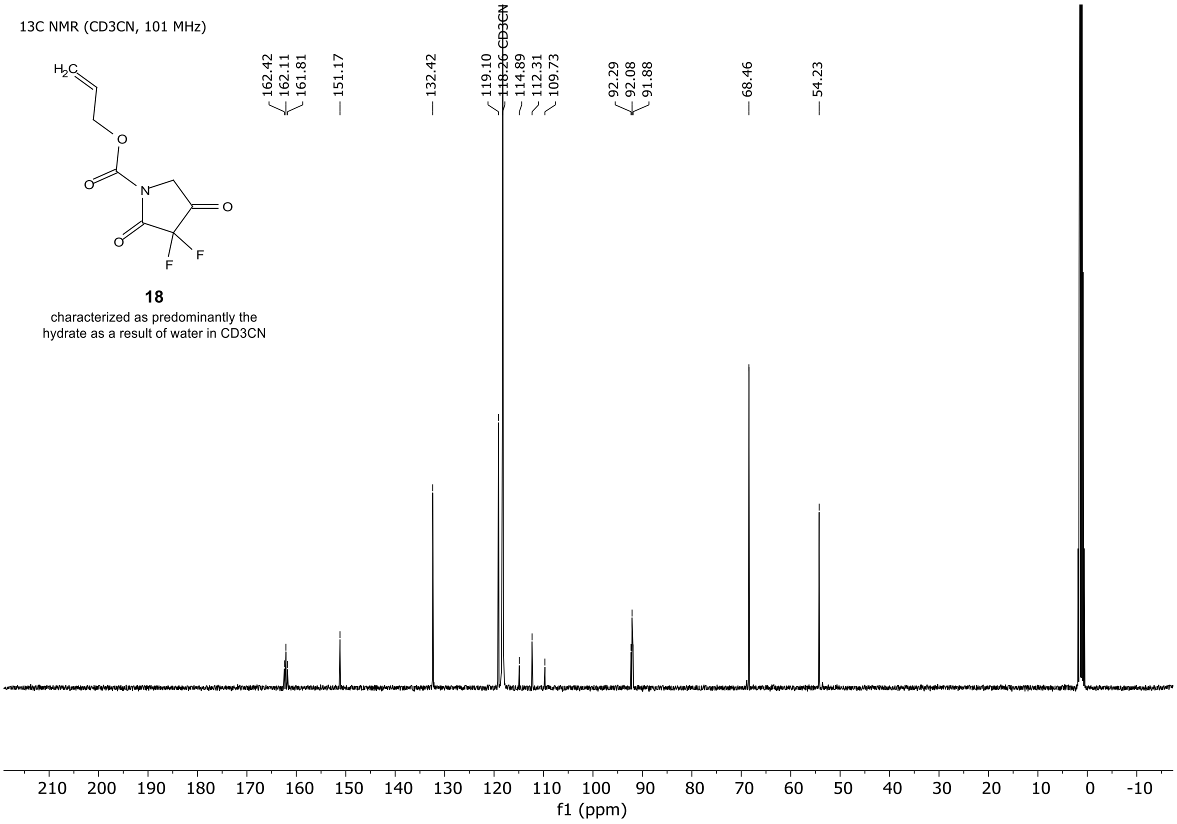

Figure S76: ^{13}C NMR for the characterization of **18**.

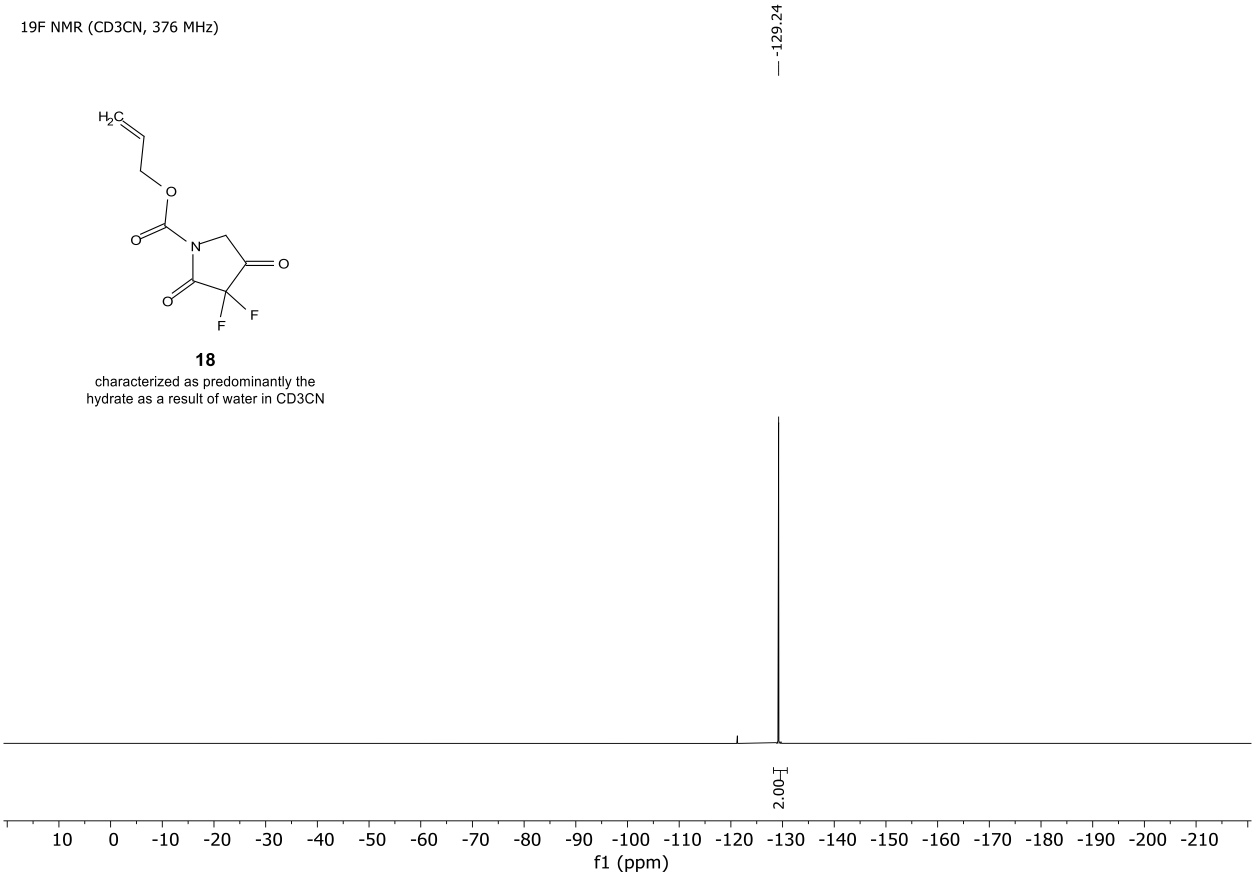

Figure S77: ^{19}F NMR for the characterization of **18**.

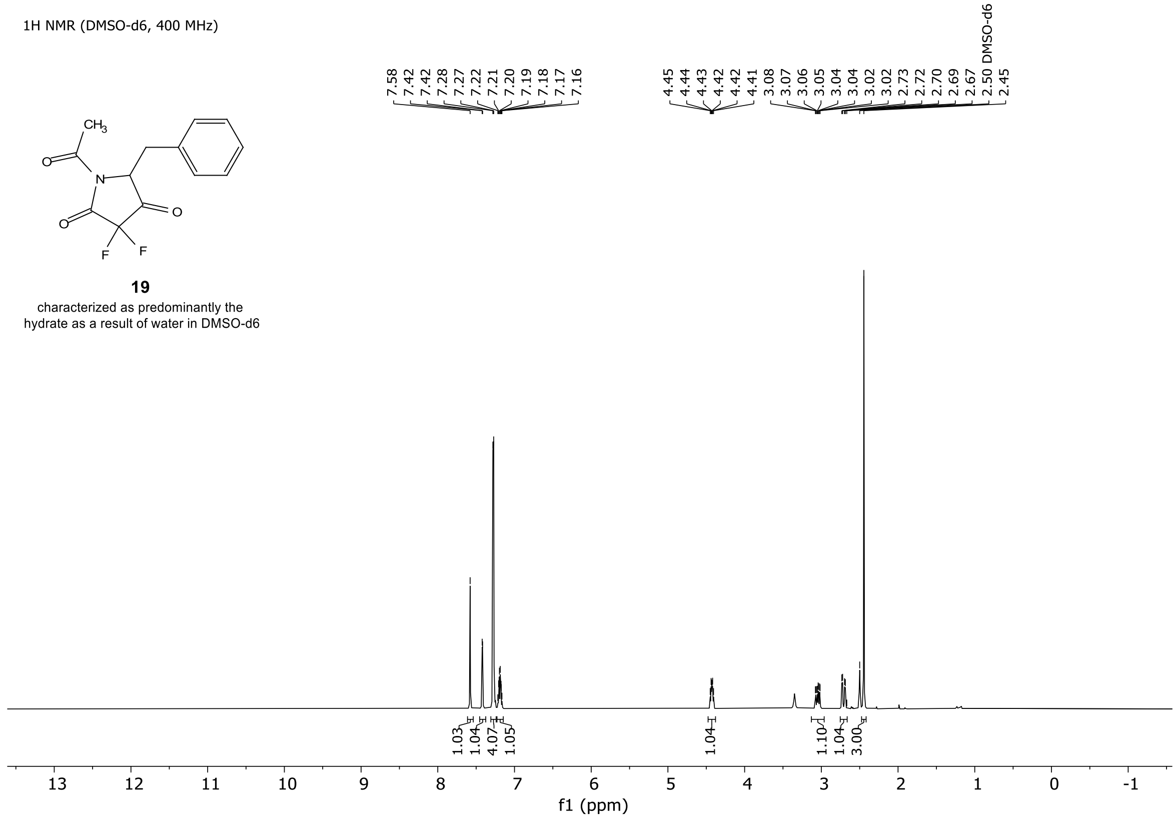

Figure S78: ^{1}H NMR for the characterization of **19**.

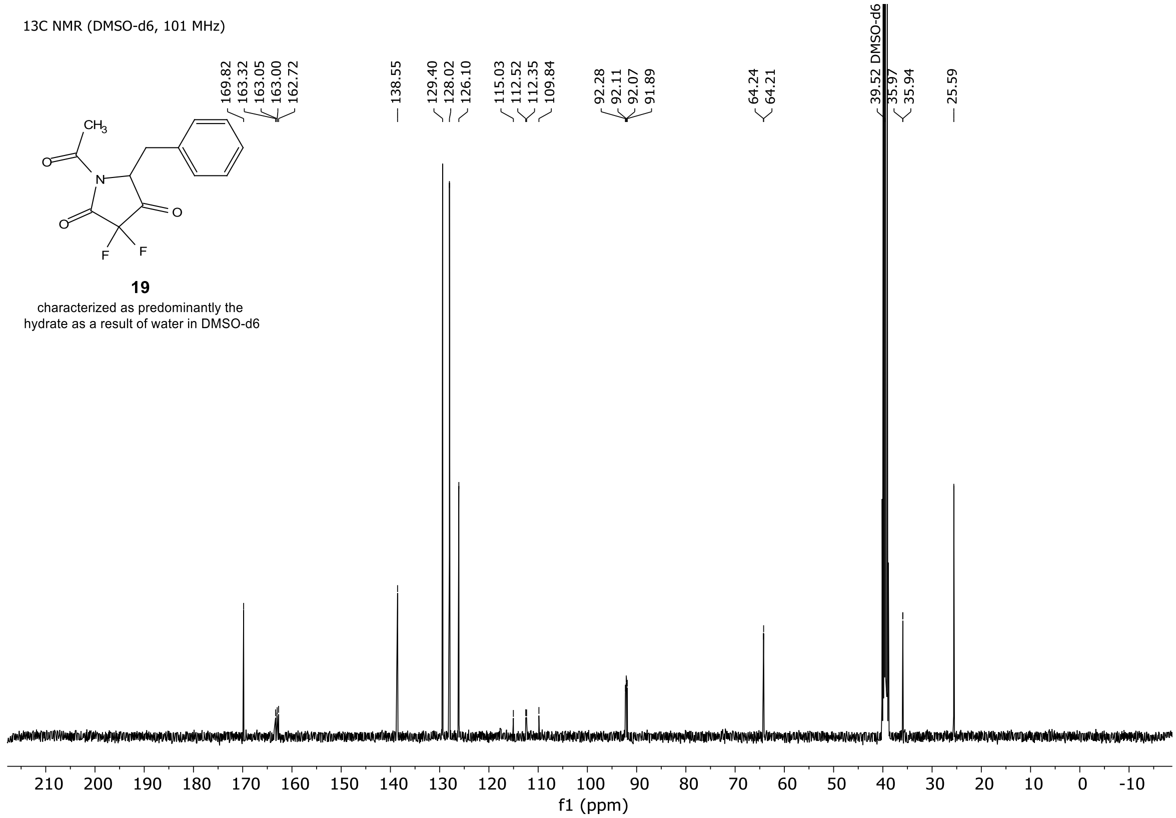

Figure S79: ^{13}C NMR for the characterization of **19**.

19F NMR (DMSO-d6, 376 MHz)

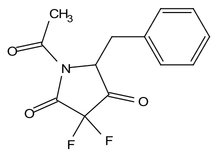

19
characterized as predominantly the hydrate as a result of water in DMSO-d6

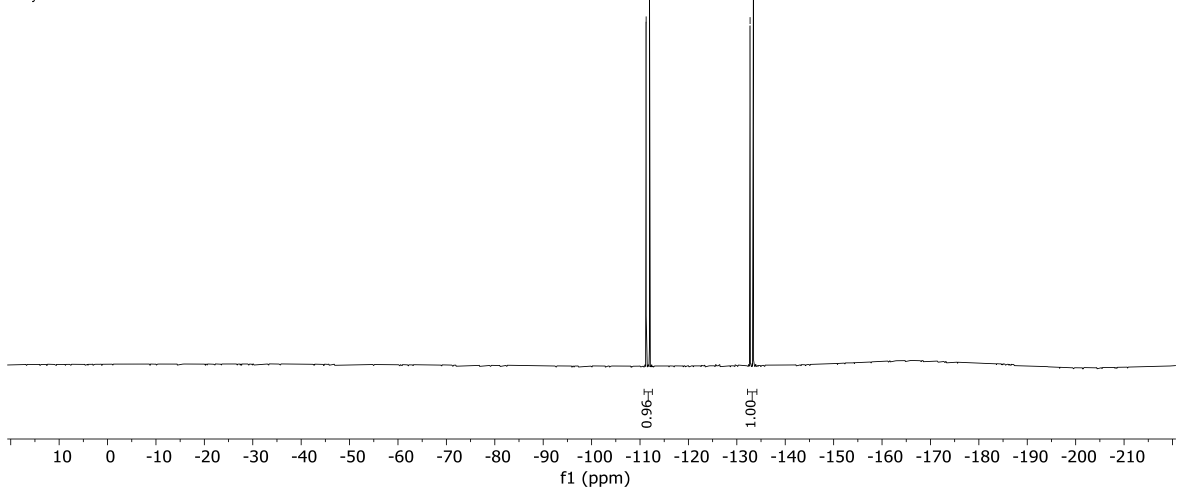

Figure S80: ^{19}F NMR for the characterization of **19**.

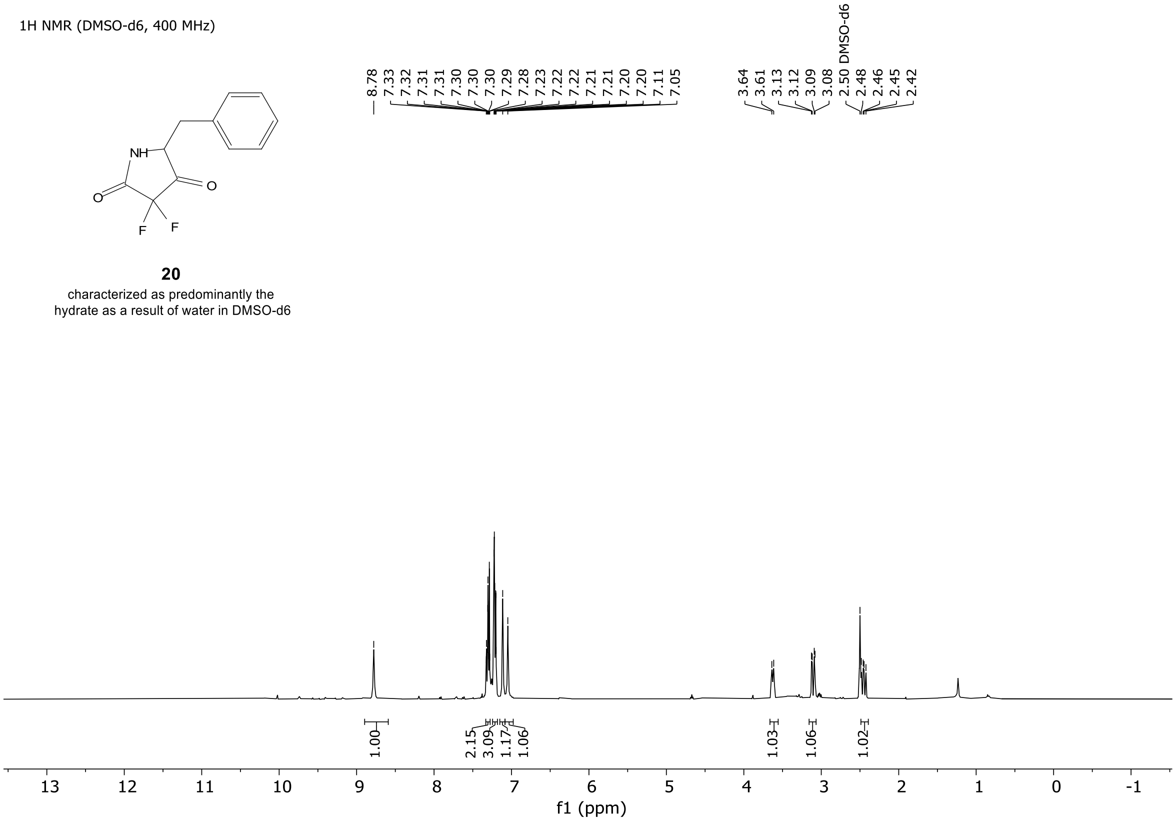

Figure S81: ^{1}H NMR for the characterization of **20**.

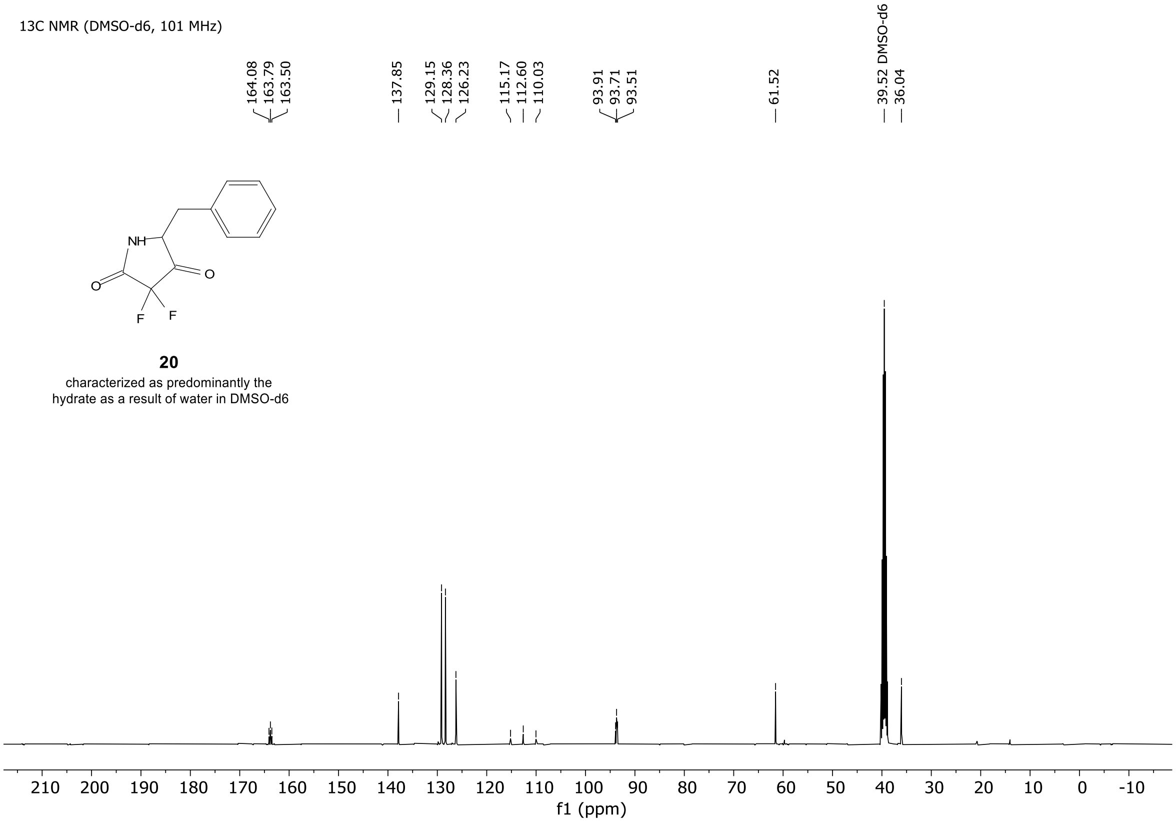

Figure S82: ^{13}C NMR for the characterization of **20**.

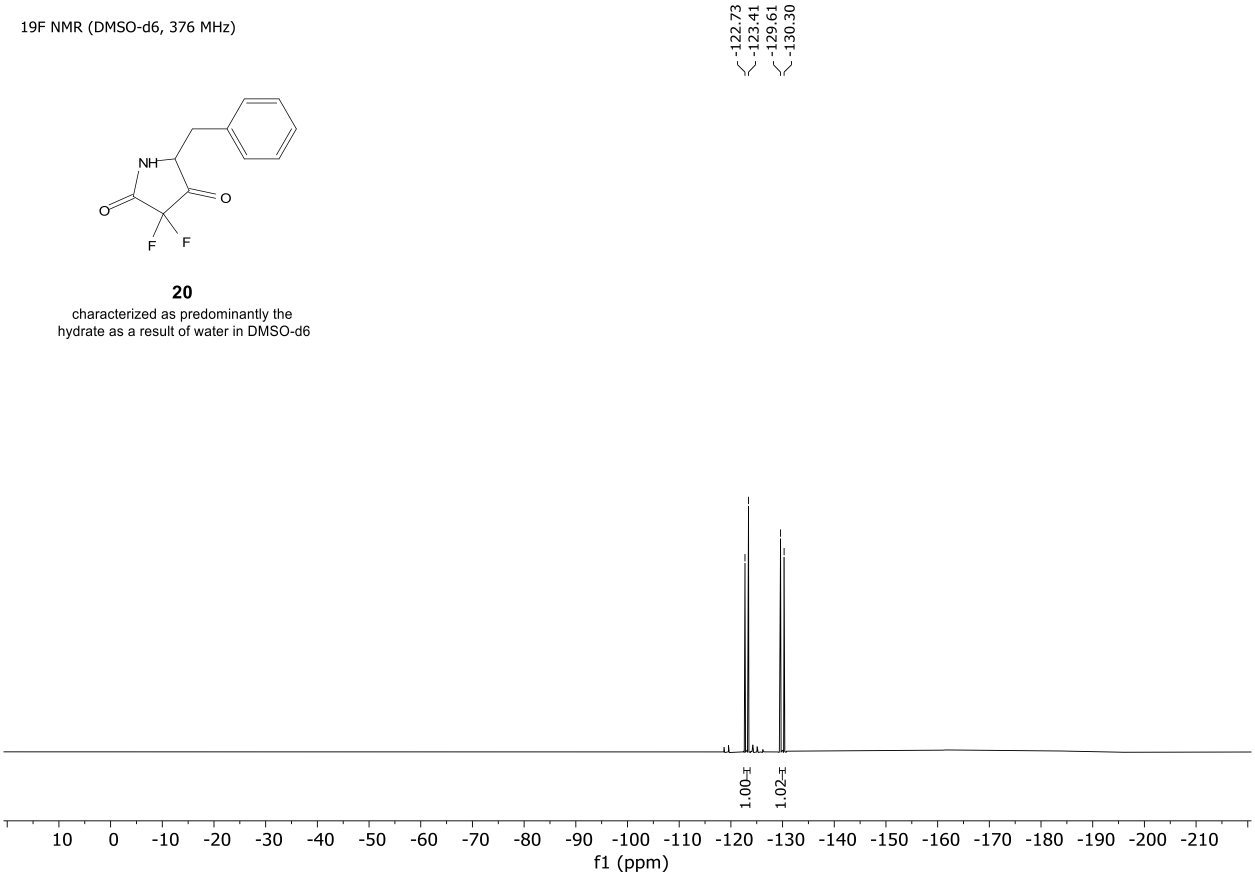

Figure S83: ^{19}F NMR for the characterization of **20**.

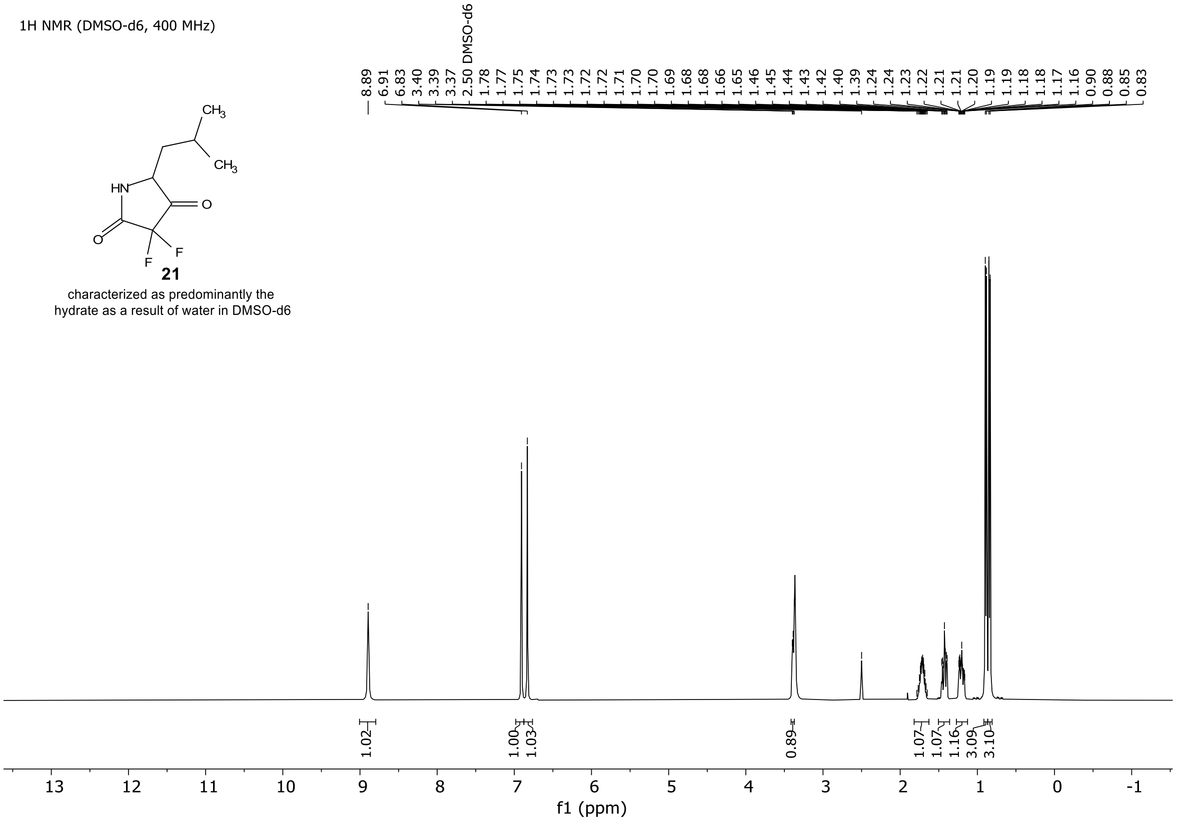

Figure S84: ^{1}H NMR for the characterization of **21**.

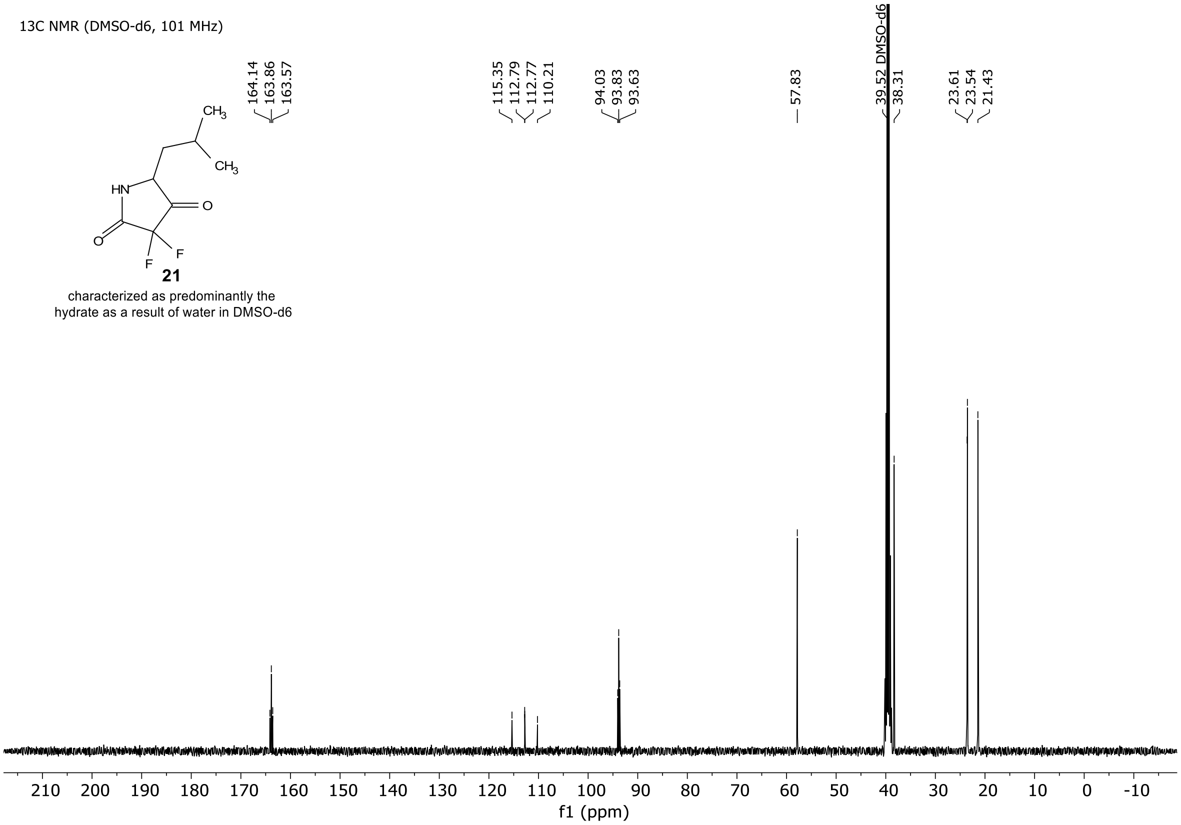

Figure S85: ^{13}C NMR for the characterization of **21**.

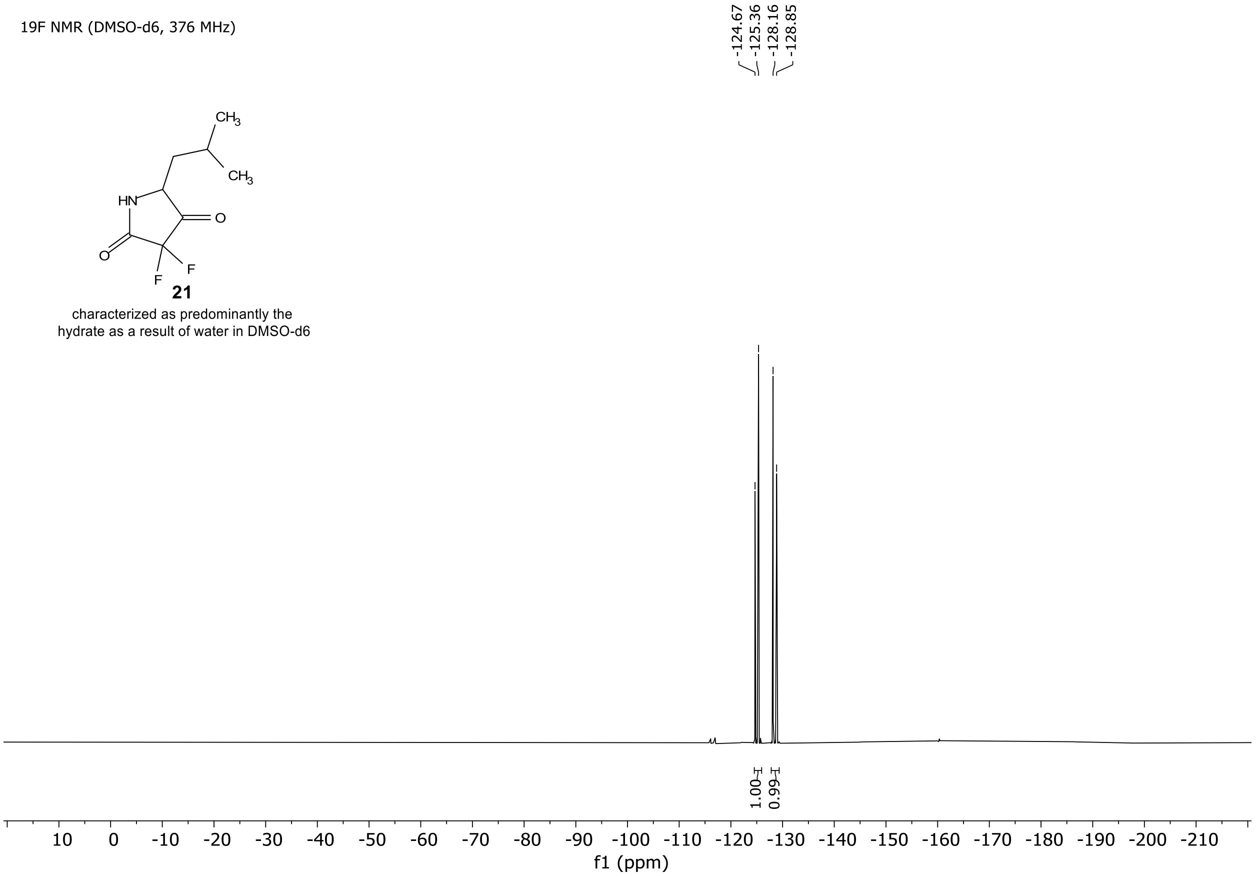

Figure S86: ^{19}F NMR for the characterization of **21**.

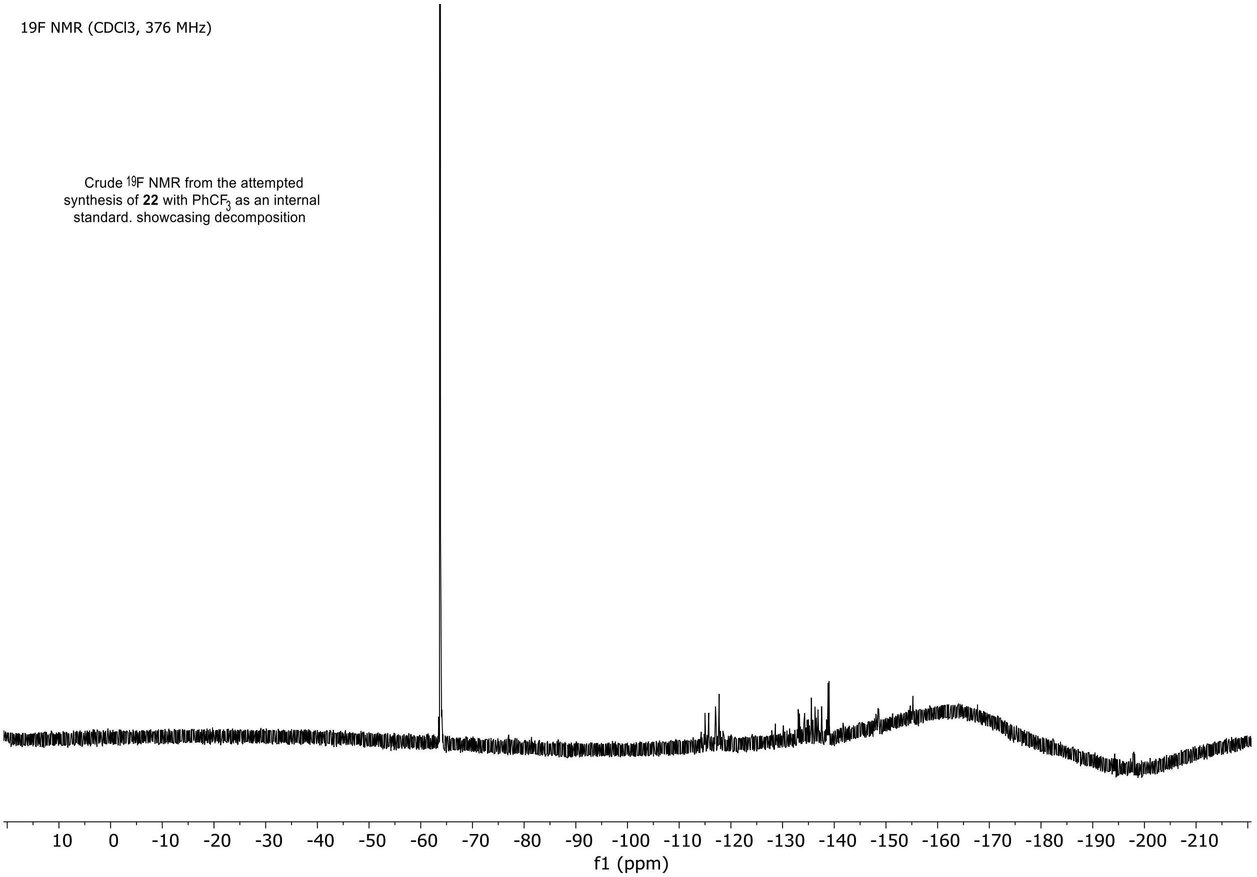

Figure S87: ^{19}F NMR from the attempted synthesis of **22** with $PhCF_3$ as an internal standard.
